# Cytochrome P450-Mediated Metabolism of Antimycobacterial *N*α‑Aroyl‑*N*‑aryl-phenylalanine Amides

**DOI:** 10.1021/acsinfecdis.6c00100

**Published:** 2026-06-02

**Authors:** Johannes Doering, Erik Meerz, Rüdiger W. Seidel, Markus Lang, Andreas M. Kany, Seppo Auriola, Risto Olavi Juvonen, Hannu Raunio, Richard Goddard, Matthew D. Zimmerman, Véronique Dartois, Anna K. H. Hirsch, Thomas Dick, Adrian Richter

**Affiliations:** a Institut für Pharmazie, Martin-Luther-Universität Halle-Wittenberg, Wolfgang-Langenbeck-Str. 4, 06120 Halle (Saale), Germany; b Helmholtz Institute for Pharmaceutical Research Saarland (HIPS), Helmholtz Centre for Infection Research (HZI), Campus Building E8.1, 66123 Saarbrücken, Germany; c PharmaScienceHub, Campus Building A2.3, 66123 Saarbrücken, Germany; d School of Pharmacy, Faculty of Health Sciences, University of Eastern Finland, Box 1627, Kuopio FI-70211, Finland; e Max-Planck-Institut für Kohlenforschung, Kaiser-Wilhelm-Platz 1, 45470 Mülheim an der Ruhr, Germany; f Center for Discovery and Innovation, 3139 Hackensack Meridian Health, Nutley, New Jersey 07110,United States; g Hackensack Meridian School of Medicine, Hackensack Meridian Health, Nutley, New Jersey 07110, United States; h Department of Pharmacy, Saarland University, Campus Building E8.1, 66123 Saarbrücken, Germany; i Department of Microbiology and Immunology, Georgetown University, Washington D.C. 20057,United States

## Abstract

*N*α-Aroyl-*N*-aryl-phenylalanine amides (AAPs) are a class of antimycobacterial substances that inhibit the RNA polymerase and are effective against various pathogenic and opportunistic mycobacteria, including *Mycobacterium tuberculosis*, *Mycobacterium abscessus*, and *Mycobacterium avium*. Further development of these promising compounds, however, has been hindered by their low microsomal stability, leading to insufficient bioavailability. The present study investigates the mechanism by which microsomal enzymes metabolically degrade AAPs and identifies the resulting metabolites using LC-MS/MS. Rapid oxidation of the *ortho*-phenylenediamine structure, present in various substances in this class, plays a key role in this process. Additionally, we demonstrated *in vitro* and *in vivo* that cytochrome P450 enzyme inhibitors significantly slow the degradation of AAPs. Identification of metabolites will inform further chemical modification of AAPs to achieve metabolic stability.

## Graphical abstract

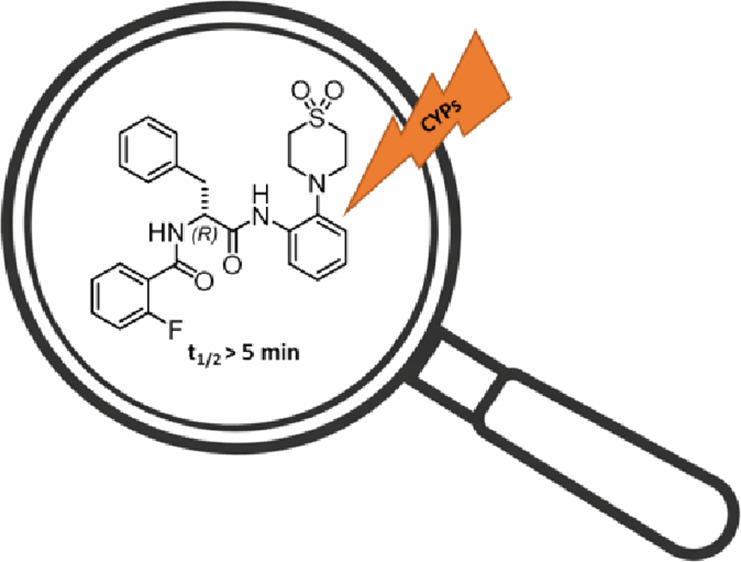

## Introduction

Mycobacterial infections are a global health problem exacerbated by intrinsic and acquired drug resistance of pathogenic and opportunistic mycobacteria.^1,2^ These diseases can be grouped into: tuberculosis, leprosy,^3^ and infections caused by nontuberculous mycobacteria (NTM).^4,5^ Although NTM can cause a wide range of infections, NTM-pulmonary disease is particularly severe and often cannot be adequately treated with currently available drugs.^6,7^ Resistance to clinically used RNA polymerase inhibitors of the rifamycin class (e.g., rifampicin) occurs in opportunistic NTM such as *Mycobacterium abscessus* and *Mycobacterium avium* or multi drug resistant-*Mycobacterium tuberculosis* (MDR-*M. tuberculosis*)/rifamycin resistant-*Mycobacterium tuberculosis* (RR-*M. tuberculosis*) and makes successful therapy considerably difficult.^8–10^ For the opportunistic NTM *M. abscessus* subsp. *abscessus*, the inactivation of rifamycins by adenosine diphosphate (ADP) ribosylation was described as a relevant resistance mechanism.^11,12^ In addition, rifampicin is inactivated by the oxidation of its naphtho-hydroquinone system in *M. abscessus* subsp. *abscessus*. Rifabutin, in contrast, has a naphthoquinone structure instead, resulting in an improved activity against *M. abscessus*.^13^ Acquired resistance to rifamycins in *M. avium* was ascribed to mutations in the *rpoB* gene,^14^ but recent studies have shown that rifampicin is clinically ineffective even against *M. avium* strains that do not possess this mutation.^15,16^ Although the mechanism of RNA polymerase inhibition has great potential against opportunistic and pathogenic mycobacteria,^17,18^ it cannot currently be used effectively against NTM. Furthermore, acquired rifamycin resistance in *M. tuberculosis* is problematical for effective treatment. As a consequence, rifamycin-resistant *M. tuberculosis* is listed in the WHO Bacterial Priority Pathogens List 2024.^19^ In MDR-*M. tuberculosis*, the most prevalent form of rifamycin resistance is induced by mutations in the *rpoB* gene, which encodes the β-subunit of the RNA polymerase. These mutations directly alter the rifamycin binding site (clinically most relevant are D435V, H445Y, and S450L).^20^

*N*α-Aroyl-*N*-aryl-phenylalanine amides (AAPs) are a class of synthetic RNA polymerase inhibitors with antimycobacterial activity that lack cross-resistance to rifamycin owing to their different binding mode to the RNA polymerase.^21^ Motivated by the antimycobacterial activity of the hit compound MMV688845 (GSK1055950A)^22–24^ shown in Figure 1, we carried out a comprehensive characterization of the substance class against different mycobacteria and showed promising properties.^25,26^ In particular, the activity against *M. abscessus*, which is intrinsically resistant against most rifamycins, should be emphasized, and the bactericidal efficacy and the activity in a macrophage-infection model underline the potential of AAPs. Lin et al. showed that the compound D-AAP1 acts as an inhibitor of the RNA polymerase of *M. tuberculosis*. The mechanism of inhibition and the binding site differ from that of rifamycins since AAPs bind to the *N*-terminal part of the bridge-helix and the F-loop thereby inhibiting nucleotide addition.^27^

**Figure 1:**
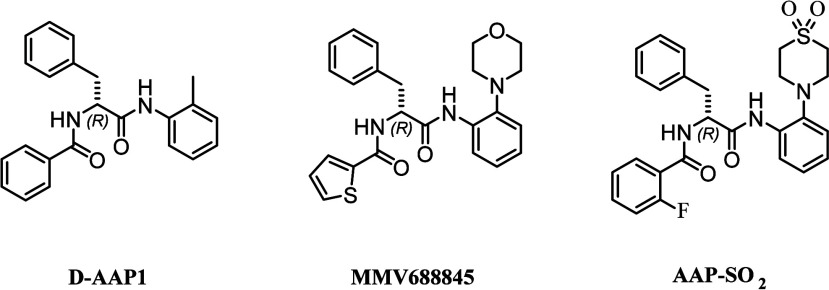
Chemical diagrams of AAPs D-AAP1,^27^ MMV688845,^22^ and AAP-SO_2_.^28^

Isolation of *M. abscessus* mutants resistant to MMV688845 revealed that a mutation in the AAP binding pocket of the RNA polymerase causes resistance to this NTM,^25^ so the target was confirmed for this species of bacteria. Based on a *M. abscessus* homology model of the AAP binding pocket, we performed hit-to-lead optimization affording compound AAP-SO_2_ (Figure 1).^28^ AAP-SO_2_ exhibits an MIC_90_ of 0.78 μM against *M. abscessus* subsp. *abscessus* ATCC19977 and 0.2 μM against *M. tuberculosis* H37Rv. In addition to its efficacy in MIC assays, AAP-SO_2_ showed bactericidal activity against *M. abscessus* and inhibited bacterial growth in macrophage infection assays.^28^ The investigation of AAPs in our group led to a detailed antimycobacterial characterization of this substance class against NTM such as *M. abscessus*, an investigation of structure–activity relationships, and the development of the lead structure AAP-SO_2_. Very recently, the structure of the *M. tuberculosis* RNA polymerase elongation complex bound to AAP-SO_2_, as determined by cryo-electron microscopy (PDB code: 9MRQ), was disclosed.^29^

The promising antimycobacterial properties of AAPs *in vitro* are offset by their low bioavailability and insufficient microsomal stability, although the compounds show satisfactory plasma stability. In a recent study, we showed that methyl or fluorine substituents in the vicinity of the two amide groups can improve the microsomal stability, albeit not sufficiently for adequate *in vivo* bioavailability.^30^ In the present study, we investigated the mechanism of microsomal degradation of the MMV688845 and AAP-SO_2_ and characterized various metabolites detected in LC-MS/MS experiments. In addition, the bioavailability of AAP-SO_2_ was assessed *in vivo* after the inhibition of hepatic metabolism.

## Results and Discussion

### LC-MS/MS Analysis of MMV688845 and AAP-SO_2_ Metabolites in Murine Microsomal Suspensions

In order to develop a strategy for the rational improvement of the metabolic stability of AAPs, we performed LC-MS/MS experiments to identify metabolites and to gain insight into microsomal metabolism (human and murine). For the MMV688845, dealkylation of the morpholine ring was observed, and the formation of metabolite *d* was detected by characteristic mass loss (−70 Da, see Table 1 and Figure 2). The formation of metabolite *d* was validated by the synthesis of a reference compound (see below). In addition to metabolite *d*, three further degradation products (*a*, *b*, and *c*) were detected, which show mass changes at the 2-morpholinoaniline fragment. Both a loss in mass (*a*, – 2 *m*/*z*) and an increase in molecular weight (*b*, + 16 Da, *c*, + 14 Da) were observed.

**Table 1:** MMV688845 Metabolites Determined after Incubation with Murine Liver Microsomes

ID	metabolic reaction	mass diff. (Da)	chemical formula	*m*/*z* obs.	*m*/*z* calc.	mass accuracy (ppm)
**MMV688845**		0	C_24_H_26_N_3_O_3_S^+^	436.1693	436.1689	–0.9
***a***	oxidation	–2	C_24_H_24_N_3_O_3_S^+^	434.1523	434.1533	2.3
***b***	*N*-dealkylation	16	C_24_H_26_N_3_O_4_S^+^	452.1643	452.1639	–0.9
***c***	oxidation + *N*-dealkylation	14	C_24_H_24_N_3_O_4_S^+^	450.1486	450.1482	–0.9
**c** + Na^+^	oxidation + *N*-dealkylation	36	C_24_H_23_N_3_NaO_4_S^+^	472.1293	472.1301	1.7
***d***	*N*-dealkylation	–70	C_20_H_20_N_3_O_2_S^+^	366.1273	366.1271	–0.5
***e***	*N + O*-dealkylation	–26	C_22_H_24_N_3_O_3_S^+^	410.1537	410.1533	–1.0
***f***	hydroxylation	16	C_24_H_26_N_3_O_4_S^+^	452.1645	452.1639	–1.3

**Figure 2:**
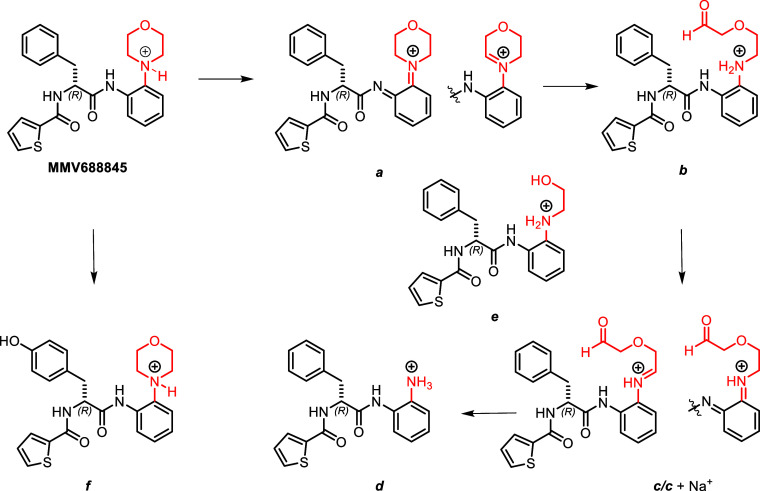
Proposed CYP-mediated metabolic fate of MMV688845 based on *in vitro* studies using murine liver microsomes.

On the basis of the results of the LC-MS/MS analysis, proposed structures for these three metabolites are shown in Figure 2. The first step is likely the oxidation of the *o*-phenylenediamine structure to form an *o*-quinone diimine, which tautomerizes to an iminium cation (metabolite *a*). Hydrolysis of the C=N double bond leads to the formation of metabolite *b* followed by a second oxidation of the *o*-phenylenediamine structure, which again leads to the formation of a hydrolyzable iminium cation (metabolite *c*), the precursor of metabolite *d*. In addition, metabolite *e* with a mass loss of 26 Da at the 2-morpholinoaniline substituent was detected, which indicates the loss of one of the ethylene bridges of the six-membered ring. Metabolite *f* showed an increase in mass of 16 Da on the phenylalanine moiety of the drug, which is probably due to *para*-hydroxylation, which could also be detected for AAP-SO_2_ (see below).

Identification of the metabolites of AAP-SO_2_ revealed a conversion similar to that for MMV688845, as shown in Figure 3 and Table 2. Degradation of the 2-thiomorpholinedioxide moiety and oxidation of the *o*-phenylenediamine unit are characteristic of the metabolism of this AAP: The first step is the oxidation of the *o*-phenylenediamine structure followed by *N*-dealkylation, ultimately leading to metabolite *j*. By the typical mass loss of 118 Da and an independent synthesis, metabolite *j* could be identified unambiguously. It is remarkable that as with MMV688845, a mass loss of 2 Da can be observed for AAP-SO_2_, which indicates the rapid oxidation of the *o*-phenylenediamine structure to *o*-quinone diimine.

**Figure 3:**
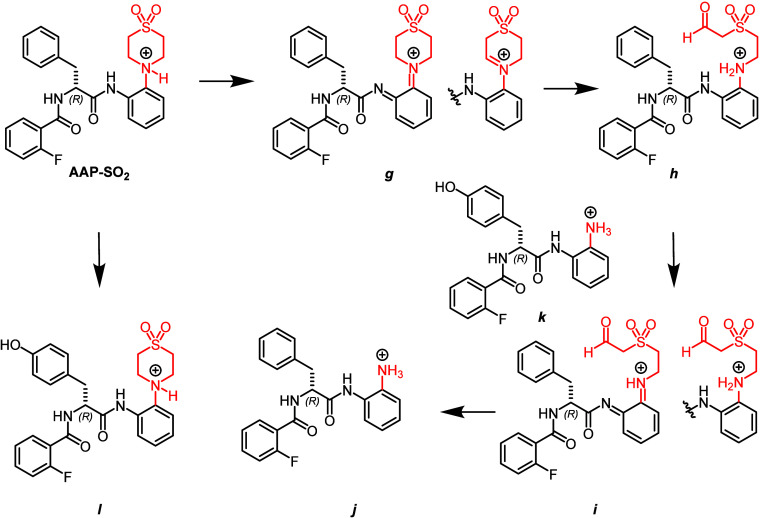
Proposed CYP-mediated pathways for AAP-SO_2_ metabolism based on *in vitro* studies with murine liver microsomes.

**Table 2:** AAP-SO_2_ Metabolites Determined after Incubation with Murine Liver Microsomes

ID	metabolic reaction	mass diff. (Da)	chemical formula	*m*/*z* obs.	*m*/*z* calc.	mass accuracy (ppm)
**AAP-SO**_**2**_		0	C_26_H_27_FN_3_O_4_S^+^	496.1714	496.1701	–2.6
***g***	oxidation	–2	C_26_H_25_FN_3_O_4_S^+^	494.1558	494.1544	2.8
***h***	*N*-dealkylation	16	C_26_H_27_FN_3_O_5_S^+^	512.1662	512.1650	2.3
***i***	oxidation + *N*-dealkylation	14	C_26_H_25_FN_3_O_5_S^+^	510.1508	510.1493	2.9
***j***	*N*-dealkylation	–118	C_22_H_21_FN_3_O_2_^+^	378.1620	378.1612	2.1
***k***	*N*-dealkylation + hydroxylation	–102	C_22_H_21_FN_3_O_3_^+^	394.1570	394.1561	2.3
***l***	hydroxylation	16	C_26_H_27_FN_3_O_5_S^+^	512.1664	512.1650	2.7

In addition to the known *N*-dealkylation, hydroxylation of the phenylalanine side chain was also detected in metabolites *k* and *l*. The identification of compound *l* was possible by the synthesis of a reference compound, which itself represents an active metabolite (see the section below).

### Analysis of Selective Metabolism and Metabolite Identification with Human CYP Enzymes

In order to assess the relevance of metabolite identification for human metabolism, stability studies of AAP-SO_2_ against human microsomes and isolated CYP enzymes (CYP3A4, CYP3A5, and CYP3A7) were performed. Since AAP-SO_2_ and MMV688845 exhibit a very similar behavior toward mouse microsomes, experiments were only conducted with the advanced lead compound. For the experiments, AAP-SO_2_ was incubated for 30 min in a buffer solution containing human microsomes (purchased from BD Biosciences Discovery Labware) and an NADPH regenerating system. The exact conditions are given in the Methods section. Metabolites similar to those identified in the studies of murine liver microsomes could be identified. As shown in Table 3, it is striking that metabolites *g* and *h* known from the murine microsomal metabolism are likewise observed. Different hydroxylation of AAP-SO_2_ is, however, evident (metabolites *m*, *n*, and *o*). The metabolites *m*/*n* show a mass increase of 32 Da, which indicates double hydroxylation. One hydroxylation occurs on the phenylalanine side chain and the other on the *o*-phenylenediamine, although the exact positions of the hydroxylation could not be determined with certainty. Compound *o* is a monohydroxylated metabolite in which the *o*-phenylenediamine moiety has also been oxidized to an *o*-quinone diimine, resulting in a mass increase of 14 Da. Figure 4 shows structural proposals for the detected metabolites, although the exact position of the hydroxylation could not be accurately located in all cases. Metabolite *g*, in which the *o*-phenylenediamine structure was oxidized to a quinone diimine, is particularly prominent. As shown in Figure 5, metabolite *g* could be detected during incubation with microsomes and also after incubation with cytochromes CYP3A4, CYP3A5, and CYP3A7.

**Table 3:** AAP-SO_2_ Metabolites Determined after Incubation with Human Liver Microsomes

ID	metabolic reaction	mass diff. (Da)	chemical formula	*m*/*z* obs.	*m*/*z* calc.	mass accuracy (ppm)
**AAP-SO**_**2**_		0	C_26_H_27_FN_3_O_4_S^+^	496.1698	496.1701	0.6
***g***	oxidation	–2	C_26_H_25_FN_3_O_4_S^+^	494.1552	494.1544	–1.6
***l***	hydroxylation	16	C_26_H_27_FN_3_O_5_S^+^	512.1646	512.1650	0.8
***h***	*N*-dealkylation	16	C_26_H_27_FN_3_O_5_S^+^	512.1648	512.1650	0.4
***m***	hydroxylation	32	C_26_H_27_FN_3_O_6_S^+^	528.1595	528.1599	0.8
***n***	hydroxylation	32	C_26_H_27_FN_3_O_6_S^+^	528.1593	528.1599	1.1
***o***	oxidation/hydroxylation	14	C_26_H_25_FN_3_O_5_S^+^	510.1493	510.1493	0.0

**Figure 4:**
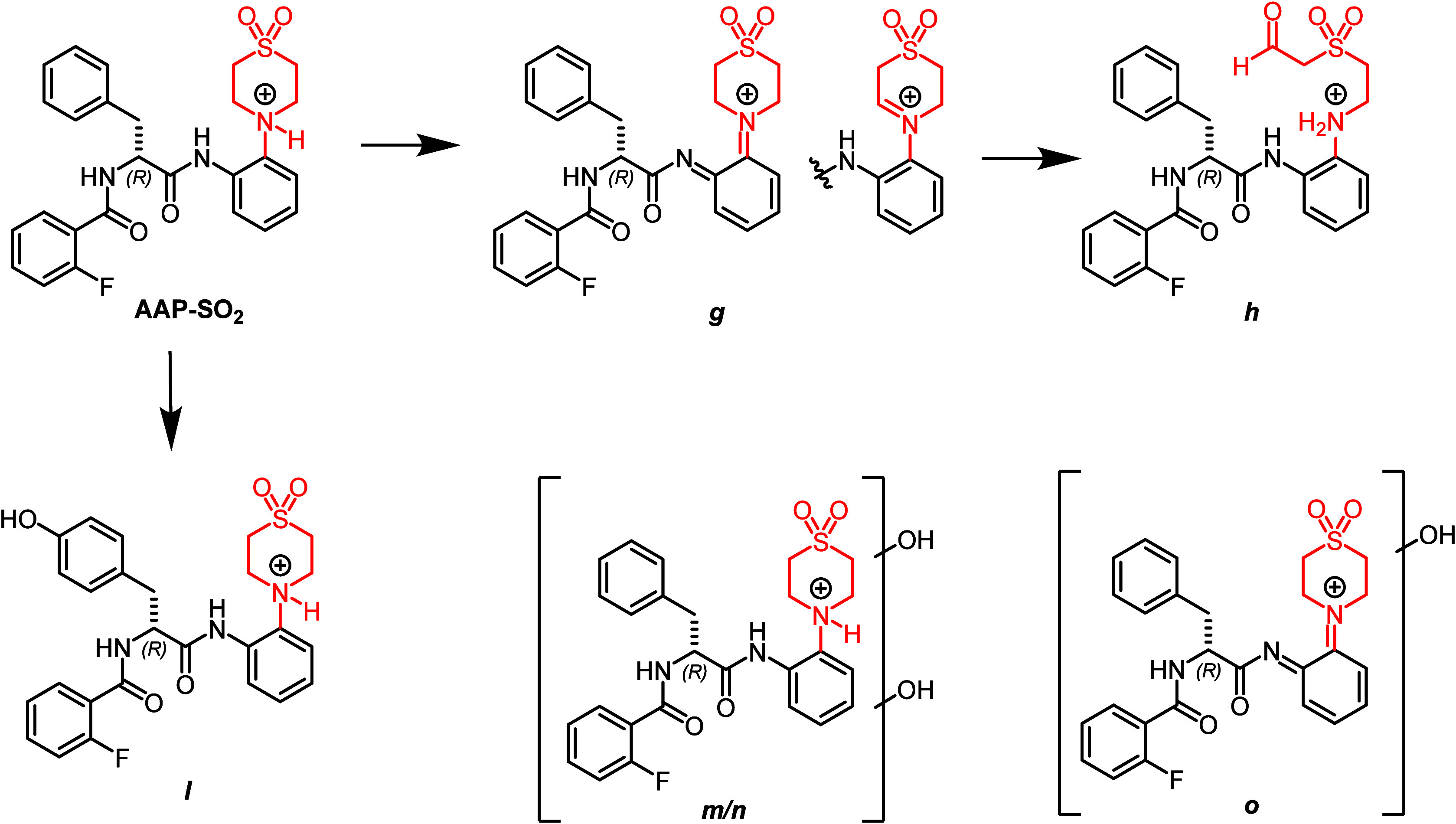
Proposed CYP-mediated pathways for AAP-SO_2_ metabolism based on *in vitro* studies with human liver microsomes. The positions of the hydroxy groups in m/n and o could not be determined.

**Figure 5:**
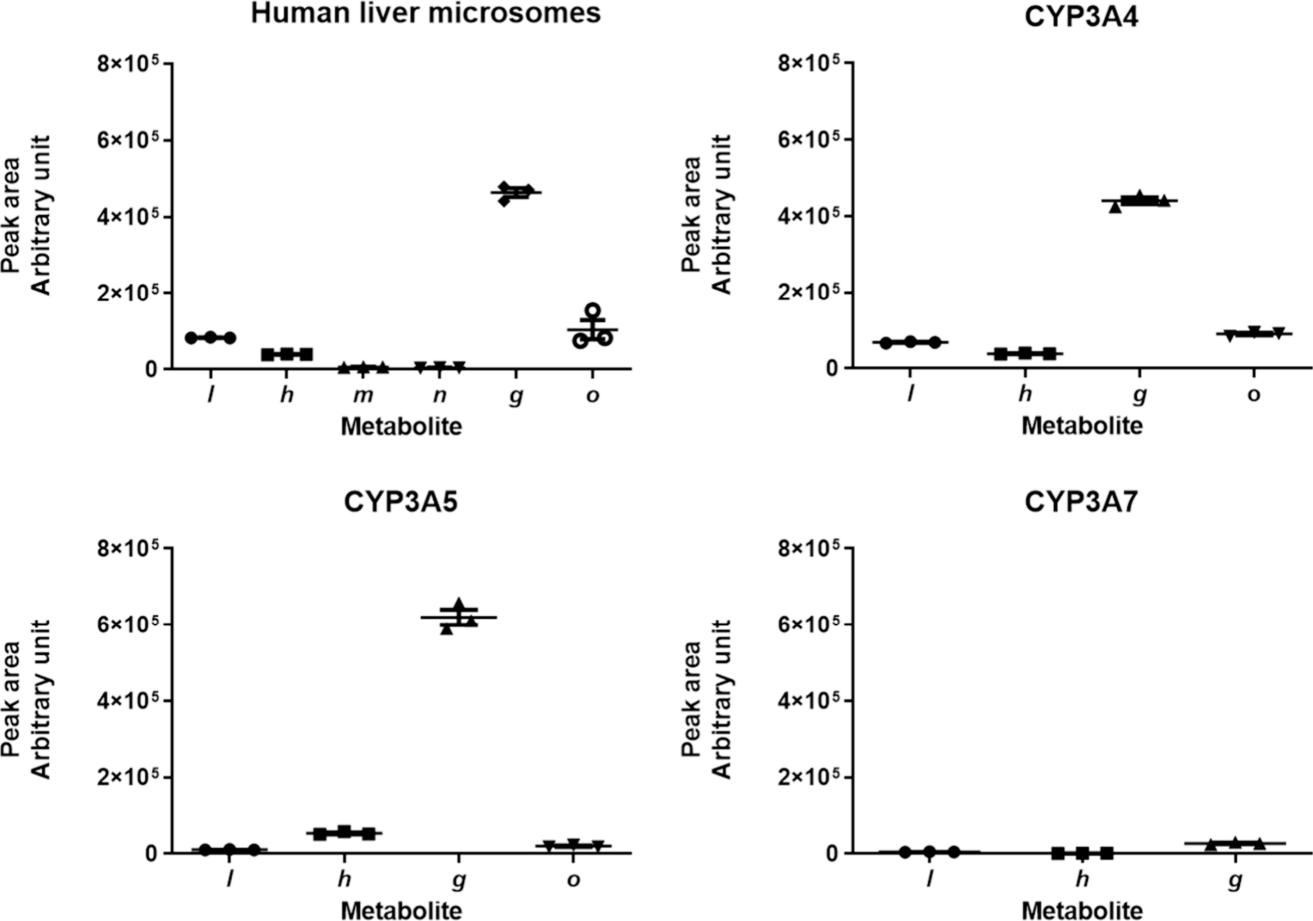
LC/MS-MS analysis of oxidation products of AAP-SO_2_ in incubations, with human microsomes or CYP enzymes. Samples were incubated for 30 min in triplicate.

Estimation of the relative amounts of the respective metabolites based on the arbitrary unit peak area of the LC-MS experiments reveals that metabolite *g* is formed when incubated with various CYP enzymes (see Figure 5). Thus, the data suggest that the *o*-phenylenediaminein AAPs is rapidly oxidized by human CYP3A enzymes.

In addition to the prominent appearance of metabolite *g*, it is noticeable that metabolites *m* and *n* cannot be observed during conversion with isolated CYP3A enzymes. The formation of metabolites *l*, *g*, *h*, and *o* by both CYP3A4 and CYP3A5 indicates a CYP-mediated degradation of the 2-thiomorpholin-2-one-aniline system. This metabolism is also observed with CYP3A7 but without the formation of metabolite *o*, which is hydroxylated.

### Synthesis and Characterization of AAP Metabolites

A selection of metabolites of MMV688845 and AAP-SO_2_ was synthesized using the synthetic procedure shown in Figure 6. The synthesis of the metabolite *e* started with a nucleophilic aromatic substitution of o-bromonitrobenzene with ethanolamine followed by the hydrogenation of the nitro group using a palladium catalyst resulting in **IM1** (see Figure 6A below). The resulting amine **IM1** was then coupled in the next step with *N*-Boc-d-phenylalanine, leading to **IM2**. Subsequently, the Boc protecting group was cleaved under acidic conditions and another amide bond was formed using the coupling reagent PyBOP,^31^ which led to the formation of metabolite *e*.

**Figure 6:**
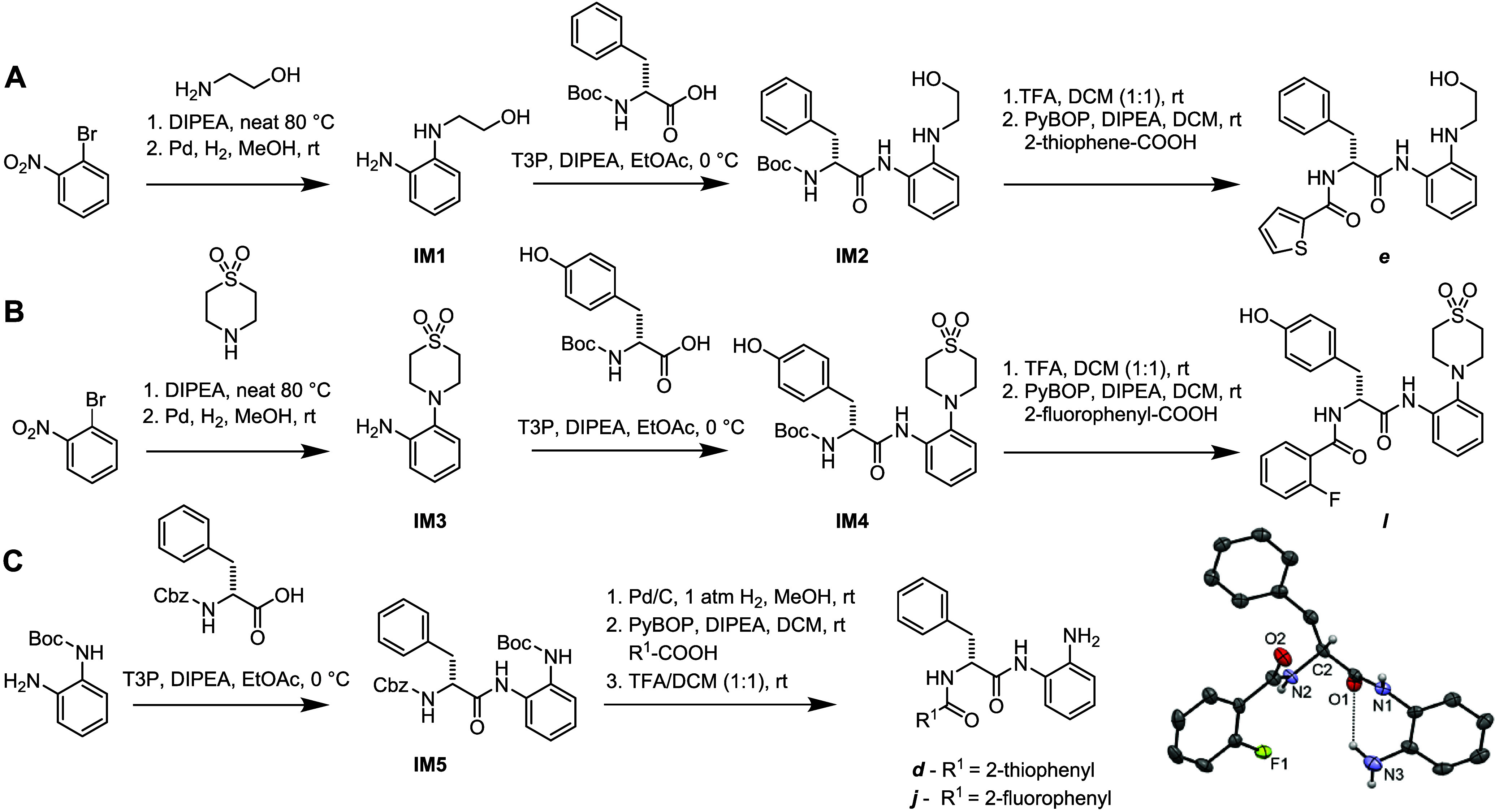
Overview of the synthetic routes leading to metabolites *d*, *e, j*, and *l*: (A) Synthesis of the metabolite *e* derived from MMV688845, (B) synthesis of the metabolite *l* derived from AAP-SO_2_, and (C) synthesis of the metabolite *d* and *j* derived from MMV688845/AAP-SO_2_. The inset shows the molecular structure of compound *j* determined by X-ray crystallography (minor positional disorder of the 2-fluorophenyl group and carbon-bound H atoms, except for the H atom at C2, omitted for clarity).

The synthesis of metabolite *l* is summarized in Figure 6B. The initial step was an aromatic nucleophilic substitution of *o*-bromo-2-nitrobenzene with thiomorpholine dioxide followed by the hydrogenation of an aromatic nitro group, which leads to the formation of the building block 4-(2-aminophenyl)­thiomorpholine-1,1-dioxide (**IM3**). **IM3** was then coupled with *N*-Boc-d-tyrosine to form **IM4** using T3P as the coupling reagent. Next, the Boc protecting group was removed by TFA, and the free amine reacts with 2-fluorophenyl carboxylic acid to form a second amide bond using PyBOP leading to metabolite *l*.

For the synthesis of metabolites corresponding to *d* and *j*, commercially available *N*-Boc-1,2-phenylenediamine was used. The formation of the amide bond with *N*-Cbz-(D)-phenylalanine was performed using T3P (**IM5**) as the coupling reagent.^32^ The subsequent step is deprotection of the amine: The precursors of metabolites *d* or *j* were protected with a Cbz-protecting group and cleavage was achieved through reduction with a palladium catalyst under a hydrogen atmosphere. After the free amine was obtained, it was coupled to 2-fluorophenyl carboxylic acid/2-thiophenyl carboxylic acid. For this synthetic step, PyBOP in DCM was used as the coupling reagent.^31^ For metabolites corresponding to *d* and *j*, the last step is a deprotection of the aromatic amine, which is carried out using TFA in DCM. The molecular structure of compound *j* was confirmed by X-ray crystallography (Figure 6C). As expected, both amide bonds adopt a *Z* conformation. In contrast to the parent AAP structures,^21^ the amine does not exhibit an N_amide_–H···O=C but an N_amine_–H···O=C intramolecular hydrogen bond and also shows a distinctly different intermolecular hydrogen bonding pattern (see Supporting Information).

### Antimycobacterial Characterization of Selected Metabolites

For this study, the metabolites *d*, *e*, *g*, and *l* were tested against *M. abscessus* subsp. *abscessus* ATCC 19977 and *Mycobacterium smegmatis* mc^2^ 155. The results are summarized in Table 4, which also shows the activity and stability of the parent compounds MMV688845 and AAP-SO_2_ ^28^ for comparison. Metabolites *d*, *e*, and *j* did not show any activity, but metabolite *l* exhibits antimycobacterial activity, with an MIC_90_ of 3.13 μM against both species. Thus, compound *l* can be identified as an active metabolite of the parent compound AAP-SO_2_, although a slight decrease in activity is observed through hydroxylation of the phenylalanine side chain. By comparison, AAP-SO_2_ exhibits an MIC_90_ of 0.78 μM against *M. abscessus* subsp. *abscessus* ATCC 19977 and *M. smegmatis* mc^2^ 155.^28^

**Table 4:**
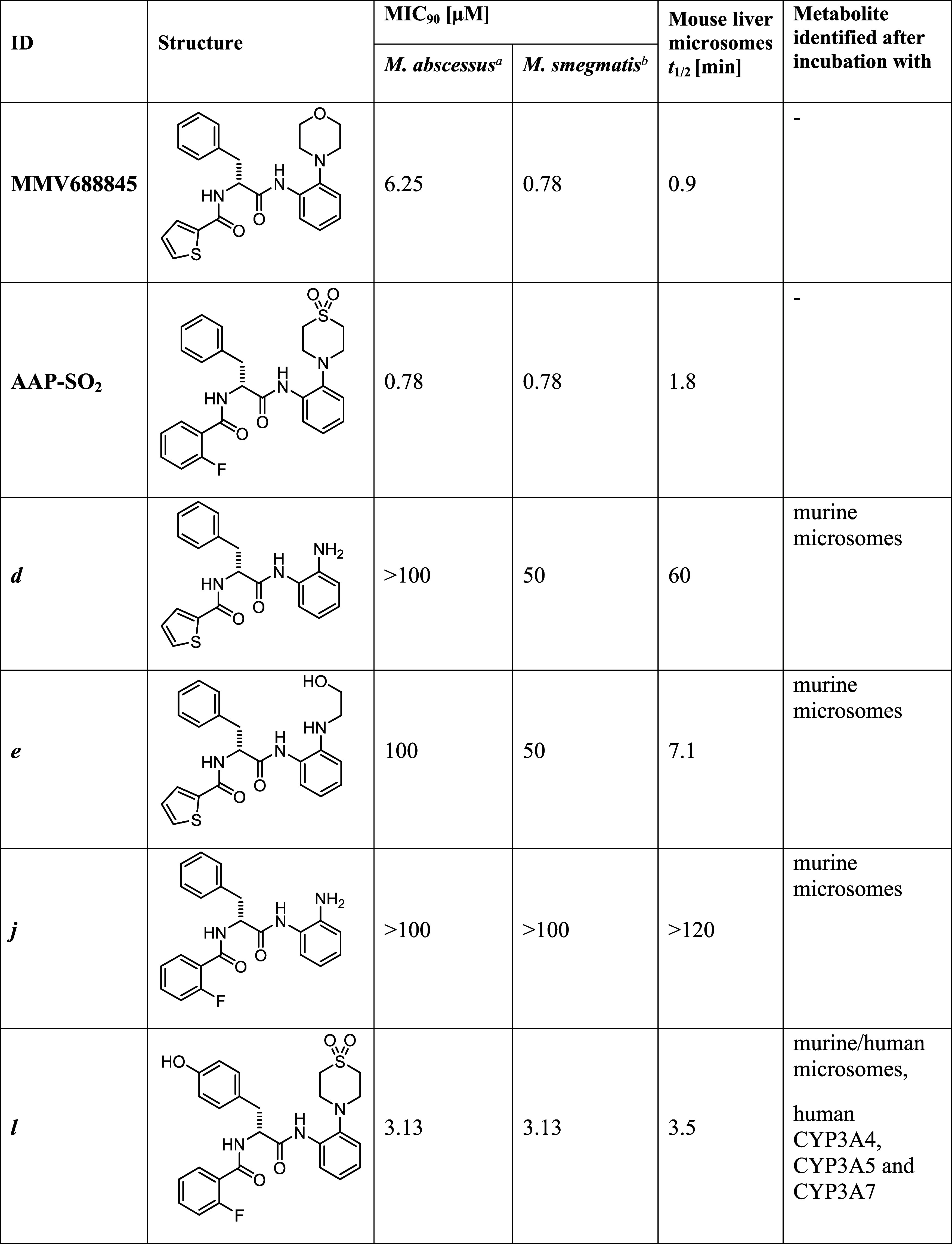
Antibacterial Activity against NTM and Microsomal Stability in Murine Liver Microsomes of AAP Metabolites

a *Mycobacterium abscessus* ssp. *abscessus* ATCC 19977.

b *Mycobacterium smegmatis* mc^2^ 155

In addition, the microsomal stability of the compounds *d*, *e*, and *g* was determined by incubation with mouse liver microsomes. The *t*_1/2_ was calculated, and the results show that the free aromatic amine *j* exhibits a *t*_1/2_ exceeding 120 min, while the thiophene-containing analogue *d* shows a *t*_1/2_ of 60 min. The intermediate metabolite *e* possesses significantly decreased stability with a *t*_1/2_ of only 6.6 min.

### The CYP Dependence of AAP Metabolism *In Vitro* and *In Vivo*

*In vitro* experiments were conducted to demonstrate that the metabolism of AAP-SO_2_ is primarily mediated by the oxidative metabolism of CYP enzymes. Microsomal stability was determined in the absence of the coenzyme NADPH, which was found to completely stop metabolic conversion (Figure 7A). This behavior indicates that oxidative CYP metabolism is crucial for the rapid degradation of AAP-SO_2_ in the microsomal suspensions. Furthermore, Figure 7B shows that doubling the microsomal concentration increases the degradation rate. Therefore, the rate of degradation is related to the enzyme concentration.

**Figure 7:**
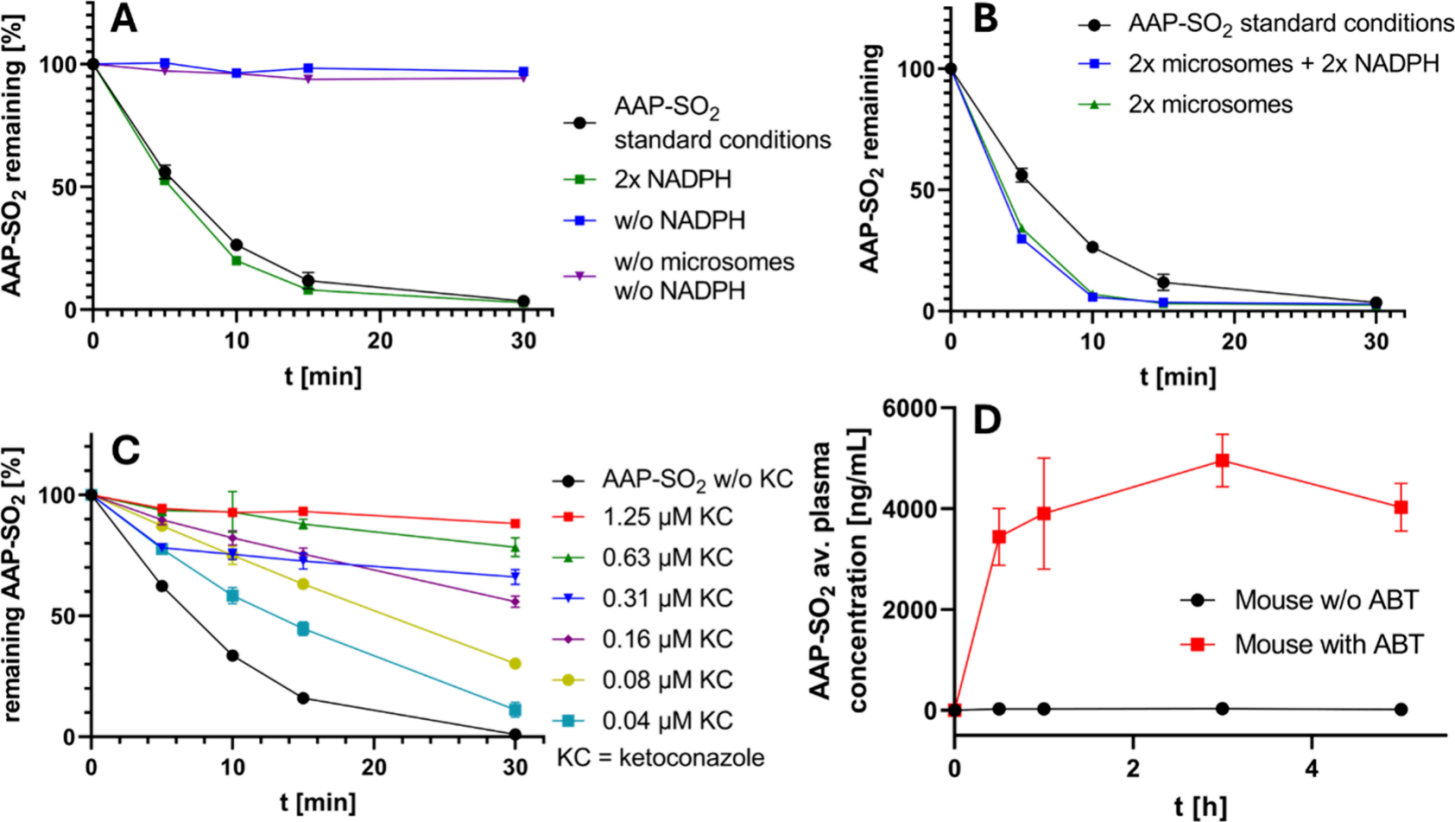
(A) Microsomal stability of AAP-SO_2_ depending on NADPH, (B) microsomal stability of AAP-SO_2_ depending on different microsome concentrations, (C) microsomal stability of AAP-SO_2_ depending on different concentrations of the CYP inhibitor ketoconazole (KC) concentrations, (D) plasma concentration–time profile of AAP-SO_2_ following a single oral dose of 25 mg kg^–1^ in CD-1 mice with and without administration of 1-aminobenzotriazole (ABT). Experiments were carried out in duplicate, and the results are represented as mean values with the standard deviations displayed as error bars.

The addition of the CYP inhibitor ketoconazole,^33–35^ as shown in Figure 7C, led to a dose-dependent slowdown in the degradation of AAP-SO_2_. Even low ketoconazole concentrations of 0.3 μM proved sufficient to inhibit the degradation of the active ingredient. Ketoconazole is a nonselective CYP inhibitor, but it is particularly known for interacting with CYP3A4. Therefore, the data suggest that inhibition of CYP3A4 in particular is capable of stopping the degradation of AAP-SO_2_.

Like MMV688845,^25^ AAP-SO_2_ does not show satisfactory bioavailability after oral administration in a mouse model, and the plasma concentration is significantly below the MIC of the drug candidate as shown in Figure 7D. However, the bioavailability could be significantly increased by adding the nonspecific CYP inhibitor 1-aminobenzotriazole (ABT),^36,37^ and plasma concentrations of up to 10 times the MIC could be achieved. These data show that hepatic metabolism in particular is an obstacle to the oral bioavailability of AAPs, while other factors such as solubility and permeability are adequate.

Improving microsomal stability will be a key factor in the further development of AAPs: Possible strategies for this include the use of electron-deficient (hetero)­aromatics that replace the oxidation-sensitive, electron-rich benzene ring.^38^ Heterocycles with one or more nitrogen atoms, such as pyridine or pyrimidine, are a suitable approach for this. Other options include an isosteric replacement of the entire (thio)­morpholinoaniline structure and the anilidic amide bond, whereby it must be taken into account that the NH group of the amide bond forms a hydrogen bond with the target enzyme RNA polymerase. A triazole or benzimidazole heterocycle can be used as a concrete starting point for replacing the amide bond while retaining the hydrogen bridge donor properties.^39^ However, we are not aware that such an approach to improving the metabolic stability of AAPs has yet been described in the literature. Compound D-AAP1, however, demonstrates that the *ortho*-phenylenediamine moiety is not essential for the antimycobacterial activity of AAPs and that the tertiary nitrogen atom can be replaced to improve the metabolic stability. In a study by Lang et al., it was shown that replacing the tertiary thiomorpholine nitrogen with a CH group led to a significant reduction in antimycobacterial activity.^28^ Furthermore, it was shown that the introduction of methyl or fluorine substituents at the *ortho*-phenylenediamine structure slightly improves microsomal stability but also reduces antimicrobial activity.^30^ The majority of published and active AAPs possess an *ortho*-phenylenediamine group. For further development of these compounds, it is therefore important to either replace this functional group with an isostere or reduce its susceptibility to oxidation.

## Conclusions

This study examines the metabolic conversion mechanisms of AAPs MMV688845 and AAP-SO_2_ in detail. For the class of AAPs as novel synthetic RNA polymerase inhibitors, we propose, for the first time, metabolites based on LC/MS-MS data. The *o*-phenylenediamine structure present in this class of substances was identified as a metabolic target that undergoes enzymatic oxidation. Mass loss of 2 Da, which is associated with this metabolic conversion, was detected in murine and human microsomes and represents the initial metabolic attack that contributes to the instability of this substance class. This metabolic attack is followed by the breakdown of the morpholine (MMV688845) or thiomorpholine dioxide (AAP-SO_2_) ring, for which various metabolites have been postulated on the basis of LC-MS/MS data. Different hydroxylations of AAP-SO_2_ were evident (metabolites *m*, *n*, and *o*), which could not be observed previously.

Using CYP inhibitors, it has been shown *in vitro* and *in vivo* that the oxidative metabolism of CYP enzymes is responsible for the low stability and bioavailability of AAPs. In addition, metabolites *d* and *j* could be identified beyond doubt by postsynthesis and also examined with regard to their stability and antimycobacterial efficacy. This revealed a loss of antimycobacterial activity and an increase in stability against microsomal degradation. It should be emphasized that *l* was described as an active metabolite with *p*-hydroxylation on the phenylalanine side chain.

Optimization of metabolic stability is essential for further development of this promising class of antimycobacterial substances. The data from metabolite identification summarized here now allow a rational approach to this goal for the first time. The *o*-phenylenediamine structure is a metabolic weak point of AAPs that should be modified or replaced. The study summarized in this article is intended to pave the way for the approaches discussed herein.

## Materials and Methods

### MIC Determination against *M. smegmatis* mc^2^ 155 and *M. abscessus* ATCC 19977 pTEC27

MIC values were determined by the broth microdilution method. 96-well flat bottom tissue culture plates (Sarstedt, 83.3924.500) were used. In the third well of each row, two times the desired highest concentration of each compound was added in 7H9 medium supplemented with 10% ADS (albumin, dextrose and saline) and 0.05% Tween 80. Each compound was diluted 2-fold in a nine-point serial dilution. The concentration of the starting inoculum was 5 × 10^5^ CFU mL^–1^. The starting inoculum was diluted from a preculture at the mid log phase (OD_600_ 0.3 to 0.8), and an OD_600_ of 0.1 was correlated to 1 × 10^8^ CFU mL^–1^. The plates were sealed with parafilm, placed in a container with moist tissue, and incubated for 3 days (*M. smegmatis*) or 4 days (*M. abscessus*) at 37 °C. Each plate had eight negative controls (1% dimethyl sulfoxide) and eight positive controls (100 μM amikacin). After incubation, the plates were monitored by OD measurement at 550 nm (BMG labtech Fluostar Optima). The *M. abscessus* plates were additionally evaluated by fluorescence measurement (λ_ex_ = 544 nm, λ_em_ = 590 nm).

Data analysis: Every assay plate contained eight wells with dimethyl sulfoxide (1%) as negative control, which corresponds to 100% bacterial growth and eight wells with amikacin (100 μM) as positive control in which 100% inhibition of bacterial growth was achieved. Controls were used to monitor the assay quality through determination of the *Z*’ score.^40^ The *Z*’ score was calculated as follows:

$$Z^{\prime }=1-\frac{3({\mathrm{SD}}_{\mathrm{amikacin}}+{\mathrm{SD}}_{\mathrm{DMSO}})}{M_{\mathrm{amikacin}}-M_{\mathrm{DMSO}}}$$

where SD is the standard deviation and *M* is the mean.

The percentage of growth inhibition was calculated by the equation:

$$\%\mathrm{growth}\;\mathrm{inhibition}=-100\%\times \frac{\mathrm{signal}(\mathrm{sample})-\mathrm{signal}(\mathrm{DMSO})}{\mathrm{signal}(\mathrm{DMSO})-\mathrm{signal}(\mathrm{amikacin})}$$

### LC-MS/MS Analysis of MMV688845 Metabolites in Murine Microsomal Suspensions

#### Materials – Reagents and Consumables

DMSO Chromasolv Plus, HPLC grade, ≥99.7% (Sigma-Aldrich, USA; Cat# 34869), acetonitrile Chromasolv, gradient grade, for HPLC, ≥ 99.9% (Sigma-Aldrich, USA; Cat# 34851), methanol, HiPerSolv, HPLC-gradient grade, ≥99.9% (VWR Chemicals, USA, Cat# 20864.320), KH_2_PO_4_ (Helicon, Cat# Am–O781–0.5), K_2_HPO_4_ (Helicon, Cat# Am–O705–0.5), MgCl_2_·6H_2_O (Santa Cruz Biotechnology, Inc., USA; Cat# sc-203126A), formic acid (Sigma-Aldrich, Cat# 94318), DMSO stock solutions of the tested compound(s) 20 mM, mouse liver microsomes: pooled, male BALB/c mice (XenoTech, M3000/lot #2010026), glucose-6-phosphate dehydrogenase from baker’s yeast, type XV (Sigma-Aldrich, USA; Cat #G6378), d-glucose-6-phosphate monosodium salt (Santa Cruz Biotechnology, Inc., USA; Lot# sc-210728), NADPH tetrasodium salt (Santa Cruz Biotechnology, Inc., USA; Lot# sc-202725), and verapamil hydrochloride (Sigma-Aldrich, USA; Cat# V4629) were used.

#### Equipment

A gradient HPLC system (Agilent Technologies, USA) with Waters Atlantis dC18 HPLC Column, 100 Å, 3.5 μm, 2.1 mm × 100 mm (Waters, cat #186001295), Agilent 6550 iFunnel Q-TOF with an AJS ion source (Agilent Technologies, USA), VWR Membrane Nitrogen Generators N2–04-L1466, nitrogen purity 99%+ (VWR, USA), Centrifuge 4–15C Qiagen (Sigma, Germany) with Sigma 4–15 Qiagen Rotor Nr.09100 were used.

#### Analytical System

Profiling of compound MMV688845 metabolites in mouse liver microsomes was performed using an Agilent 6550 iFunnel Q-TOF instrument from Agilent Technologies (USA) with a dual Agilent Jet Stream (AJS-ESI) ion source. The Agilent HPLC system comprised an Agilent 1200 G1312A binary pump, a 1200 G1367B HiP Sampler with a thermostat module G1330B, and a 1260 G1316A thermostated column compartment. The data acquisition and system control were performed by using MassHunter software from Agilent Technologies. Data postprocessing was performed using MassMetaSite 4.3 from Molecular Discovery (UK).

#### Incubation of MMV688845 with Mouse Liver Microsomes

Microsomal incubations were carried out in 96-well plates in 3 aliquots of 30 μL each (one for each time point). Liver microsomal incubation medium comprised phosphate buffer (100 mM, pH 7.4), MgCl_2_ (3 mM), NADPH (3 mM), glucose-6-phosphate (5.3 mM), and glucose-6-phosphate dehydrogenase (0.67 units/mL) with 0.42 mg of liver microsomal protein per mL. In the control reactions, the NADPH-cofactor system was substituted with phosphate buffer. Test compounds (2 μM, final solvent concentration 1.6%) were incubated with microsomes at 37 °C, shaking at 100 rpm. Three time points over 7 min were analyzed. The reactions were stopped by adding 5 volumes of methanol with internal standard to incubation aliquots followed by protein sedimentation by centrifuging at 6,000 rpm for 20 min. Each reaction was performed in duplicate. Supernatants were analyzed using the HPLC system coupled with a tandem mass spectrometer and high-resolution mass spectrometer.

### HPLC-HRMS Conditions

#### Chromatographic Conditions for Metabolite Detection

Columns: Waters Atlantis dC18 (2.1 × 100 mm, 3 μm), Mobile phase A: acetonitrile:water:formic acid = 5:95:0.1, Mobile phase B: acetonitrile:formic acid = 100:0.1, Linear gradient: 0 min 0% B, 2 min 0% B, 11 min 50% B, 18 min 70% B, 24 min 100% B, 27 min 100% B, 27.50 min 0% B, 33 min stop, a divert valve directed the flow to the detector from 2 to 27 min, elution rate: 500 μL/min, column temperature: 30 °C.

#### HRMS Conditions

Scan types: MS, AutoMS/MS; ion source: turbo spray; ionization mode: ESI; ion polarity: positive, MS range (*m*/*z*) 115–630, MS scan rate (spectra/s) 13.33, MS/MS range (*m*/*z*) 50–600, MS/MS scan rate (spectra/s) 5.00, isolation width MS/MS medium (∼4 amu); fixed collision energies 10.00, 20.00, 40.00; reference masses: 121.05087300; 622.02896000; instrument parameters: gas temp (°C): 290; Gas Flow (L/min): 13; Nebulizer (psig): 40; SheatGasTemp (°C): 375; SheatGasFlow (L/min): 11, AutoRecalibration: Enabled, with 1 average scan, 20 ppm detection window and Min Height 500 counts. Data storage was performed using Centroid mode.

### Metabolic Stability and Metabolite Identification of AAP-SO_2_ in Mouse Liver Microsomes

For the evaluation of phase I metabolic stability, the compound (1 μM) was incubated with 0.5 mg/mL pooled mouse liver microsomes (Xenotech, Kansas City, USA), 2 mM NADPH, 10 mM MgCl_2_ in 100 mM potassium phosphate buffer pH 7.4 at 37 °C for 120 min on a microplate shaker (Eppendorf, Hamburg, Germany). The metabolic stability of testosterone, verapamil, and ketoconazole was determined in parallel to confirm the enzymatic activity of mouse liver microsomes. The incubation was stopped after defined time points by precipitation of aliquots of enzymes with 2 volumes of cold internal standard solution (15 nM diphenhydramine in 10% methanol/acetonitrile). Samples were stored on ice until the end of the incubation and precipitated protein was removed by centrifugation (15 min, 4 °C, 4,000*g*). The remaining test compound at the different time points was analyzed by HPLC-MS/MS (Vanquish Flex coupled to a TSQ Altis Plus, Thermo Fisher, Dreieich, Germany) and used to determine half-life (*t*_1/2_).

For metabolite identification studies AAP-SO_2_ was incubated at 10 μM final concentration and samples were analyzed using HPLC-HRMS (Vanquish Flex coupled to a Q Exactive Focus, Thermo Fisher, Dreieich, Germany). LC conditions were as follows: column: Accucore Phenyl-Hexyl (2.6 μm, 100 × 2.1 mm; Thermo Fisher, Dreieich, Germany); temperature 40 °C; flow rate 0.500 mL/min; solvent A: water + 0.1% formic acid; solvent B: acetonitrile + 0.1% formic acid; gradient: 0–4.0 min 2–35% B, 4.0–7.0 min 35–98% B, 7.0–8.0 min 98% B, 8.0–10.0 min 2% B. MS analysis was performed using full scan mode (switching polarity, full MS resolution 35,000, scan range 200–2,000; data-dependent MS/MS (ddMS2) resolution 17,500, stepped collision energy with 17.5, 35, 52.5). Blank samples using DMSO were run in parallel for background subtraction. Sample processing for metabolite identification was performed using Compound Discoverer 3.2 (Thermo Fisher, Dreieich, Germany). Metabolites were identified based on mass shifts and feasibility of the metabolic reaction also in view of MS peak intensities over time.

### Selective Metabolism of AAP-SO_2_ and Metabolite Identification against Human CYP Enzymes

#### Chemicals

Formic acid (99%) and MgCl_2_ were from Honeywell Riedel-de Haen (Bucharest, Romania). Acetonitrile (ultragradient HPLC grade), methanol (HPLC gradient grade), and glycine were from Fisher J.T. Baker (Waltham, Massachusetts). Ethanol (≥99.5%, Etax Aa) was from Altia (Helsinki, Finland). Water was deionized by Milli-Q gradient A10. All chemicals were of the highest purity available from their commercial suppliers. Tris-HCl, MnCl_2_, MgCl_2_, isocitric acid, and isocitric acid dehydrogenase were purchased from Sigma-Aldrich (Steinheim, Germany), KCl was from J.T. Baker, and NADPH and NADP^+^ were from Roche Diagnostics (Mannheim, Germany). A 200 mL NADPH regenerating system contained 178.5 mg of NADP^+^ (nicotinamide adenine dinucleotide phosphate), 645 mg of isocitric acid, 340 mg of KCl, 240 mg of MgCl_2_, 0.32 mg of MnCl_2_, and 15 U of isocitric acid dehydrogenase.

#### Biological Material

Baculovirus-insect cell-expressed human CYP3A4, CYP3A5, and CYP3A7 were purchased from BD Biosciences Discovery Labware (Woburn, MA, USA) and used according to the manufacturer’s instructions.

Human liver microsomes were also purchased from BD Biosciences Discovery Labware (Woburn, MA, USA) and used according to the manufacturer’s instructions.

#### Incubation of AAP-SO_2_ with Recombinant Human CYPs and Human Liver Microsomes

AAP-SO_2_ was incubated with human CYP enzymes or human liver microsomes in 100 μL of 100 mM Tris-HCl buffer pH 7.4 containing 5 mM MgCl_2_, 10% NADPH regenerating system, 2 pmol recombinant human CYP enzymes or 2 μL human liver microsomes, and 10 μM AAP-SO_2_ as the substrate. Negative control mixtures were incubated (i) without the cofactor NADPH or (ii) without enzymes. Triplicate samples were incubated for 30 min at 37 °C, 300 μL of acetonitrile was added stopping the reaction, and samples were mixed and stored at −80 °C until analysis. 100 μL of 0, 0.1, 1, and 10 μM AAP-SO_2_ standards in 100 mM Tris-HCl pH 7.4 buffer were prepared as the samples. The samples were melted at RT and centrifuged at 10,000*g* for 15 min, and 200 μL of supernatant was taken into HPLC vials and then analyzed by an Orbitrap UHPLC-MS.

#### Analysis by UHPLC-MS

Metabolite analyses were carried out with a UHPLC-QTOF-MS System (Agilent Technologies 1290 LC, 6540 MS, Agilent Technologies, Santa Clara, CA, USA) using reversed-phase chromatography (RP) combined with positive mode electrospray ionization. The RP chromatography was performed on a Zorbax Eclipse XDB-C18 column (100 mm × 2.1 mm, 1.8 μm; Agilent Technologies). The column temperature was 50 °C, the flow rate wa 0.4 mL/min, and gradient elution was used with water (eluent A) and methanol (eluent B) containing 0.1% v/v of formic acid. The following gradient profile was employed: 0–7 min: 40 → 60% B; 7–8 min: 60 → 100% B; 8–10 min: 100% B; 10–10.1 min 100 → 40% B; 10–12 min: 40% B. The injection volume was 3 μL, and the sample tray was maintained at 10 °C. The MS ion source conditions were as follows: drying gas temperature of 325 °C, flow of 10 L/min, nebulizer pressure of 45 psi, capillary voltage of 3500 V. For data acquisition, the mass range was 20–1600 amu with a scan speed of 600 ms. In the targeted MS/MS analyses, the protonated metabolite ions were selected for fragmentation with a collision energy of 20 V. The quadrupole isolation width was 1.3 amu, and the scan speed was 500 ms. Continuous mass-axis calibration was performed by monitoring two reference ions from an infusion solution throughout the runs.

### CYP Dependence of AAP Metabolism *In Vitro –* Microsomal Stability of AAP-SO_2_ Depending on NADPH, Microsome Concentrations, and the CYP Inhibitor Ketoconazole Concentrations

#### Phosphate Buffer

A phosphate concentration of 100 mM was achieved by mixing 469.6 mL of ddH_2_0, 9.9 mL of 1 M KH_2_PO_4_ stock solution, and 20.5 mL of 2 M K_2_HPO_4_ stock solution. The stock solutions mentioned were also prepared with ddH_2_O. The buffer was adjusted to a pH value of 7.4 with the aid of 0.1 N KOH and a pH meter while stirring continuously. The final filtration was carried out in a previously sterilized safety cabinet using a disposable vacuum filtration system (polyethersulfone filter pore size = 0.22 μm).

#### Stock Solutions of the Test Compounds

Test compounds were dissolved at a concentration of 2 mM in 2 mL microscrew tubes with HPLC-grade DMSO used as the solvent.

#### Mouse Microsomal Suspensions

Mouse microsomal suspensions were purchased directly from Thermo Fisher (pooled mouse liver microsomes from CD-1 mice). For the assay, the microsomal suspension is divided into 35 μL aliquots (sufficient for one substance and its duplicate) and frozen in 1.5 mL reaction vessels at – 80 °C.

#### NADPH as a Cofactor

For an assay in reaction plates, 880 μL of a 5 mM solution of NADPH·4Na·H_2_O in phosphate buffer was required. The NADPH solution used was prepared from the solid compound stored at −20 °C shortly before use, as the cofactor is unstable in solution and this minimizes loss.

#### MgCl_2_ Solution

The solution was prepared by dissolving MgCl_2_·6*x*H_2_O in sterile phosphate buffer (100 mM, pH= 7.4). Similar to the phosphate buffer, this reagent was sterilized by filtration and stored in a sterile final container.

#### Termination Reagent with Internal Standard

A mixture of acetonitrile and methanol (ratio of 9:1, solvents of HPLC purity) was prepared. 2,3-Dihydroxynaphthalene was used as an internal standard, which was added to the prepared acetonitrile/methanol solution as a acetonitrile stock solution. Two separate termination reagents were prepared containing 4 μM and 400 nM internal standards. For 200 mL of 4 μM termination reagent, 80 μL of a 10 mM 2,3-dihydroxynaphthalene stock solution is added to 180 mL of acetonitrile and 20 mL of methanol. Similarly, for 200 mL of a 400 nM termination reagent, 800 μL of a 100 μM stock solution was added. To ensure that the reagent remains functional, it was essential to store it in a refrigerator at −20 °C and on ice during the assay.

Determination of microsomal stability in 96-well microtiter plates. The following reagents were mixed in a 1.5 mL microreaction vessel for one replicate of a stability determination (incubation mix): 471 μL of 100 mM phosphate buffer, 3 μL of DMSO stock solution of the test substance AAP-SO_2_, 15 μL of microsomal stock solution (20 mg/mL), 6 μL of 1 M MgCl_2_ solution. 250 μL of the incubation mix was pipetted into column 1 and the remaining 245 μL into column 2 of the microtiter plate. The filled and covered microtiter plate was pretempered for 10 min at 37 °C in the incubator. In the meantime, 5 mM NADPH solution was prepared by adding the appropriate amount of 100 mM phosphate buffer to the solid aliquot (store on ice). 110 μL of 5 mM NADPH solution was pipetted into column 3 followed by transferring 20 μL of the NADPH solution from column 3 into columns 4 to 8. Using a multichannel pipet, 100 μL of termination reagent with an internal standard was pipetted from a cooled reservoir into column 4. Then, 80 μL of incubation mix from columns 1/2 were pipetted into columns 4 to 8 and mixed immediately. The microtiter plate was placed back into the incubator with the lid on. At time points *t* = 5, 10, 15, and 30 min, the reaction was stopped by adding 100 μL termination reagent with internal standard. Once all time points had been terminated, the plate was centrifuged with the lid on at 4000 rpm for 15 min at 4 °C and analyzed by HPLC-UV.

#### Analytical HPLC-UV

The analytical HPLC system used consists of two LC-10 AD pumps, an SPD-M10A VP PDA detector, and a SIL-HAT autosampler from Shimadzu (Kyoto, Japan). The column used is a Poroshell 120 EC-C18 column (2.7 μm, 3.0 × 50 mm) from Agilent Technologies (Santa Clara, CA, USA). HPLC-grade water was used as mobile phase A and HPLC-grade acetonitrile as phase B, with 0.1% trifluoroacetic acid added to both eluents.

### *In Vivo* Bioavailability Analysis of AAP-SO_2_

Pharmacokinetics studies: CD-1 female mice (22–25 g) were used in oral pharmacokinetic studies (IACUC number AUP-25–02). All animal studies were approved by the Hackensack Meridian Health Institutional Animal Care and Use Committee and were aligned with applicable sections of the Guide for the Care and Use of Laboratory Animals (2011). Compounds for oral snapshot pharmacokinetic (PK) profiling were administered by oral gavage at 25 mg/kg in a vehicle consisting of 5% dimethylacetamide (DMA), 60% PEG300, and 35% D5W (5% dextrose in water). Mice pretreated with ABT were dosed ABT 1 h prior to test compound dosing at 30 mg kg^–1^ in a 0.5% carboxymethyl cellulose and 0.5% Tween 80 suspension in water. Aliquots of 30–40 μL of blood were taken by puncture of the lateral tail vein from each mouse (*n* = 2 per compound) at 30 min, 1, 3, and 5 h postdose. Blood was captured in CB300 blood collection tubes containing K_2_EDTA and stored on ice. Plasma was recovered after centrifugation and stored at −80 °C until analyzed by high pressure liquid chromatography coupled to tandem mass spectrometry (HPLC-MS/MS). Pharmacokinetic parameters were determined using noncompartmental pharmacokinetic analysis.

LC-MS/MS analytical methods: Neat 1 mg/mL DMSO stock of compounds were serially diluted in 50/50 acetonitrile (acetonitrile)/ Milli-Q water to create standard curves solutions. Standards were created by adding 10 μL of spiking solutions to 90 μL of drug-free plasma (CD-1 K_2_EDTA Mouse, Bioreclamation IVT). 10 μL of control, standard, or study sample were added to 100 μL of acetonitrile protein precipitation solvent containing 10 ng/mL of the internal standards verapamil (Sigma-Aldrich). Extracts were vortexed for 5 min and centrifuged at 4000 rpm for 5 min. 75 μL of supernatant were transferred for HPLC-MS/MS analysis and diluted with 75 μL of Milli-Q deionized water.

LC-MS/MS analysis was performed on a Sciex Applied Biosystems Qtrap 6500+ triple-quadrupole mass spectrometer coupled to a Shimadzu Nexera X2 UHPLC system to quantify each drug in the plasma. Chromatography was performed on an Agilent SB-C8 (2.1 × 30 mm; particle size, 3.5 μm) using a reversed-phase gradient. Milli-Q deionized water with 0.1% formic acid was used for the aqueous mobile phase and 0.1% formic acid in acetonitrile for the organic mobile phase. Multiple-reaction monitoring of parent/daughter transitions in electrospray positive-ionization mode was used to quantify the analytes. The following MRM transitions were used for AAP-SO_2_ (496.1/123.0) and verapamil (455.3/165.0). Sample analysis was accepted if the concentrations of the quality control samples were within 20% of the nominal concentration. Data processing was performed using Analyst software (version 1.6.2; Applied Biosystems Sciex).

## Supplementary Material









## Notes

CIF file of compounds (CIF)
CIF file of compounds (PDF)
Coordinate data of compounds (XYZ)
Structures and synthetic protocols of intermediates and final compounds, NMR spectra, HPLC-purity chromatograms, LC-MS/MS spectra for metabolite identification, and additional information on X-ray crystallography (the authors have cited additional references within the Supporting Information) (PDF)

## Notes

CCDC 2515366

## ABBREVIATIONS

ABT: 1-aminobenzotriazole
AAP: *N*α-aroyl-*N*-aryl-phenylalanine amides
DIPEA: *N*,*N*-diisopropylethylamine
MDR: multi drug resistant
NTM: nontuberculous mycobacteria
NTM-PD: NTM pulmonary disease
PyBOP: (benzotriazol-1-yloxy)­tripyrrolidinophosphonium hexafluorophosphate
T3P: propanephosphonic acid anhydride
TFA: trifluoroacetic acid
