# Supplementary material for: Cytochrome P450-Mediated Metabolism of Antimycobacterial Nα‑Aroyl‑N‑aryl-phenylalanine Amides
Source: ACS Infect Dis. 2026 Jun 2;12(6):1992–2003. doi: 10.1021/acsinfecdis.6c00100 (PMC13270519; doi:10.1021/acsinfecdis.6c00100)
Supplement: Supplementary file 4 [file id6c00100_si_004.pdf]

Supporting Information: Cytochrome P450-Mediated Metabolism of antimycobacterial *N*α-Aroyl-*N*-Aryl-Phenylalanine Amides

Johannes Doering [a], Erik Meerz [a], Rüdiger W. Seidel [a], Markus Lang [a], Andreas M. Kany [b,c], Seppo Auriola [d], Risto Olavi Juvonen [d], Hannu Raunio [d], Richard Goddard [e], Matthew D. Zimmerman [f], Véronique Dartois [f,g], Anna K. H. Hirsch [b,c,h], Thomas Dick [f,g,i], and Adrian Richter [a]*

[a] Institut für Pharmazie, Martin-Luther-Universität Halle-Wittenberg, Wolfgang-Langenbeck-Str. 4, 06120 Halle (Saale), Germany, [b] Helmholtz Institute for Pharmaceutical Research Saarland (HIPS) – Helmholtz Centre for Infection Research (HZI), Campus Building E8.1, 66123, Saarbrücken, Germany, [c] PharmaScienceHub, Campus Building A2.3, 66123, Saarbrücken, Germany, [d] School of Pharmacy, Faculty of Health Sciences, University of Eastern Finland, Box 1627, FI-70211, Kuopio, Finland [e] Max-Planck-Institut für Kohlenforschung, Kaiser-Wilhelm-Platz 1, 45470 Mülheim an der Ruhr, Germany, [f] Center for Discovery and Innovation, Hackensack Meridian Health, Nutley, New Jersey, USA, [g] Hackensack Meridian School of Medicine, Hackensack Meridian Health, Nutley, NJ 07110, [h] Saarland University, Department of Pharmacy, Campus Building E8.1, 66123, Saarbrücken, Germany, [i] Department of Microbiology and Immunology, Georgetown University, Washington, DC, USA

Corresponding Author

[*]Adrian Richter - Institut für Pharmazie, Martin-Luther-Universität Halle-Wittenberg, Wolfgang-Langenbeck-Str. 4, 06120 Halle (Saale), Germany; orcid.org/0000-0002-0062-7896; Email: adrian.richter@pharmazie.uni-halle.de

Table of Contents

1. Chemistry experimental procedures and analytical data S1-S10

2. X-ray crystallography S11-S15

3. $^{1}H/^{13}C/^{19}F$-NMR Spectra S16-S10

4. HRMS mass spectra and HPLC chromatograms of the *in vitro* test compounds S21-S26

5. LC-MS/MS Data for metabolite identification S27-S49

References S50

1. Chemistry experimental procedures and analytical data

General Methods Chemistry:

All purchased starting materials were used as received without further purification. Solvents used for synthesis or purification were distilled and stored over 4 Å-molecular sieves. Reactions were checked with thin-layer-chromatography (Merck TLC silica gel 60 F_{254} plates) and analysed under 254 nm UV light. Flash chromatography was performed with a puriFlash® 430 instrument (Interchim, Montluçon, France). Columns were packed with the help of a Büchi Cartridger C-670 in either 8 g (v = 10 mL min^{-1}), 45 g (v = 30 mL min^{-1}) or 90 g (v = 40 mL min^{-1}) cartridges with 40-63 µm normal phase silica gel produced by Carl Roth. The column loading was performed by using the dry load method. Preparative HPLC was also employed for purification using a Polaris 5 C18-A column (5 µm, 21.2 mm × 250 mm; Agilent Technologies, Santa Clara, USA) on a Shimadzu system (Kyoto, Japan) equipped with two LC-20 AD pumps, an SPD-M10A VP PDA detector and a SIL-HAT autosampler. A water/acetonitrile mixture was used as the mobile phase.

All test compounds were purified to an HPLC purity of >95 %. Analytical HPLC for purity verification was performed using the same autosampler and detector with a Poroshell 120 EC-C18 column (2.7 µm, 3.0 × 50 mm; Agilent Technologies, Santa Clara, USA) and two LC-10 AD pumps.

Compound characterization was achieved using an expression CMS mass spectrometer (Advion Inc., Ithaca, NY, USA) with ASAP (atmospheric solids analysis probe) sampling and a Plate Express TLC extractor. High-resolution mass spectra were obtained on an LTQ Orbitrap XL mass spectrometer (Thermo Fisher Scientific Inc., Waltham, MA, USA). NMR were recorded using an Agilent Technologies 400 MHz VNMRS spectrometer, an Agilent Technologies 500 MHz DD2 spectrometer and an Agilent 600 MHz VNMRS spectrometer at 25 °C. Chemical shifts are reported relative to the residual solvent signals ($CDCl_3$: δ_H = 7.26 ppm; δ_C = 77.36 ppm). The operating frequency for ^1H NMR was 400, 500 or 600 MHz and for ^{13}C NMR 100 MHz. The spectra were analyzed with MNova 11.0 and processed with baseline and automatic phase correction.

Synthesis of 2-((2-Nitrophenyl)amino)ethan-1-ol (IM1-NO$_2$)

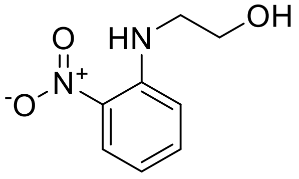

1-Bromo-2-nitrobenzene (1665 mg, 8.24 mmol, 1.0 eq.) was dissolved in ethanolamine (1495 µl, 24.72 mmol, 3.0 Äq.) and stirred for 72 h at 80 °C. After completion the mixture was diluted with dichloromethane and purified with flash chromatography using a 25-75 % gradient with a mobile phase containing ethyl acetate and heptane. The final yield was 1043 mg (70 %).

^{1}H NMR (402 MHz, $CDCl_3$) δ 8.15 (dd, J = 8.6, 1.6 Hz, 1H), 7.47 – 7.38 (m, 1H), 6.90 (dd, J = 8.7, 1.3 Hz, 1H), 6.71 – 6.62 (m, 1H), 4.59 (d, 2H), 3.97 – 3.89 (m, 2H), 3.50 (t, J = 5.4 Hz, 2H).

Mass: Calculated m/z for $C_8H_{10}N_2O_3^+$ $[M+H]^+$ =183.07; found APCI: 182.9

Synthesis of 2-((2-Aminophenyl)amino)ethan-1-ol (IM1)

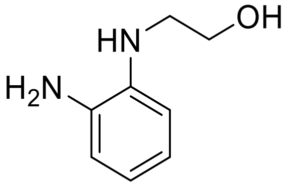

IM1-NO$_2$ (1043 mg, 5.73 mmol, 1.0 Äq.) was dissolved in methanol and $Pd(OH)_2$ on carbon (104 mg, 10 wt%) was added. The mixture was stirred at room temperature under a hydrogen gas atmosphere for 3 h. The catalyst was filtered off, and the filtrate was evaporated under reduced pressure without further purification. The yield was 846 mg (97 %).

Mass: Calculated m/z for $C_8H_{12}N_2O^+$ $[M+H]^+$ =153.09; found APCI: 152.9

Synthesis of *tert*-butyl (R)-(1-((2-((2-hydroxyethyl)amino)phenyl)amino)-1-oxo-3-phenylpropan-2-yl)carbamate (IM2)

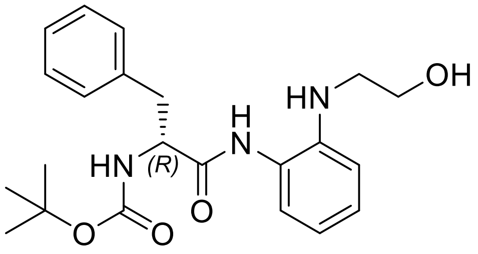

IM1 (212 mg, 1.39 mmol, 1.0 eq.) was combined with *N*-(*tert*-butoxycarbonyl)-D-phenylalanine (738 mg, 2.78 mmol, 2.0 eq.) and DIPEA (726 µl, 4.17 mmol, 3.0 eq.) in ethyl acetate. The solution was stirred at -20 °C for 10 min, then T3P (882 µl, 2.78 mmol, 2.0 eq.) was added. The reaction mixture was stirred at 0 °C for 2 h, followed by purification via flash chromatography using a 25-75 % ethyl acetate/heptane gradient. The reaction yielded 472 mg (85 %).

^{1}H NMR (502 MHz, $CDCl_3$) δ 7.99 (s, 1H), 7.45 – 6.96 (m, 8H), 6.89 – 6.77 (m, 2H), 5.30 (s, 1H), 4.47 (q, *J* = 7.2 Hz, 1H), 3.80 – 3.71 (m, 2H), 3.31 – 3.21 (m, 2H), 3.21 – 3.07 (m, 2H), 1.41 (s, 9H).

Mass: Calculated m/z for $C_{22}H_{29}N_3O_4^+$ $[M+H]^+$ =400.22; found APCI: 399.8

Synthesis of (R)-2-Amino-*N*-(2-((2-hydroxyethyl)amino)phenyl)-3-phenylpropanamide (deprotected IM2)

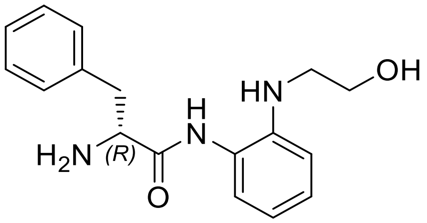

A solution of **IM2** (455 mg, 1.14 mmol, 1.0 eq.) in dichloromethane (2 ml) was treated with TFA (2 ml) and stirred at room temperature for 1 h. The mixture was then diluted with dichloromethane and washed with $NaHCO_3$ solution. The organic layer was dried over $MgSO_4$ and evaporated in vacuo, yielding 128 mg (38 %).

Mass: Calculated m/z for $C_{17}H_{21}N_3O_2^+$ $[M+H]^+$ =300.16; found APCI: 299.8

Synthesis of (R)-N-(1-((2-((2-hydroxyethyl)amino)phenyl)amino)-1-oxo-3-phenylpropan-2-yl)thiophene-2-carboxamide (*e*)

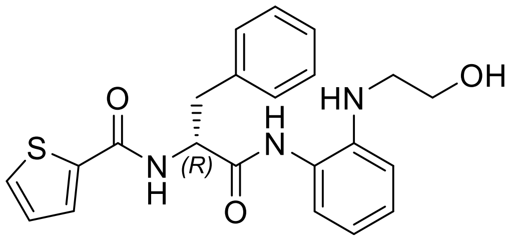

Deprotected IM2 (128 mg, 0.43 mmol, 1.0 eq.), 2-thiophenecarboxylic acid (60 mg, 0.47 mmol, 1.1 eq.), DIPEA (224 µl, 1.29 mmol, 3.0 eq.) and PyBOP (245 mg, 0.47 mmol, 1.1 eq.) were combined in dichloromethane and the mixture was stirred at room temperature for 24 h. The crude product was purified by flash chromatography with a mixture of ethyl acetate and heptane (20-40 %), followed by preparative HPLC with a gradient of 40-70 % in acetonitrile and water. Lyophilization afforded 75 mg (42 %) of the final product.

^{1}H NMR (502 MHz, CD_3CN) δ 8.05 (s, 1H), 7.66 – 7.59 (m, 2H), 7.50 (s, 1H), 7.37 – 7.24 (m, 5H), 7.14 – 7.06 (m, 2H), 6.85 (d, *J* = 7.6 Hz, 1H), 6.68 (dd, *J* = 8.2, 2.7 Hz, 1H), 6.64 – 6.57 (m, 1H), 4.71 – 4.62 (m, 1H), 3.70 – 3.63 (m, 2H), 3.58 – 3.31 (m, 1H), 3.33 – 3.24 (m, 1H), 3.24 – 3.19 (m, 1H), 3.19 – 3.10 (m, 2H), 2.38 (s, 1H).

^{13}C NMR (126 MHz, CD_3CN) δ 171.49, 163.61, 145.21, 139.34, 138.32, 132.26, 130.40 (d, *J* = 3.5 Hz), 129.96, 129.47 (d, *J* = 3.0 Hz), 129.01, 128.90, 128.10, 127.79, 123.55, 117.14, 112.44, 61.19 (d, *J* = 3.6 Hz), 57.61, 46.95, 37.85.

Mass: calculated m/z for $C_{22}H_{23}N_3O_3S^+$ $[M+H]^+$: 410.1533; found ESI: 410.1533

4-(2-aminophenyl)thiomorpholine 1,1-dioxide (IM3)

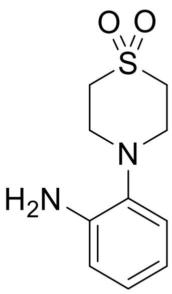

4-(2-nitrophenyl)thiomorpholine 1,1-dioxide (prepared according to Lang et al., 2023[1]) (1041 mg, 4.06 mmol, 1.0 Äq.) was dissolved in methanol and $Pd(OH)_2$ on carbon (201 mg, 10 wt%)

was added. The mixture was stirred at room temperature under a hydrogen gas atmosphere for 1 h. The catalyst was filtered off, and the filtrate was evaporated under reduced pressure without further purification. The yield was 410 mg (45 %).

Mass: Calculated m/z for $C_{10}H_{15}N_2O_2S^+$ $[M+H]^+$ =227.09; found APCI: 226.8

Synthesis of tert-butyl (*R*)-(1-((2-(1,1-dioxidothiomorpholino)phenyl)amino)-3-(4-hydroxyphenyl)-1-oxopropan-2-yl)carbamate (IM4)

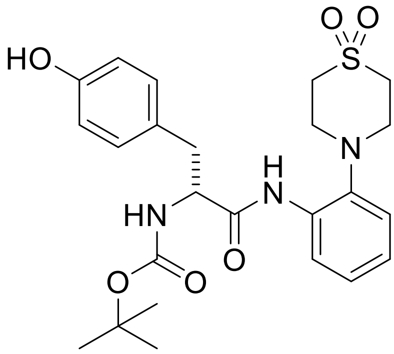

IM3 (140 mg, 0.62 mmol, 1.0 eq.) was combined with *N*-(*tert*-butoxycarbonyl)-D-tyrosine (250 mg, 0.89 mmol, 1.5 eq.) and DIPEA (326 µl, 1.87 mmol, 3.0 eq.) in ethyl acetate. The solution was stirred at -20 °C for 10 min, then T3P (411 µl, 0.69 mmol, 1.1 eq.) was added. The reaction mixture was stirred at 0 °C for 12 h, followed by purification via flash chromatography using a 30-80 % ethyl acetate/heptane gradient. The reaction yielded 130 mg (43 %).

^1H NMR (600 MHz, $CDCl_3$) δ 8.69 (s, 1H), 8.34 (s, 1H), 7.24 – 7.20 (m, 1H), 7.16 (dd, *J* = 8.0, 1.5 Hz, 1H), 7.13 – 7.06 (m, 3H), 6.75 (d, *J* = 8.5 Hz, 2H), 5.24 (s, 1H), 4.39 (s, 1H), 3.39 – 3.01 (m, 10H), 1.42 (s, 9H).

Mass: Calculated m/z for $C_{24}H_{32}N_3O_6S^+$ $[M+H]^+$ =490.20; found APCI: 490.3

Synthesis of (*R*)-*N*-(1-((2-(1,1-dioxidothiomorpholino)phenyl)amino)-3-(4-hydroxyphenyl)-1-oxopropan-2-yl)-2-fluorobenzamide (compound *I*)

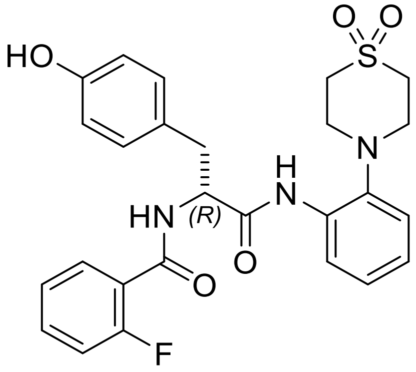

Intermediate **IM4** (91 mg, 0.23 mmol, 1.0 eq.), 2-fluorobenzoic acid (36 mg, 0.26 mmol, 1.1 eq.), DIPEA (121 µL, 0.7 mmol, 3.0 eq.) and PyBOP (133 mg, 0.26 mmol, 1.1 eq.) were combined in dichloromethane and the mixture was stirred at room temperature for 12 h. The crude product was purified by flash chromatography with a mixture of ethyl acetate and heptane (30-70 %), followed by preparative HPLC with a gradient of 40-70 % in acetonitrile and water. Lyophilization afforded 12 mg (11 %) of the final product ***I***. HPLC purity 98.6 %, t_R = 3.2 min

^{1}H NMR (600 MHz, $CDCl_3$) δ 8.99 (s, 1H), 8.39 – 8.33 (m, 1H), 8.06 (td, *J* = 7.9, 1.8 Hz, 1H), 7.57 – 7.49 (m, 1H), 7.44 (dd, *J* = 13.9, 7.1 Hz, 1H), 7.29 (t, *J* = 7.6 Hz, 1H), 7.23 (t, *J* = 7.8 Hz, 1H), 7.21 – 7.13 (m, 4H), 7.09 (t, *J* = 7.7 Hz, 1H), 6.78 (d, *J* = 8.5 Hz, 2H), 4.98 (q, *J* = 6.5 Hz, 1H), 3.37 – 3.20 (m, 6H), 3.20 – 3.10 (m, 4H).

^{13}C NMR (151 MHz, $CDCl_3$) δ 169.25, 163.90 (d, *J* = 3.1 Hz), 160.99 (d, *J* = 248.5 Hz), 155.64, 139.92, 134.47 (d, *J* = 9.6 Hz), 132.71, 131.92 (d, *J* = 1.5 Hz), 130.80, 127.99, 127.18, 125.36 (d, *J* = 3.1 Hz), 124.82, 121.48, 120.91, 119.80 (d, *J* = 10.8 Hz), 116.63 (d, *J* = 24.5 Hz), 115.99, 56.92, 51.93, 51.54, 37.38.

^{19}F NMR (564 MHz, $CDCl_3$) δ -110.45 – -115.73 (m).

Mass: calculated m/z for $C_{26}H_{27}FN_3O_5S^+$ $[M+H]^+$: 512.1650; found ESI: 512.1654, calculated m/z for $C_{26}H_{26}FN_3O_5SNa^+$ $[M+Na]^+$: 534.1469; found ESI: 534.1472

Synthesis of benzyl (R)-(1-((2-((*tert*-butoxycarbonyl)amino)phenyl)amino)-1-oxo-3-phenylpropan-2-yl)carbamate (IM5)

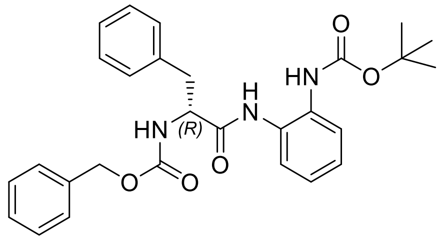

Tert-butyl (2-aminophenyl)carbamate (369 mg, 1.77 mmol, 1.0 eq.) and *N*-((benzyloxy)carbonyl)-*D*-phenylalanine (644 mg, 2.15 mmol, 1.2 eq.) were dissolved in ethyl acetate. After adding DIPEA (925 µl, 5.32 mmol, 3.0 eq.), the solution was stirred at -20 °C for 10 minutes. The coupling reagent T3P was added as a 50% solution in ethyl acetate (3165 µl, 5.32 mmol, 3.0 eq.) and the mixture was stirred at 0°C for 24h. The solution was concentrated under reduced pressure and purified by flash chromatography, using a 30-60 % gradient containing ethyl acetate and heptane. Due to insufficient separation, further purification was performed by preparative HPLC using a gradient of 40-95% acetonitrile/water. The collected fractions were lyophilized, yielding 391 mg (45%).

^{1}H NMR (402 MHz, $CDCl_3$) δ 8.12 (s, 1H), 7.45 (d, *J* = 8.1 Hz, 1H), 7.35 – 7.22 (m, 9H), 7.22 – 7.03 (m, 4H), 6.68 (s, 1H), 5.37 (s, 1H), 5.16 – 5.03 (m, 2H), 4.59 – 4.45 (m, 1H), 3.23 – 3.08 (m, 2H), 1.47 (s, 9H).

Mass: Calculated m/z for $C_{28}H_{31}N_3O_5^+$ $[M+H]^+$ = 490.23; found APCI: 490.0

Synthesis of *tert*-butyl (R)-(2-(2-amino-3-phenylpropanamido)phenyl)carbamate (deprotected IM3)

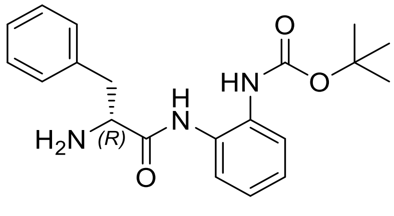

To a solution of **IM5** (391 mg) in methanol, $Pd(OH)_2$ on carbon (31 mg, 8 wt%) was added. The reaction mixture was stirred at room temperature under a hydrogen atmosphere for 3 h. Then the solution was filtered and the filtrate concentrated under reduced pressure without further purification. The final yield was 296 mg (98%).

Mass: Calculated m/z for $C_{20}H_{25}N_3O_3^+$ $[M+H]^+$ =356.19; found APCI: 356.0

Synthesis of *tert*-butyl (R)-(2-(3-phenyl-2-(thiophene-2-carboxamido)propanamido)phenyl) carbamate (Boc-*d*)

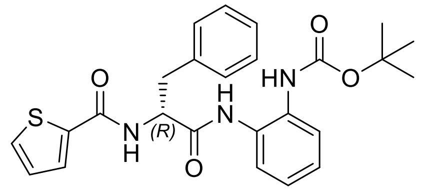

The crude **deprotected IM3** (148 mg, 0.42 mmol, 1.0 eq.), 2-thiophenecarboxylic acid (59 mg, 0.46 mmol, 1.1 eq.) and PyBOP (239 mg, 0.46 mmol, 1.1 eq.) were dissolved in dichloromethane and DIPEA (219 µl, 1.26 mmol, 3.0 eq.) was added. The reaction mixture was stirred for 24 h at room temperature. Afterwards the solution was directly concentrated under reduced pressure and purified using flash chromatography with a solvent mixture of ethyl acetate and heptane (gradient 10-90%). The yield was 163 mg (83 %).

^1H NMR (402 MHz, $CDCl_3$) δ 8.42 (s, 1H), 7.54 – 7.50 (m, 1H), 7.47 – 7.44 (m, 2H), 7.32 – 7.20 (m, 6H), 7.17 – 7.11 (m, 1H), 7.07 – 6.99 (m, 2H), 6.97 – 6.89 (m, 1H), 6.81 (s, 1H), 4.97 (q, *J* = 7.0 Hz, 1H), 3.35 – 3.20 (m, 2H), 1.46 (s, 9H).

^{13}C NMR (101 MHz, $CDCl_3$) δ 169.84, 161.99, 153.84, 137.86, 136.04, 131.01, 130.75, 129.38, 128.84, 128.79, 128.34, 127.73, 127.27, 126.58, 125.04, 124.84, 123.91, 80.90, 77.31, 76.99, 76.68, 55.59, 38.46, 28.27.

Mass: calculated m/z for $C_{25}H_{27}N_3O_4SNa^+$ $[M+Na]^+$: 488.1615; found ESI: 488.1621

Synthesis of (R)-*N*-(1-((2-aminophenyl)amino)-1-oxo-3-phenylpropane-2-yl)thiophene-2-carboxamide (*d*)

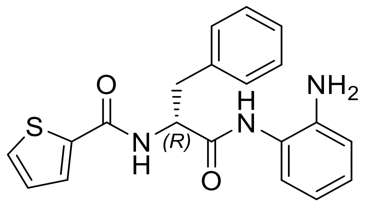

Boc-*d* was dissolved in dichloromethane (2 ml), followed by addition of TFA (2 ml). The reaction mixture was stirred at room temperature for 1 h. After dilution with additional dichloromethane and washing with $NaHCO_3$ solution, the organic layer was concentrated

under reduced pressure. The crude product was purified by preparative HPLC using a 40-80% gradient mixture of acetonitrile and water. The collected fractions were lyophilized, yielding 51 mg (80 %) of the final product.

^{1}H NMR (402 MHz, CD_3CN) δ 8.03 (s, 1H), 7.65 – 7.57 (m, 2H), 7.39 – 7.21 (m, 6H), 7.12 (dd, *J* = 4.9, 3.8 Hz, 1H), 7.05 – 6.93 (m, 2H), 6.73 (dd, *J* = 8.0, 1.4 Hz, 1H), 6.64 (td, *J* = 7.6, 1.4 Hz, 1H), 4.84 – 4.74 (m, 1H), 4.11 (s, 2H), 3.32 (dd, *J* = 13.8, 6.5 Hz, 1H), 3.23 – 3.13 (m, 1H).

^{13}C NMR (126 MHz, CD_3CN) δ 171.09, 162.93, 138.55, 132.00, 130.43, 129.56, 129.47, 128.95, 128.33, 127.75, 127.67, 123.62, 118.29, 117.08, 56.92, 38.28.

Mass: calculated m/z for $C_{20}H_{19}N_3O_2S^+$ $[M+H]^+$: 366.1271; found ESI: 366.1272

Synthesis of *tert*-Butyl (R)-(2-(2-(2-fluorobenzamido)-3-phenylpropaneamido)phenyl)carbamate (Boc-*j*)

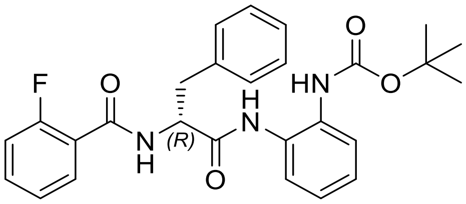

Deprotected IM3 (148 mg, 0.42 mmol, 1.0 eq.), 2-fluorobenzoic acid (64 mg, 0.46 mmol, 1.1 eq.), PyBOP (239 mg, 0.46 mmol, 1.1 eq.) were dissolved in dichloromethane, followed by addition of DIPEA (219 µl, 1.26 mmol, 3.0 eq.). The mixture was stirred at room temperature for 24 h and then purified directly by flash chromatography with a solvent mixture containing ethyl acetate and heptane (10-90%). The product was obtained in 174 mg (87 %) yield.

^{1}H NMR (402 MHz, $CDCl_3$) δ 8.43 (s, 1H), 7.99 (td, *J* = 7.9, 1.9 Hz, 1H), 7.55 (d, *J* = 1.4 Hz, 1H), 7.50 – 7.36 (m, 2H), 7.32 – 6.99 (m, 10H), 6.92 (s, 1H), 5.05 – 4.95 (m, 1H), 3.27 (d, *J* = 6.9 Hz, 2H), 1.46 (s, 9H).

^{13}C NMR (101 MHz, $CDCl_3$) δ 169.66, 163.57 (d, *J* = 3.1 Hz), 161.97, 159.51, 153.82, 136.04, 133.80 (d, *J* = 9.4 Hz), 131.91 (d, *J* = 1.9 Hz), 131.24, 129.35, 128.81, 128.29, 127.24, 126.54, 125.07, 124.75 (d, *J* = 3.2 Hz), 124.70, 123.84, 120.20, 116.14 (d, *J* = 24.5 Hz), 80.68, 55.98, 38.27, 28.27.

^{19}F NMR (378 MHz, $CDCl_3$) δ -112.44 – -112.66 (m).

Mass: calculated m/z for $C_{27}H_{28}FN_3O_4^+$ $[M+H]^+$: 478.2137; found ESI: 478.2139

Synthesis of (R)-*N*-(1-((2-Aminophenyl)amino)-1-oxo-3-phenylpropane-2-yl)-2-fluorobenzamide (*j*)

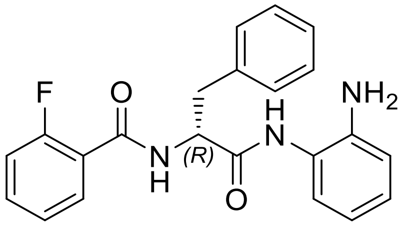

Deprotection was performed by dissolving **Boc-*j*** (87 mg, 0.18 mmol, 1.0 eq.) in dichloromethane (2 ml) and adding TFA (2 ml). The resulting mixture was stirred at room temperature for 1 h, then diluted with dichloromethane and washed with $NaHCO_3$ solution. The organic phase was evaporated directly on silica gel and flash chromatography was performed using a 25-75 % gradient of ethyl acetate and heptane. Final purification was achieved by preparative HPLC with a 40- 80 % gradient of acetonitrile and water. The fractions were lyophilized, yielding 50 mg (73 %).

^{1}H NMR (402 MHz, DMSO D6) δ 9.33 (s, 1H), 8.48 – 8.41 (m, 1H), 7.58 – 7.45 (m, 2H), 7.36 – 7.11 (m, 7H), 7.02 (dd, *J* = 7.9, 1.5 Hz, 1H), 6.89 (td, *J* = 7.6, 1.5 Hz, 1H), 6.68 (dd, *J* = 8.0, 1.4 Hz, 1H), 6.50 (td, *J* = 7.5, 1.4 Hz, 1H), 4.89 – 4.80 (m, 1H), 4.78 (s, 2H), 3.15 (dd, *J* = 13.7, 5.5 Hz, 1H), 3.05 (dd, *J* = 13.7, 8.9 Hz, 1H).

^{13}C NMR (101 MHz, DMSO D6) δ 169.69, 163.62, 160.55, 158.08, 142.53, 137.58, 132.68 (d, *J* = 8.3 Hz), 130.22 (d, *J* = 2.8 Hz), 129.32, 128.10, 126.42, 126.33, 125.88, 124.37 (d, *J* = 3.5 Hz), 122.48, 116.22, 115.98 (d, *J* = 5.0 Hz), 115.53, 55.25, 37.42.

^{19}F NMR (378 MHz, CD_3CN) δ -114.31 – -114.45 (m)

Mass: calculated m/z for $C_{22}H_{20}FN_3O_2^+$ $[M+H]^+$: 378.1612; found ESI: 378.1620

2. X-ray crystallography

Needle-shaped crystals of compound ***j*** suitable for crystal structure analysis were obtained from a solution in ethyl acetate by layering with heptane, followed by slow evaporation of the solvents at room temperature (Figure S1). A crystal was prepared in perfluoropolyether PFO-XR75 and mounted on a MiTeGen cryo loop with the aid of an optical microscope. The diffraction data were measured on a Bruker AXS Mach3 four-circle diffractometer with an IµS microfocus X-ray source and an APEX II CCD detector. The diffractometer was controlled using the APEX3 software (Bruker AXS, Karlsruhe, Germany) and the data reduction was performed using SAINT (Bruker AXS, Karlsruhe, Germany). An absorption correction using the Gaussian integration method was applied with SADABS-2016/2 (Bruker AXS, Karlsruhe, Germany).

The crystal structure was solved using SHELXT[2] and refined using SHELXL2019/3[3]. Anisotropic displacement parameters were introduced for all non-hydrogen atoms. Positional disorder of the fluorobenzene ring was described using a split model with appropriate geometric restraints (see Figure S2 and supplementary crystallographic data). The six-membered ring of the minor disorder component was treated as an ideal hexagon and the split atoms were refined with equivalent anisotropic atomic displacement parameters. Refinement of the ratio of occupancy factors using a free variable resulted in 0.921(4):0.079(4). Hydrogen atoms bonded to carbon were placed in geometrically calculated positions and refined using a riding model. Amide and amine hydrogen atoms were located by difference Fourier synthesis and refined with the following geometric restraints: N_{amide}–H = 0.88(2) Å and N_{amine}–H = 0.91(2) Å. U_{iso}(H) = 1.2 U_{eq}(C, N) was set for all hydrogen atoms. In the absence of significant anomalous scattering, the absolute structure could not be determined from the diffraction data [Flack *x* parameter calculated using Parsons' quotient method using 815 quotients [(*I*+)-(*I*-)]/[(*I*+)+(*I*-)] = 0.4(7)][4]. The *R* configuration of the molecule was assigned based on the known configuration of the starting material in the synthesis. Crystal data and refinement details for compound ***j*** are listed in Table S1. Structure pictures were created using Mercury[5] or Diamond (Crystal Impact GbR, Bonn, Germany). CCDC 2515366 contains the supplementary crystallographic data for this paper. These data can be obtained free of charge from The Cambridge Crystallographic Data Centre via www.ccdc.cam.ac.uk/structures.

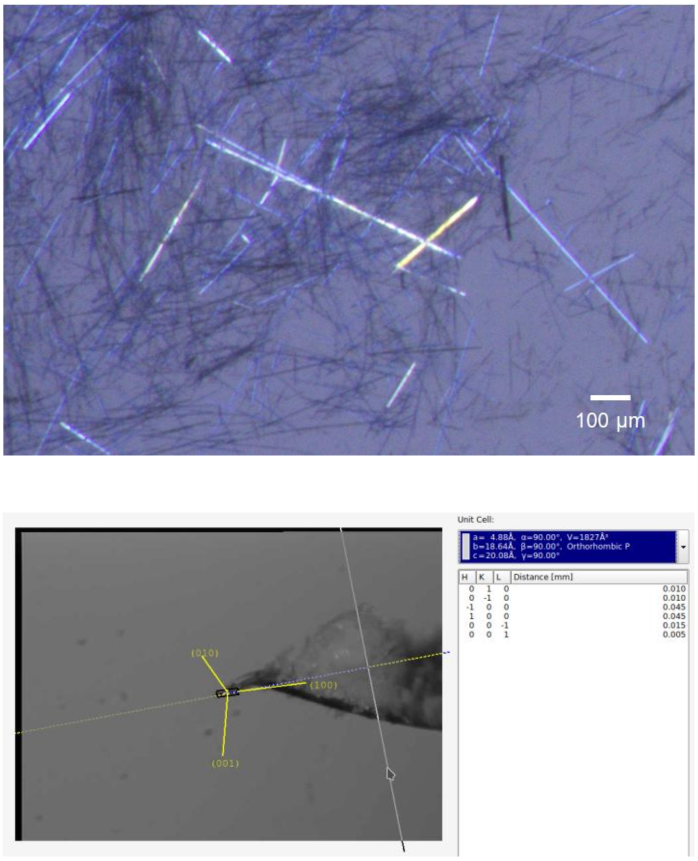

Figure S1. Microscope image of needle-shaped crystals of compound ***j*** crystallized from ethyl acetate/heptane (top) and a view of the crystal used for structure determination (bottom).

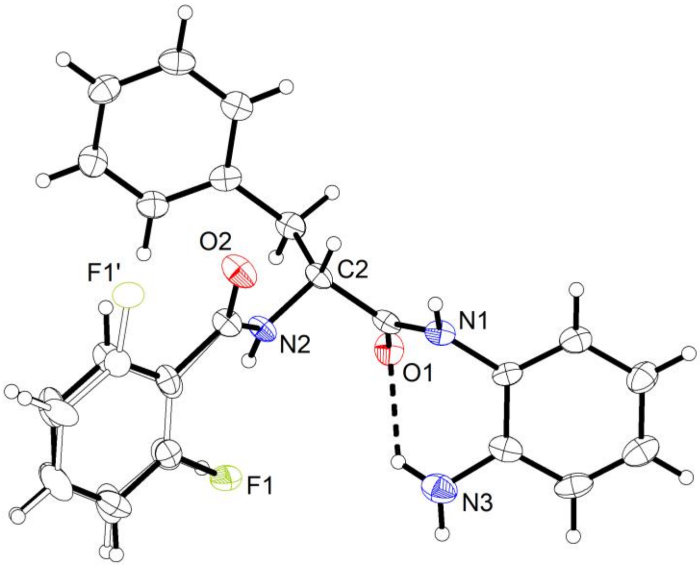

Figure S2. Displacement ellipsoid plot (50% probability) of compound ***j*** in the crystal. The minor disorder component of the fluorophenyl group is shown by empty ellipsoids. Hydrogen atoms are

represented by small spheres of arbitrary radius. The dashed line illustrates an intramolecular hydrogen bond.

Table S1. Crystal data and refinement details for compound ***j***.

Empirical formula	$C_{22}H_{20}FN_3O_2$
M_r	377.41
T (K)	100(2)
λ (Å)	0.71073
Crystal system, space group	Orthorhombic, $P2_12_12_1$
a (Å)	4.8828(5)
b (Å)	18.634(2)
c (Å)	20.078(2)
V (Å^3)	1826.8(3)
Z, Calculated density (g cm^{-3})	4, 1.372
μ (mm^{-1})	0.097
F(000)	792
Crystal size (mm)	0.091 × 0.032 × 0.021
θ range for data collection (°)	1.491 to 30.655
Reflections collected / unique	59678 / 5585 [R_{int} = 0.2020]
Completeness to θ = 25.242° (%)	100.0
Data / restraints / parameters	5585 / 13 / 276
Goodness-of-fit on F^2	0.988
$R1$ [$I > 2\sigma(I)$]	0.0599
$wR2$ (all data)	0.1305
$\Delta\rho_{max}$, $\Delta\rho_{min}$	0.334, –0.374

Figure S3a illustrates the hydrogen bonding pattern in the crystal structure of compound ***j***, and Table S2 lists the corresponding geometric parameters. The molecules form strands extending parallel to the *a* axis via two N–H···O hydrogen bonds between the amide groups of adjacent molecules [graph set descriptor: $R_2^2(12)$]. BFDH morphology prediction (Figure S3b) suggests that this corresponds to the needle axis of the crystal and this was confirmed by experiment (*cf.* Figure S1). The amino group forms an intramolecular N–H···O hydrogen bond to an amide carbonyl oxygen atom [graph set descriptor: S(7)] and an intermolecular N–H···N hydrogen bond to the amino group of a neighbouring N–H···O

hydrogen-bonded strand, symmetry-related by a 2_1 screw axis parallel to the *a* axis direction. Comparing the molecular structure in the crystal with that of the minimum energy structure of the free molecule (Figure S4), suggests that the intermolecular hydrogen bonding pattern has a bearing on the molecular conformation. In addition to the intramolecular N_{amine}–H···O hydrogen bond also observed in the crystal, the structure of the free molecule exhibits an intramolecular N_{amide}–H···O hydrogen bond.

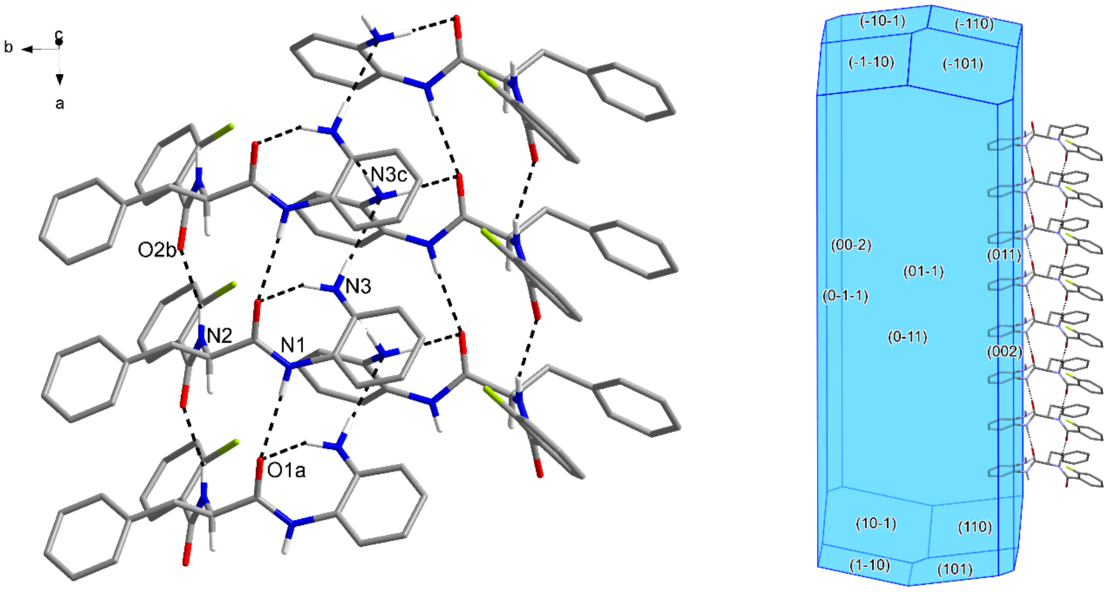

(a) **(b)**

Figure S3. (a) Section of the crystal structure of compound ***j***, viewed approximately along the *c* axis direction. Symmetry codes: (a) x+1, y, z; (b) x–1, y, z; (c) x–1/2, –y+1/2, –z+1. (b) BFDH morphology prediction for compound ***j*** (calculated with Mercury[5]). Disorder and carbon-bound hydrogen atoms are omitted for clarity.

Table S2. Hydrogen bond geometry for compound ***j*** (Å, °).

D*–H···*A	***d*(*D*–H)**	***d*(H···*A*)**	***d*(D···A)**	**<(*D*H*A*)**
N1–H1···O1a	0.87(2)	2.11(2)	2.977(3)	173(3)
N2–H2A···O2b	0.84(2)	2.03(3)	2.820(3)	156(3)
N3–H3A···N3c	0.93(2)	2.27(2)	3.190(5)	170(3)
N3–H3B···O1	0.94(2)	2.23(3)	2.952(4)	133(3)

Symmetry codes: (a) x+1, y, z; (b) x–1, y, z; (c) x–1/2, –y+1/2, –z+1.

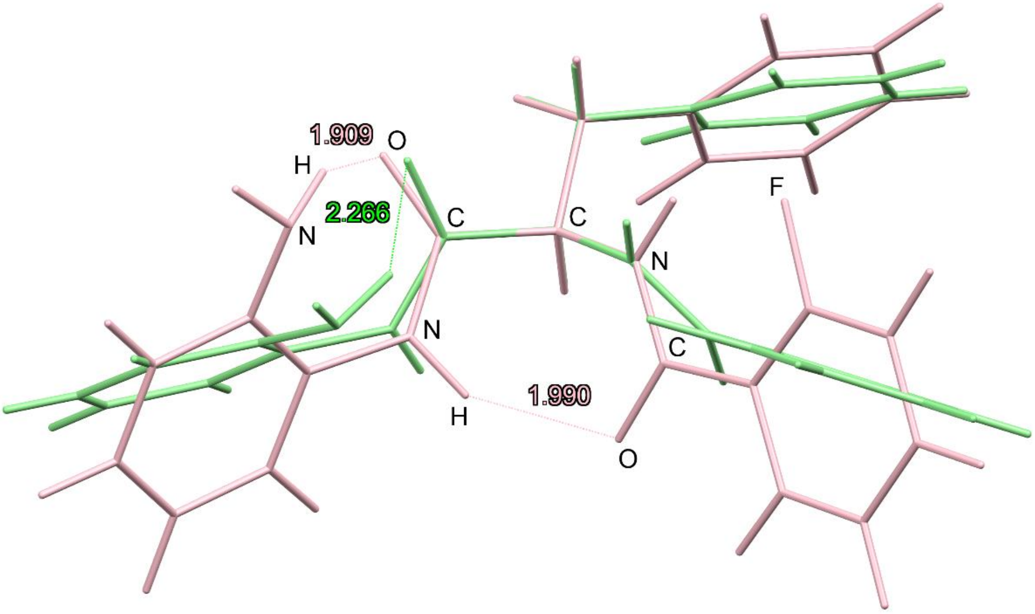

Figure S4 Structure overlay plot of the molecular structure of compound ***j*** in the crystal (light green) and the minimum energy structure of the free molecule (magenta), as calculated with ORCA version 6.1[6]; (B3LYP/G / def2-TZVPP). The dotted lines illustrate the intramolecular N_{amine}–H···O and N_{amide}–H···O hydrogen bonds.

3. $^{1}H/^{13}C/^{19}F$-NMR Spectra

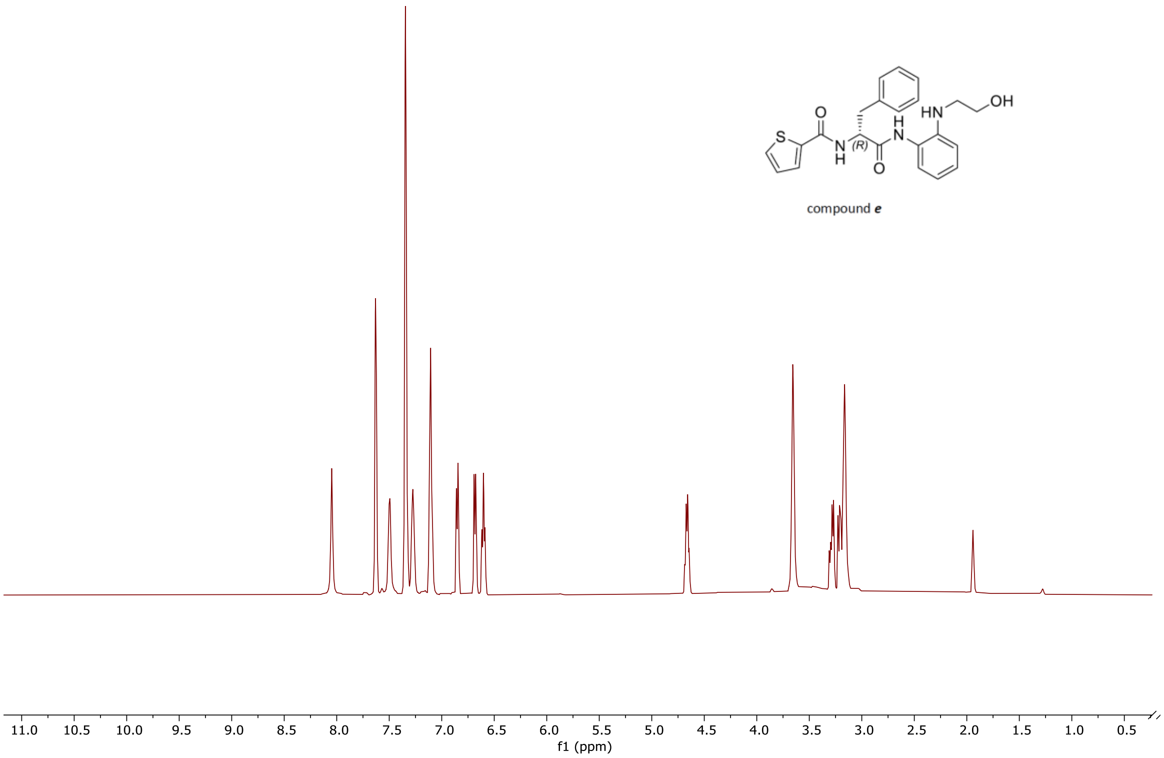

Figure S5. ^{1}H NMR spectrum (502 MHz, CD_3CN) of compound ***e***

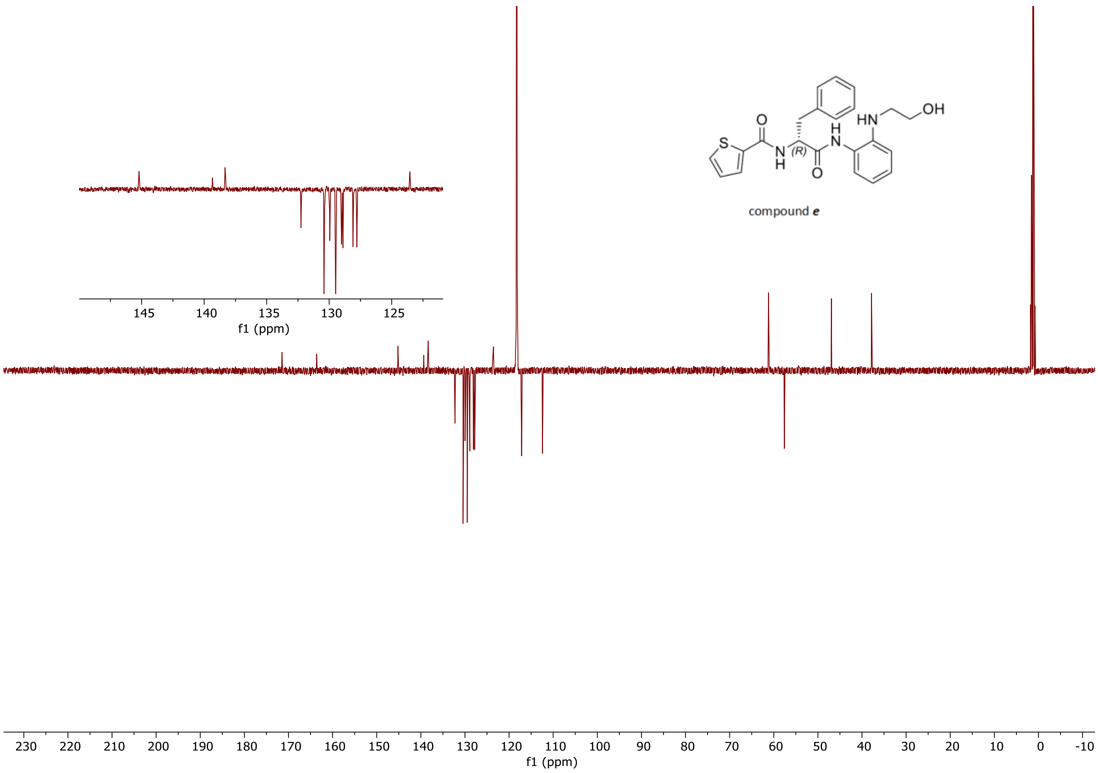

Figure S6. ^{13}C NMR spectrum (126 MHz, CD_3CN) of compound ***e***

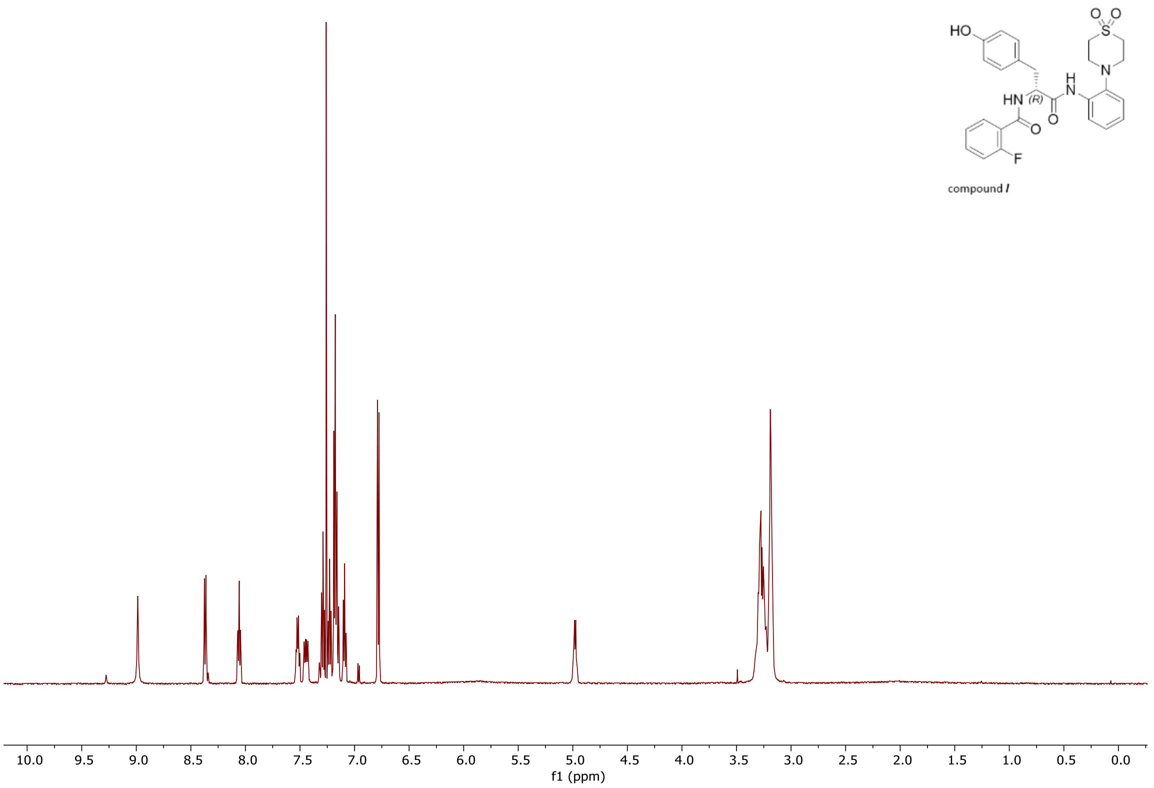

Figure S7. ^{1}H NMR spectrum (600 MHz, $CDCl_3$) of compound ***I***.

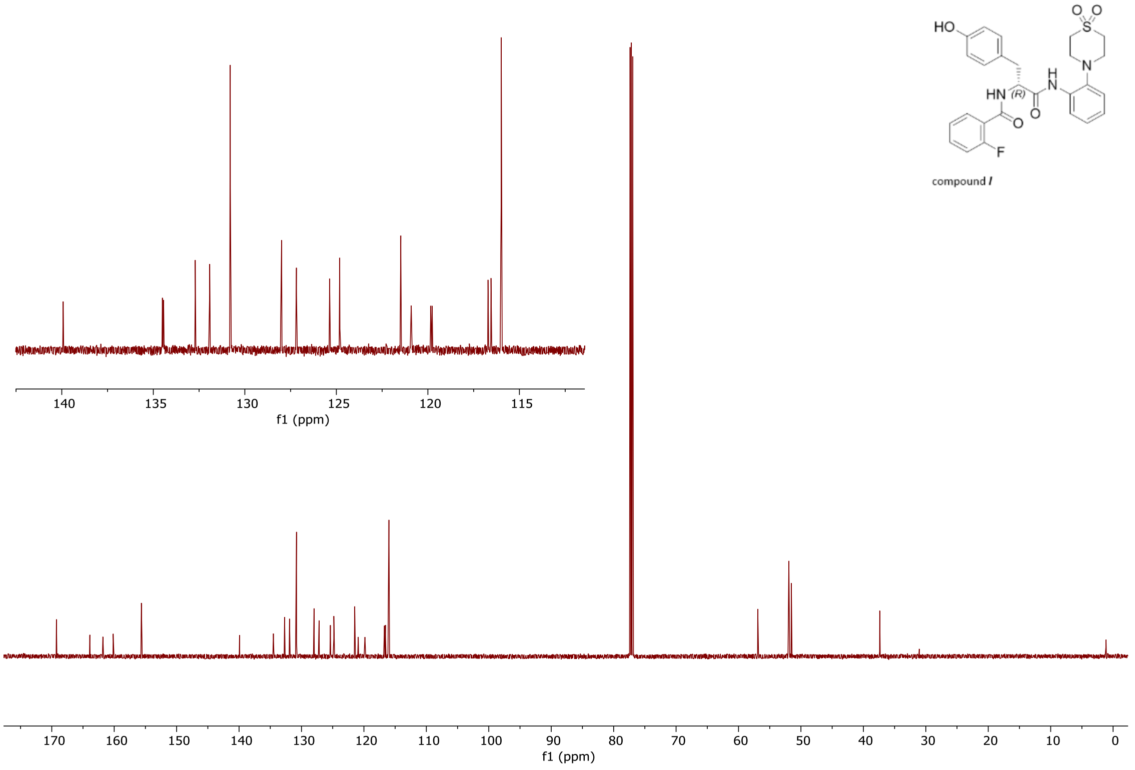

Figure S8. ^{13}C NMR spectrum (151 MHz, $CDCl_3$) of compound ***I***.

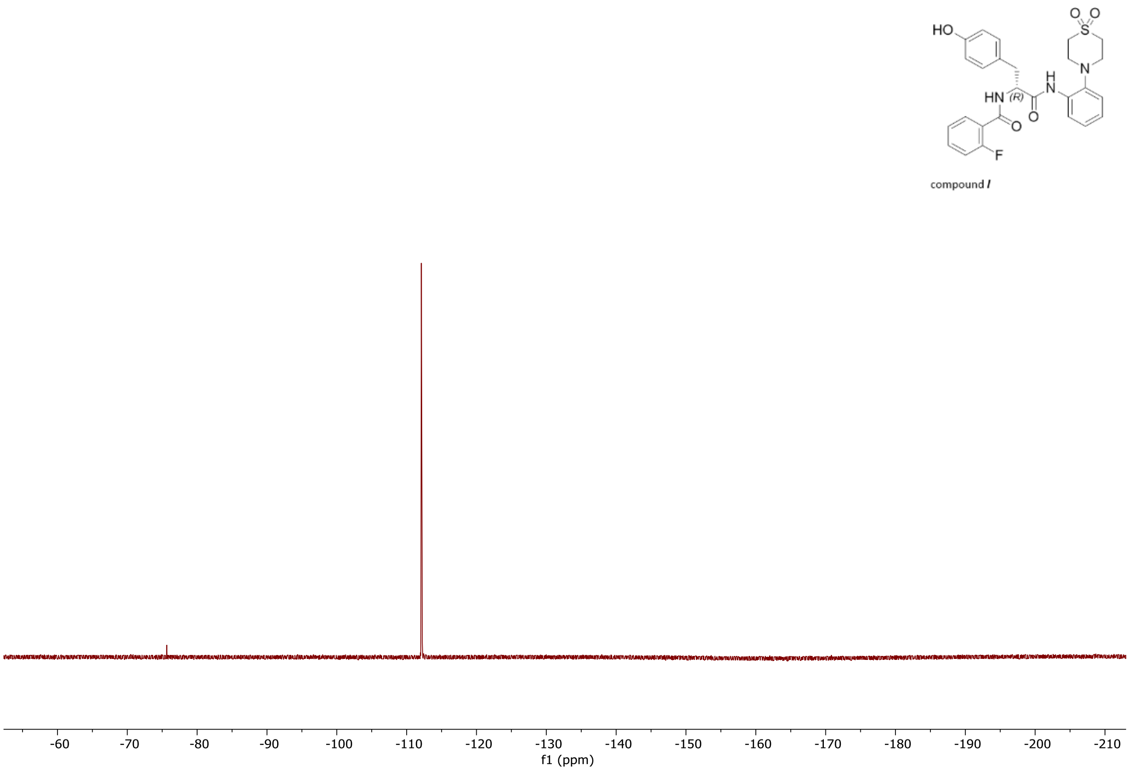

Figure S9. ^{19}F NMR spectrum (564 MHz, $CDCl_3$) of compound ***l***.

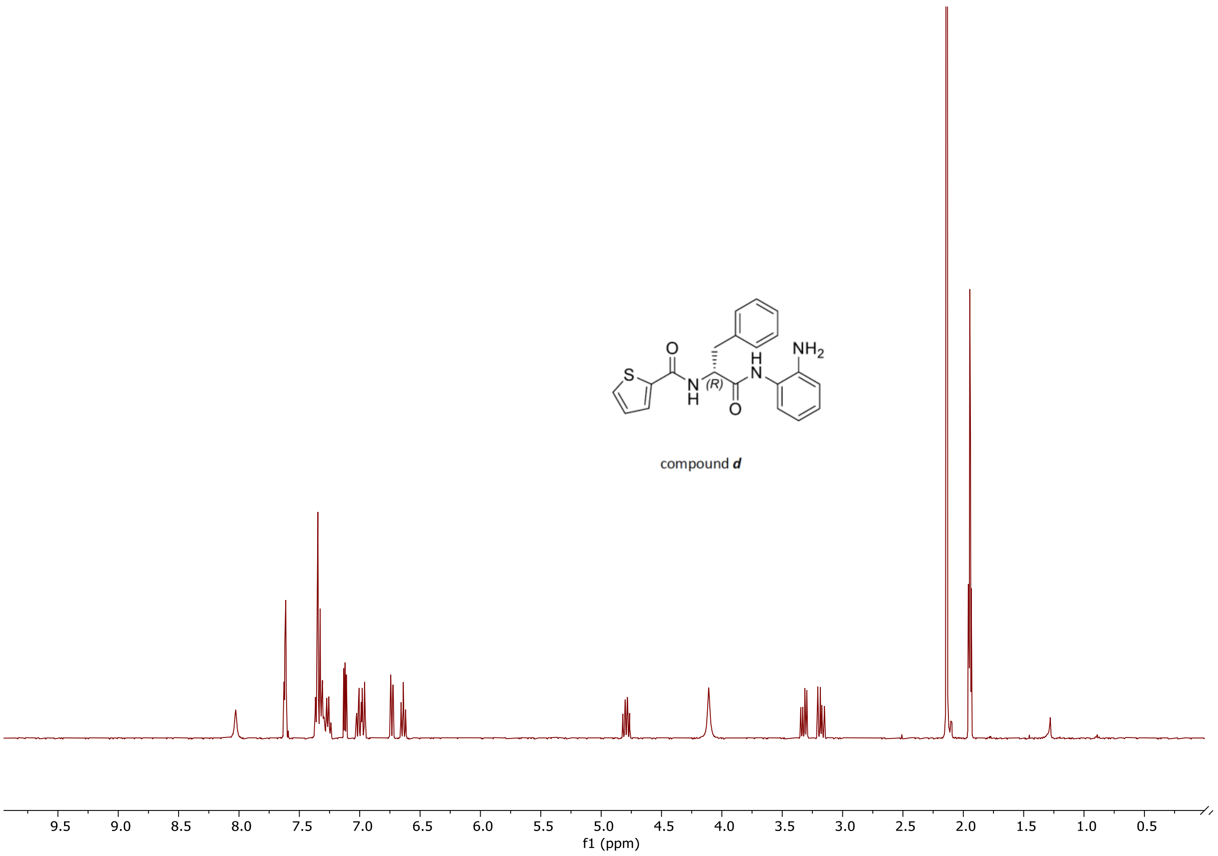

Figure S10. ^{1}H NMR spectrum (402 MHz, CD_3CN) of compound ***d***

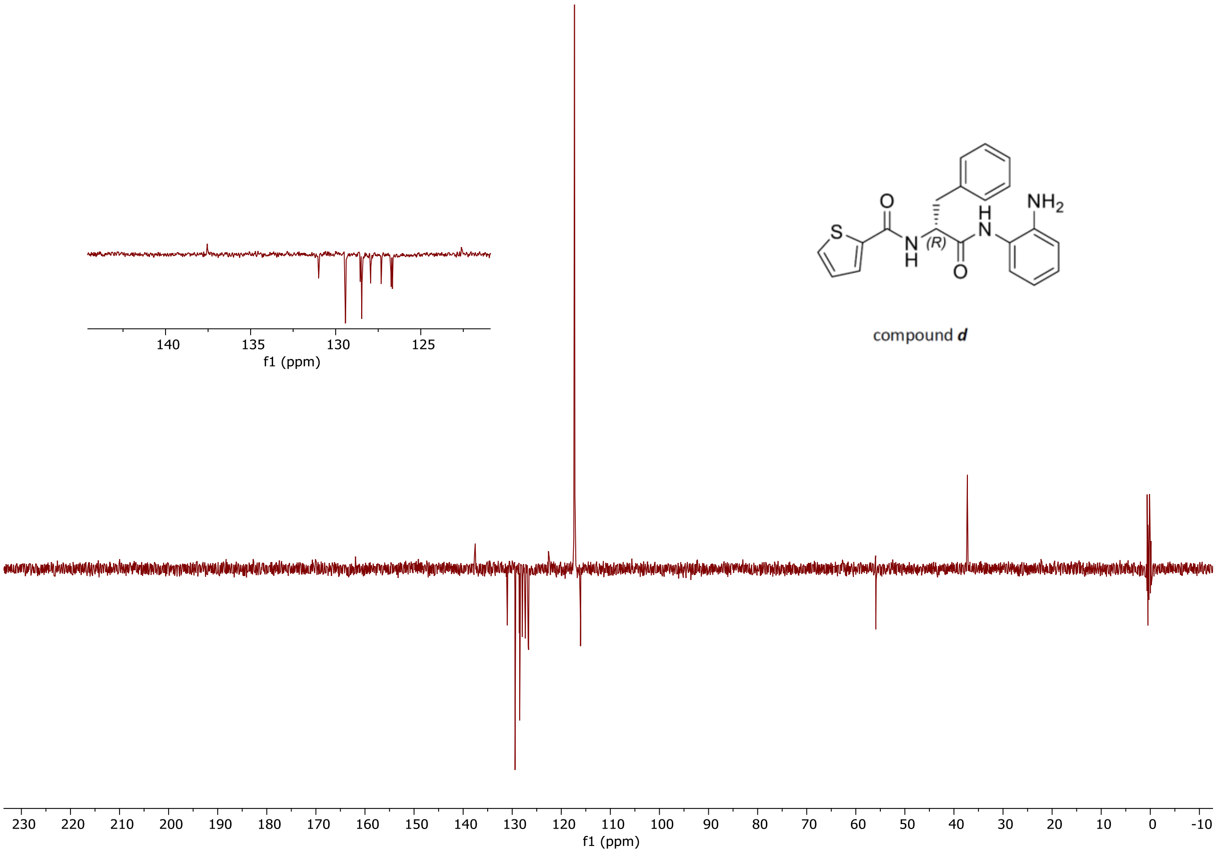

Figure S11. ^{13}C NMR spectrum (126 MHz, CD_3CN) of compound ***d***

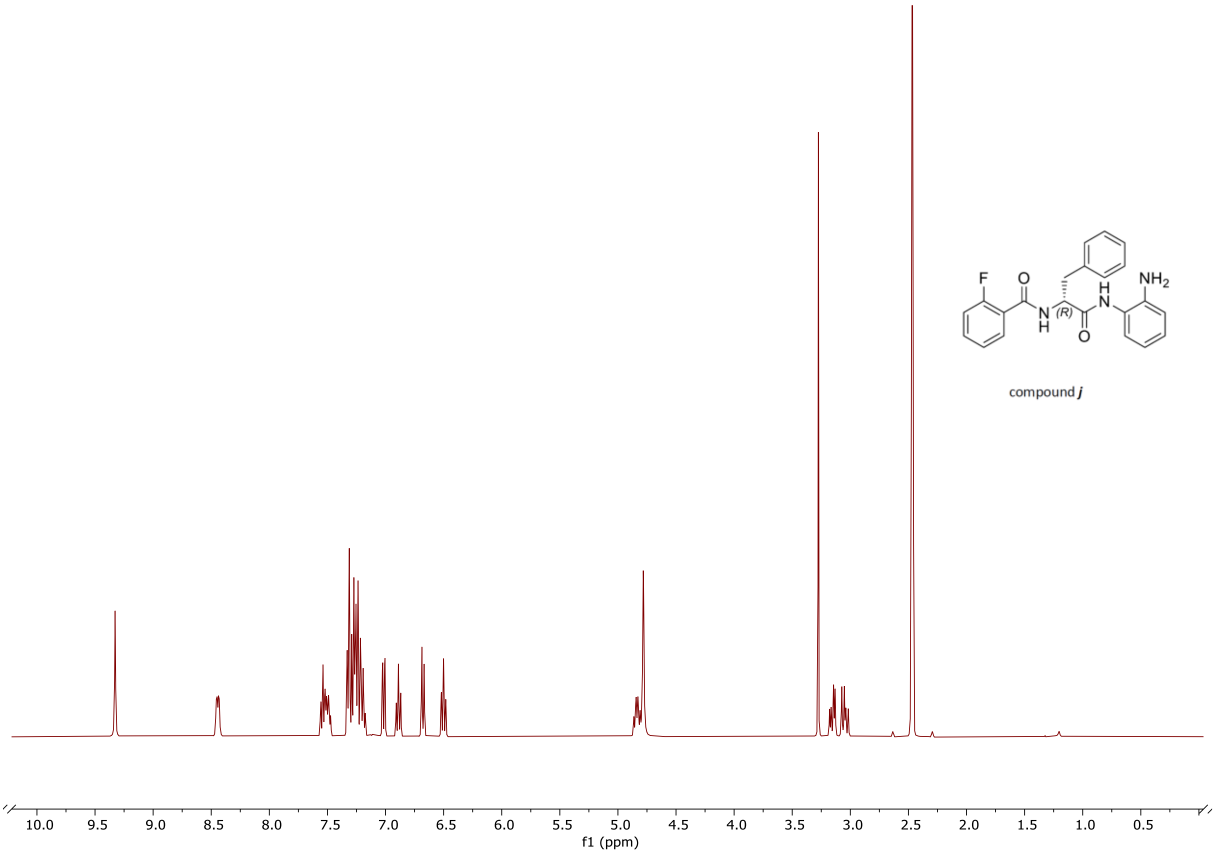

Figure S12. ^{1}H NMR spectrum (402 MHz, DMSO D6) of compound ***j***

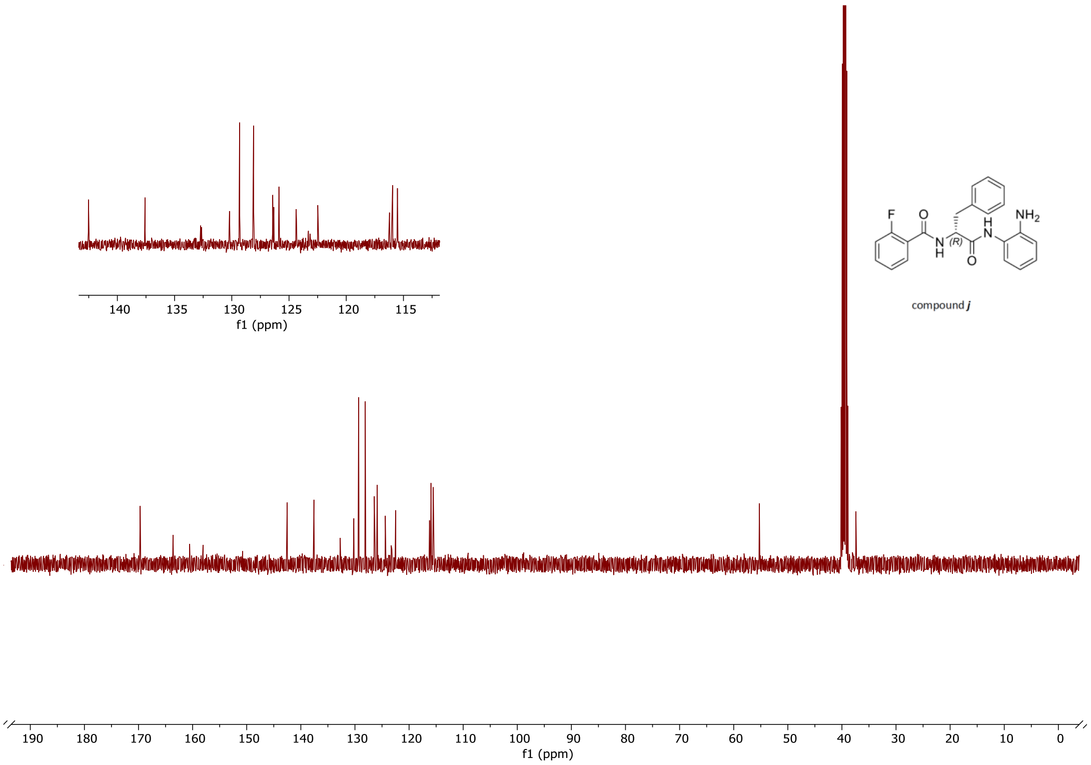

Figure S13. ^{13}C NMR spectrum (101 MHz, DMSO D6) of compound ***j***

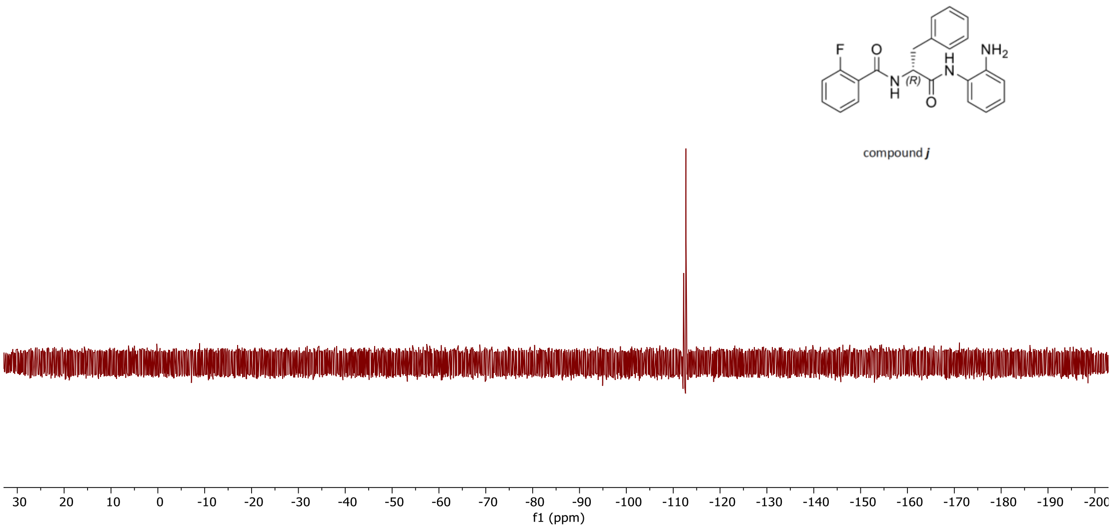

Figure S14. ^{19}F NMR spectrum (378 MHz, CD_3CN) of compound ***j***

4. HRMS mass spectra and HPLC chromatograms of the *in vitro* test compounds

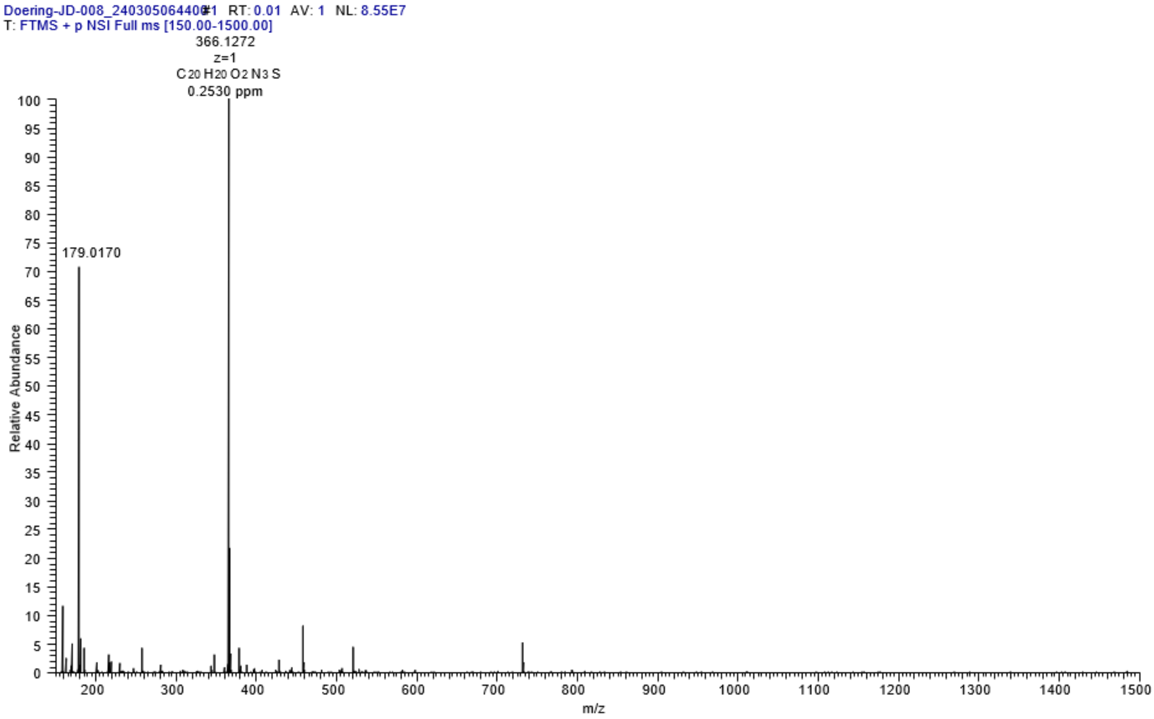

Figure S15. HRMS spectrum of compound ***d***.

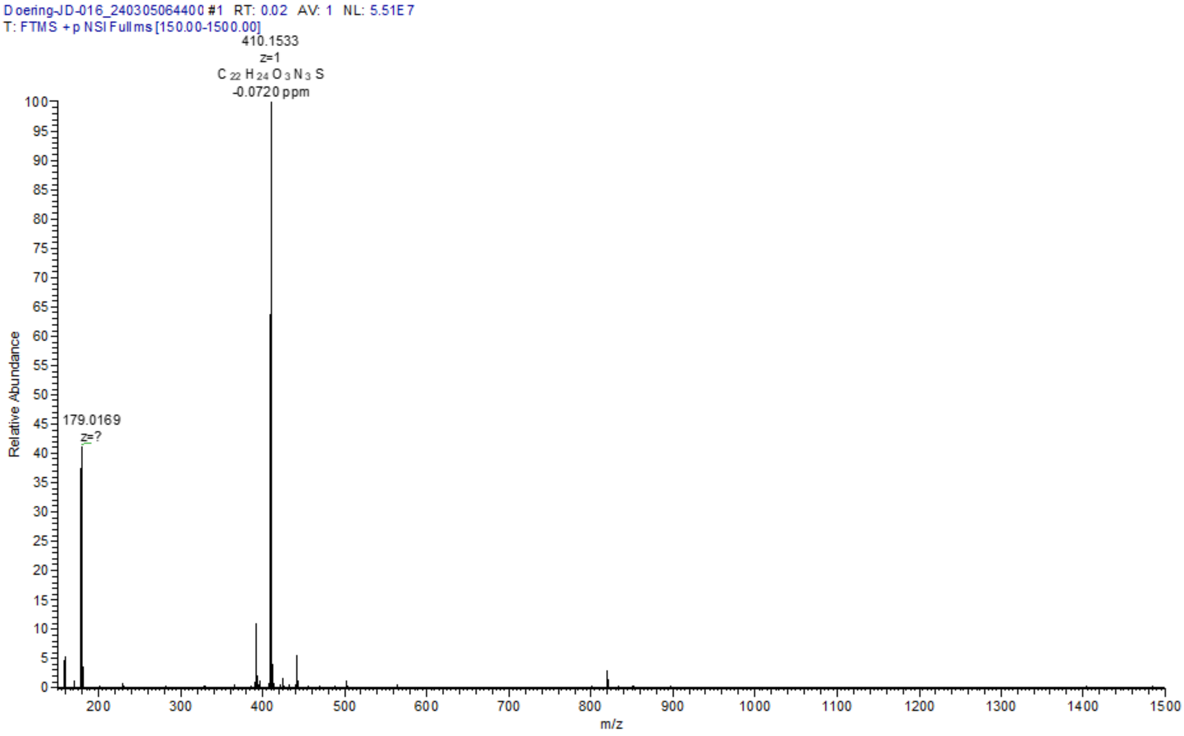

Figure S16. HRMS spectrum of compound ***e***.

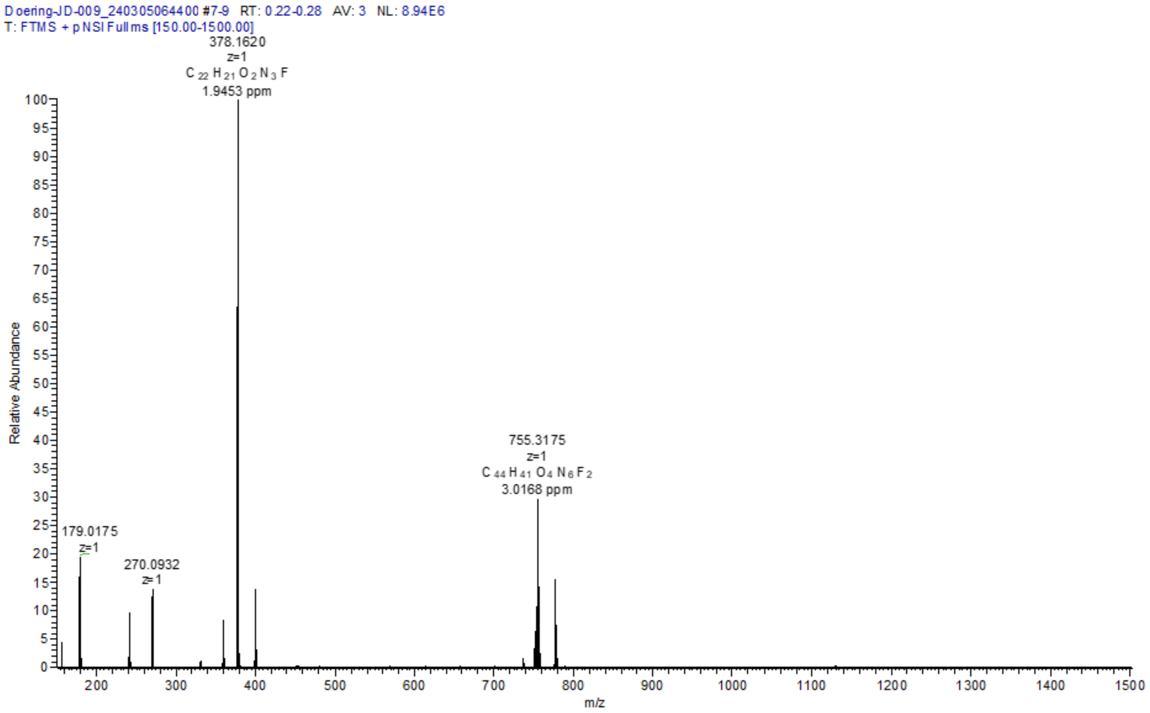

Figure S17. HRMS spectrum of compound ***j***.

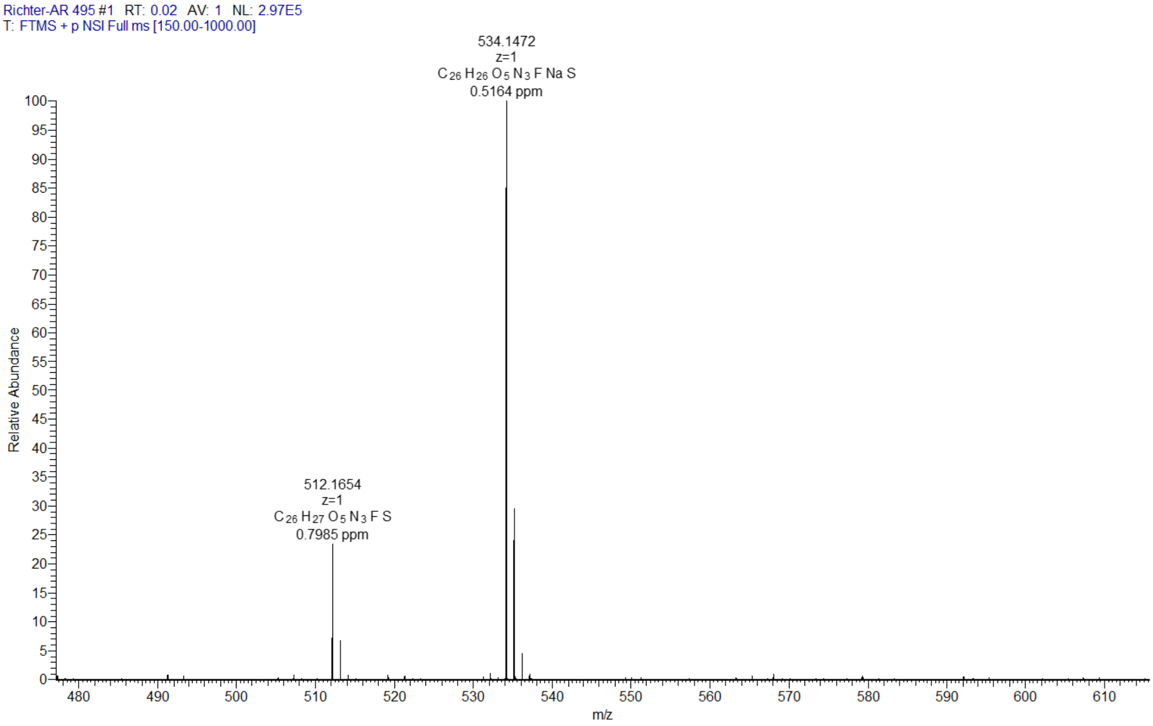

Figure S18. HRMS spectrum of compound ***l***.

<Sample Information>

Sample Name	: JD008_HPLC_1
Sample ID	:
Data Filename	: JD008_HPLC_1_001.lcd
Method Filename	: M_5-95_1.2_15min.lcm
Batch Filename	: 071223.lcb
Vial #	: 1-20
Sample Type	: Unknown
Injection Volume	: 3 uL
Date Acquired	: 12/8/2023 2:42:41 PM
Acquired by	: System Administrator
Date Processed	: 12/8/2023 4:40:48 PM
Processed by	: System Administrator

<Chromatogram>

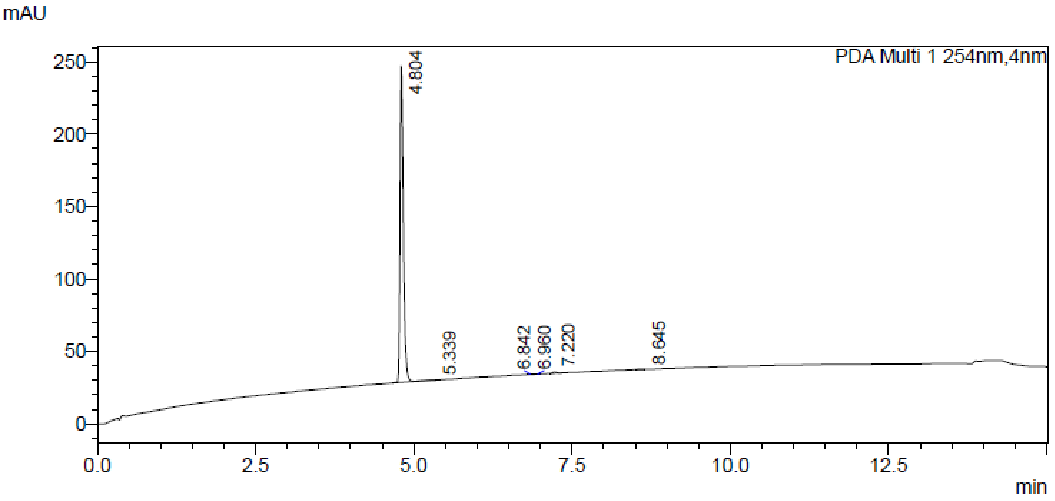

<Peak Table>

PDA Ch1 254nm

Peak#	Ret. Time	Area	Height	Area%
1	4.804	792010	218598	99.186
2	5.339	1684	-13	0.211
3	6.842	848	209	0.106
4	6.960	9	-14	0.001
5	7.220	3558	933	0.446
6	8.645	401	-7	0.050
Total		798510	219706	100.000

Figure S19. HPLC trace of compound ***d***.

<Sample Information>

Sample Name	: JD016_3µl_3		
Sample ID	:		
Data Filename	: Jd016_3µl_2.lcd		
Method Filename	: M_5-95_1.2_6min.lcm		
Batch Filename	:		
Vial #	: 1-17	Sample Type	: Unknown
Injection Volume	: 3 uL		
Date Acquired	: 12/14/2023 2:40:23 PM	Acquired by	: System Administrator
Date Processed	: 2/9/2024 1:06:03 PM	Processed by	: System Administrator

<Chromatogram>

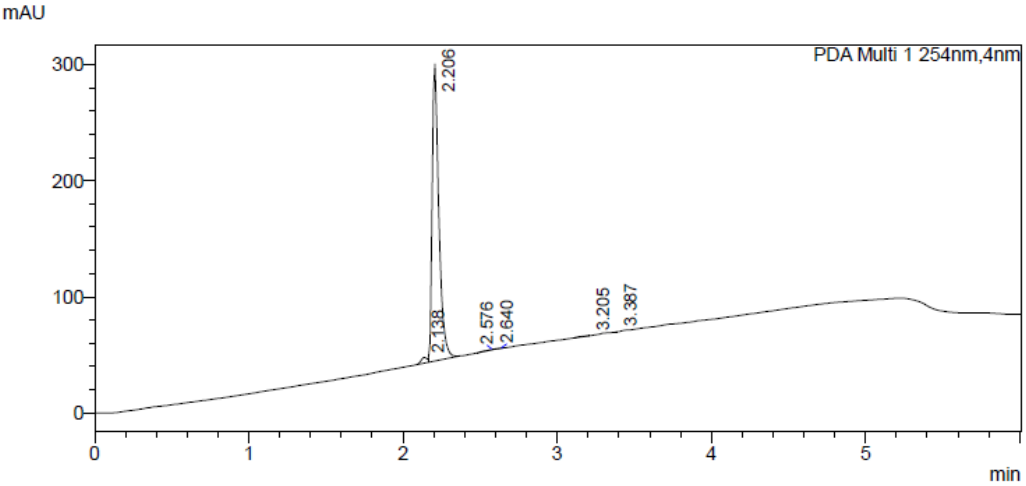

<Peak Table>

PDA Ch1 254nm

Peak#	Ret. Time	Area	Height	Area%
1	2.138	14505	4857	1.857
2	2.206	762744	255890	97.645
3	2.576	1671	-92	0.214
4	2.640	637	-57	0.082
5	3.205	1514	-87	0.194
6	3.387	67	-104	0.009
Total		781137	260407	100.000

Figure S20. HPLC trace of compound ***e***.

<Sample Information>

Sample Name	: JD009_HPLC_1
Sample ID	:
Data Filename	: JD009_HPLC_1_002.lcd
Method Filename	: M_5-95_1.2_15min.lcm
Batch Filename	: 071223.lcb
Vial #	: 1-21
Injection Volume	: 3 uL
Date Acquired	: 12/8/2023 3:01:09 PM
Date Processed	: 12/8/2023 3:37:56 PM

Sample Type	: Unknown
Acquired by	: System Administrator
Processed by	: System Administrator

<Chromatogram>

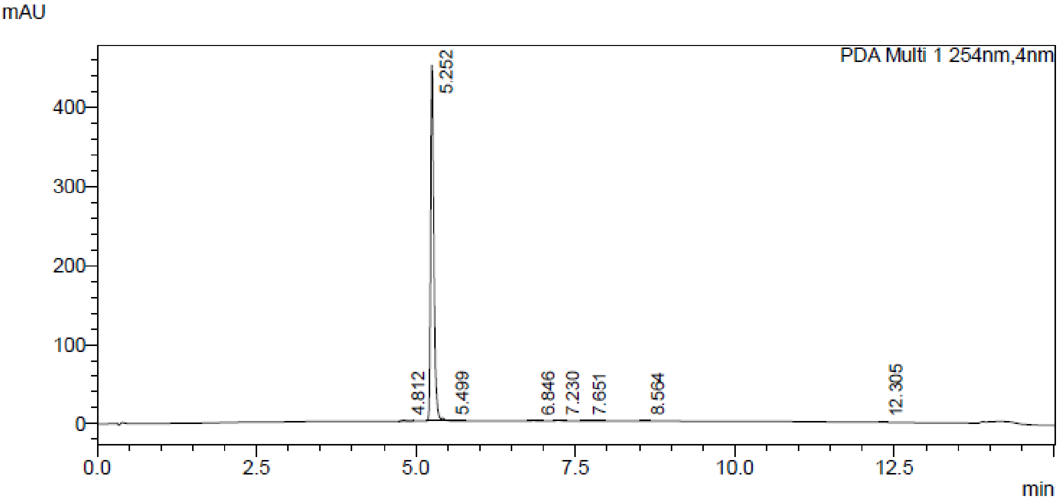

<Peak Table>

PDA Ch1 254nm

Peak#	Ret. Time	Area	Height	Area%
1	4.812	3193	919	0.193
2	5.252	1629882	450597	98.679
3	5.499	13893	458	0.841
4	6.846	527	159	0.032
5	7.230	2441	672	0.148
6	7.651	920	65	0.056
7	8.564	481	121	0.029
8	12.305	358	95	0.022
Total		1651695	453086	100.000

Figure S21. HPLC trace of compound ***j***.

<Sample Information>

Sample Name	: AR495		
Sample ID	:		
Data Filename	: AR495__004.lcd		
Method Filename	: M_5-95_1.2_6min.lcm		
Batch Filename	: AR495.lcb		
Vial #	: 2-45	Sample Type	: Unknown
Injection Volume	: 5 uL		
Date Acquired	: 3/20/2025 11:25:31 AM	Acquired by	: System Administrator
Date Processed	: 3/20/2025 11:31:32 AM	Processed by	: System Administrator

<Chromatogram>

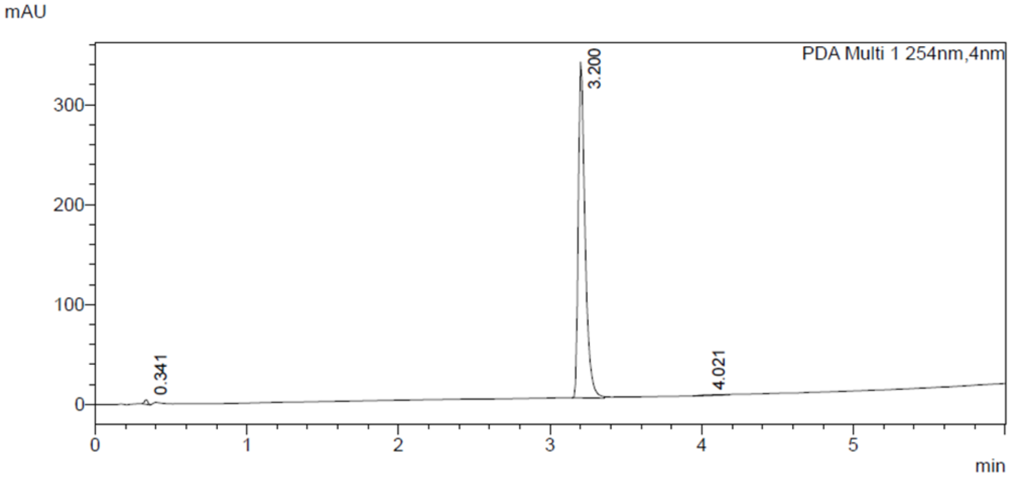

<Peak Table>

PDA Ch1 254nm

Peak#	Ret. Time	Area	Height	Area%
1	0.341	7186	4421	0.674
2	3.200	1050877	336141	98.637
3	4.021	7338	739	0.689
Total		1065401	341301	100.000

Figure S22. HPLC trace of compound ***I***.

5. LC-MS/MS Data for metabolite identification

Metabolite identification for MMV688845 after incubation with murine microsomes

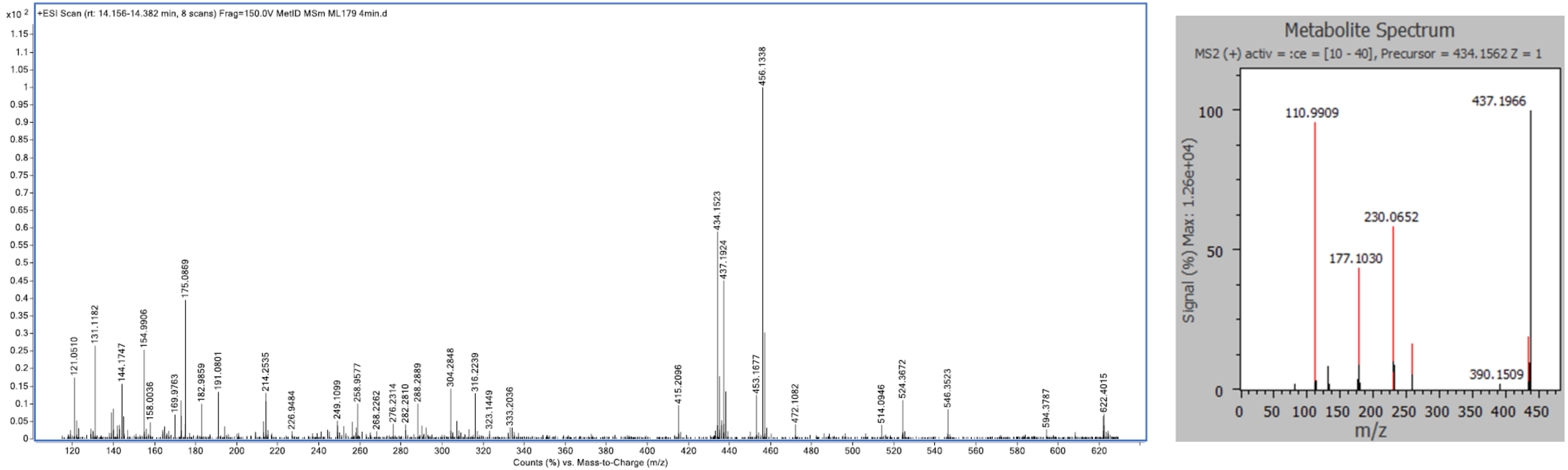

Figure S23. MS Data and fragmentation pattern of metabolite ***a***.

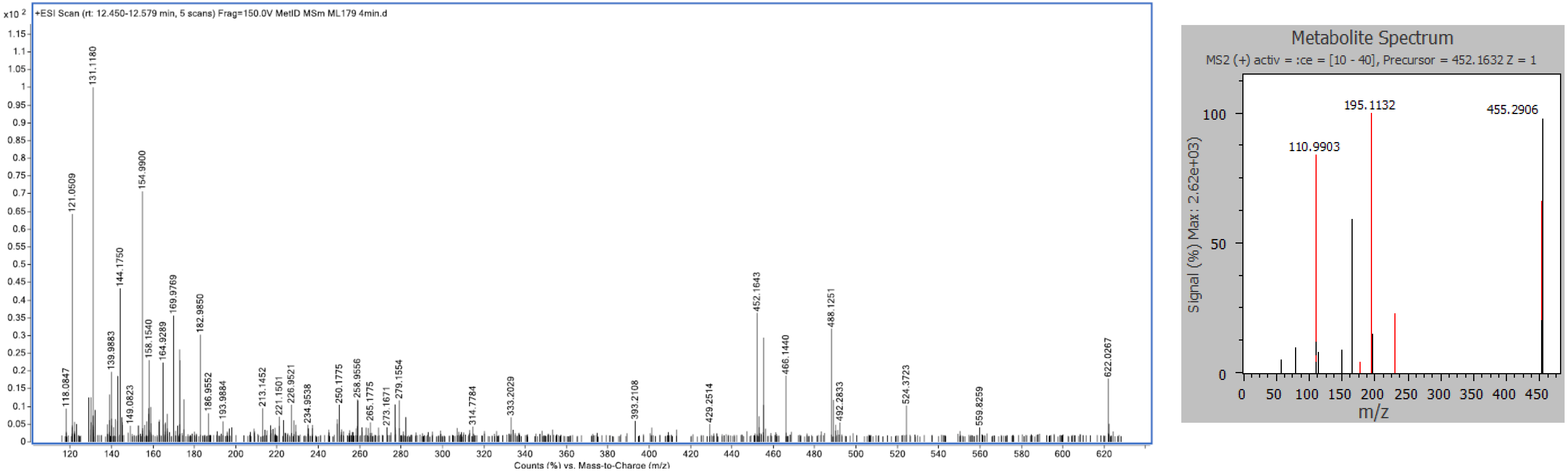

Figure S24. MS Data and fragmentation pattern of metabolite ***b*.**

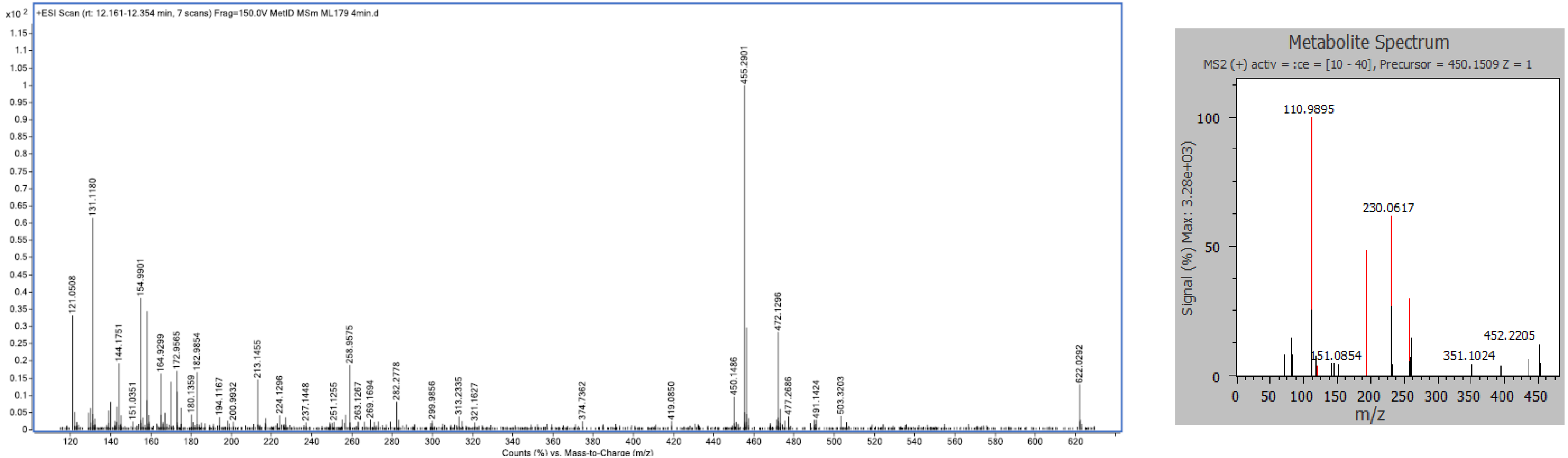

Figure S25. MS Data and fragmentation pattern of metabolite ***c.***

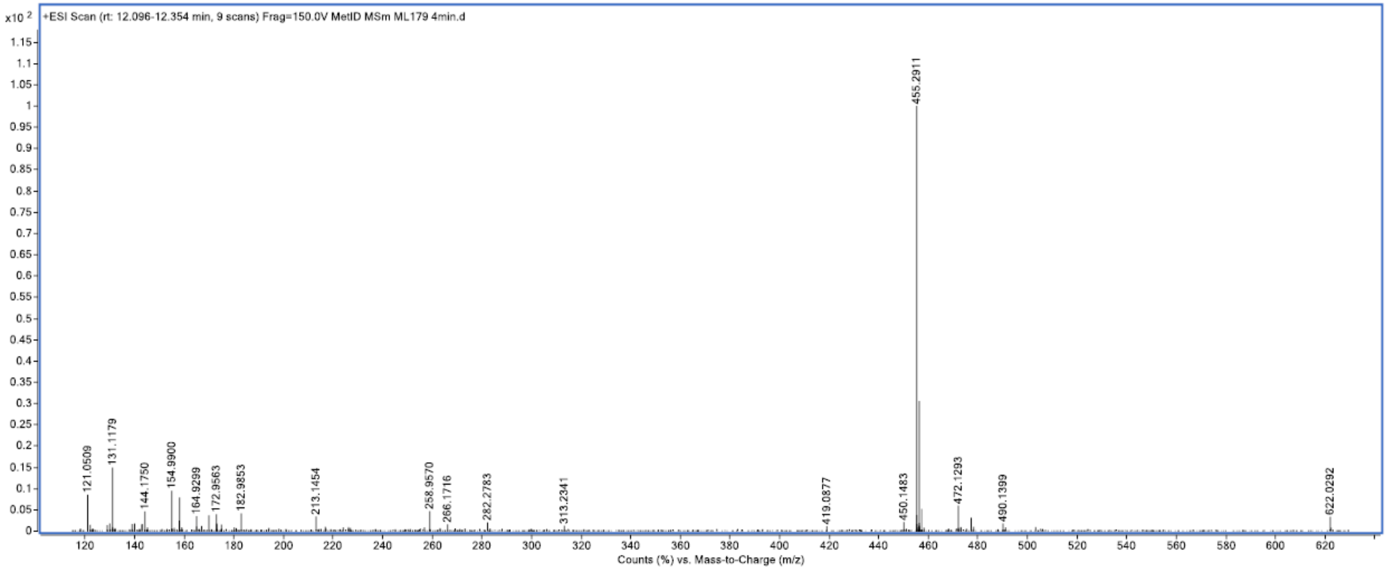

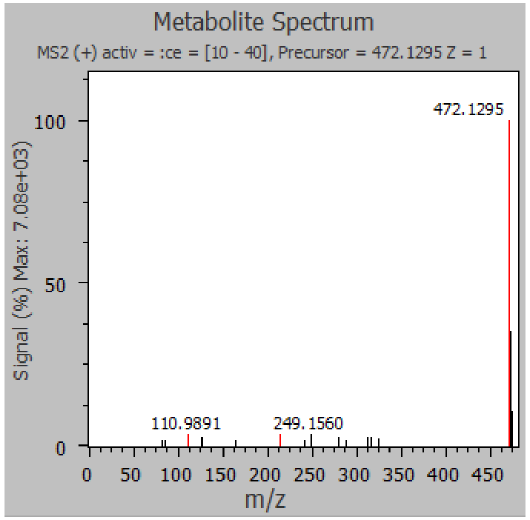

Figure S26. MS Data and fragmentation pattern of metabolite ***c+Na⁺.***

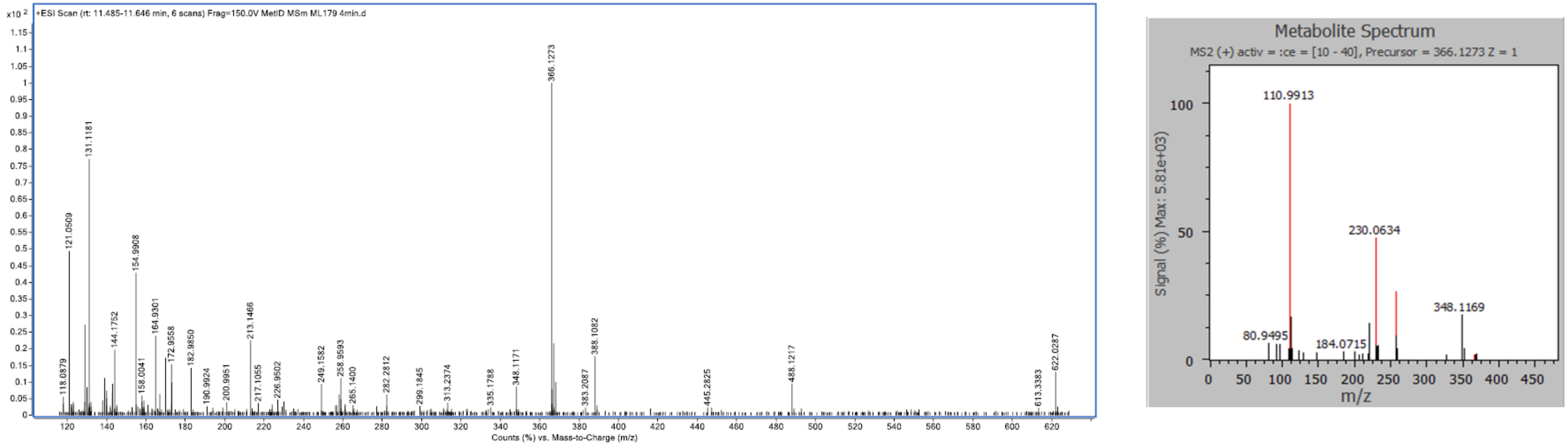

Figure S27. MS Data and fragmentation pattern of metabolite ***d.***

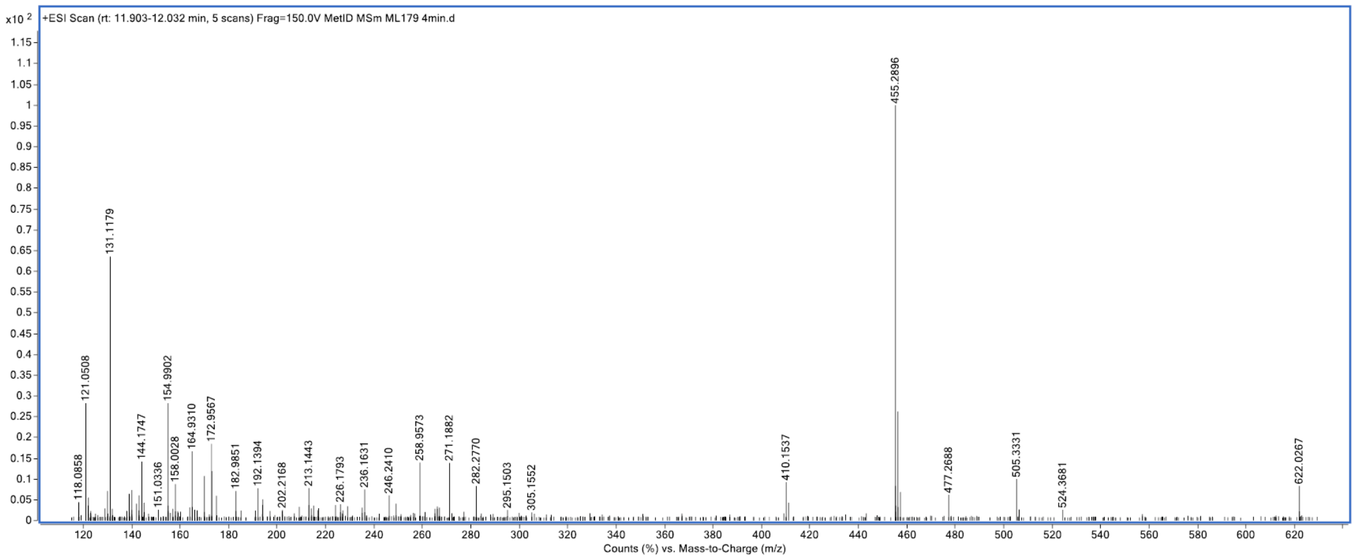

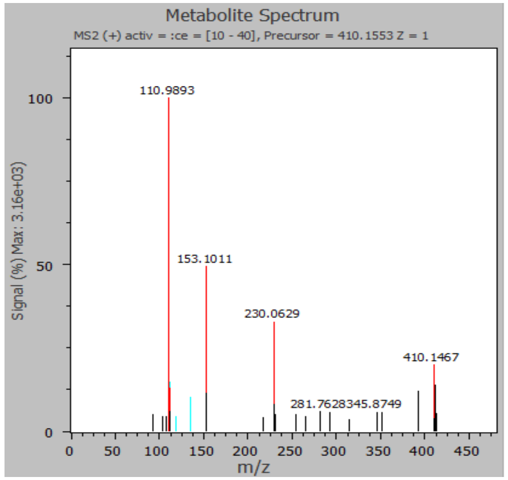

Figure S28. Fragmentation pattern of metabolite ***e.***

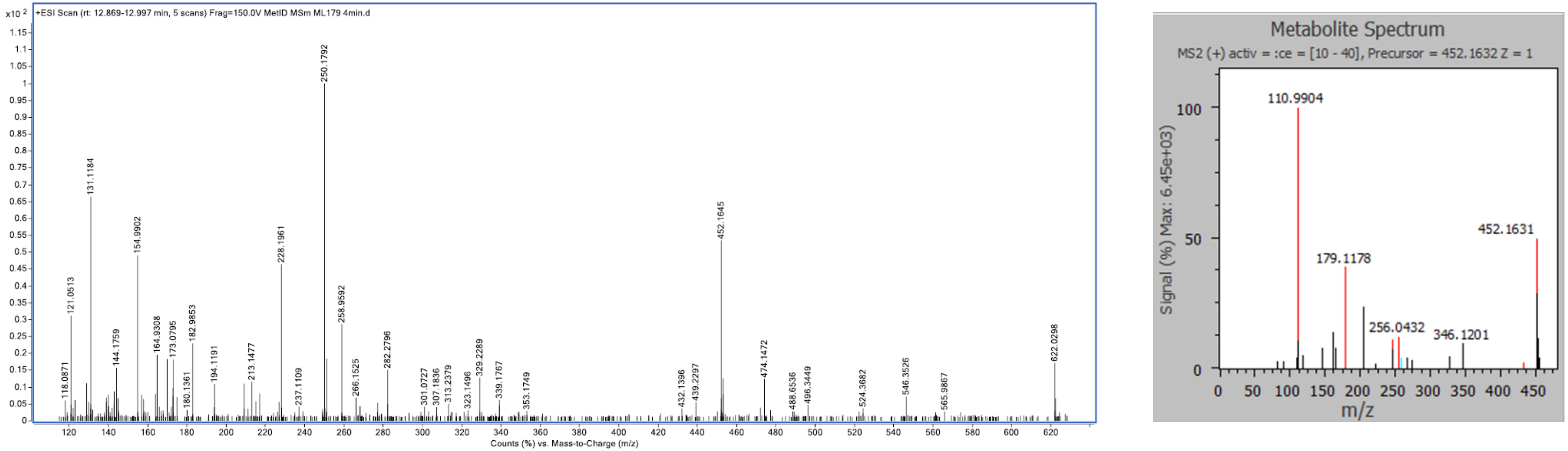

Figure S29. Fragmentation pattern of metabolite ***f*.**

Metabolite identification for AAP-SO_4 after incubation with murine microsomes

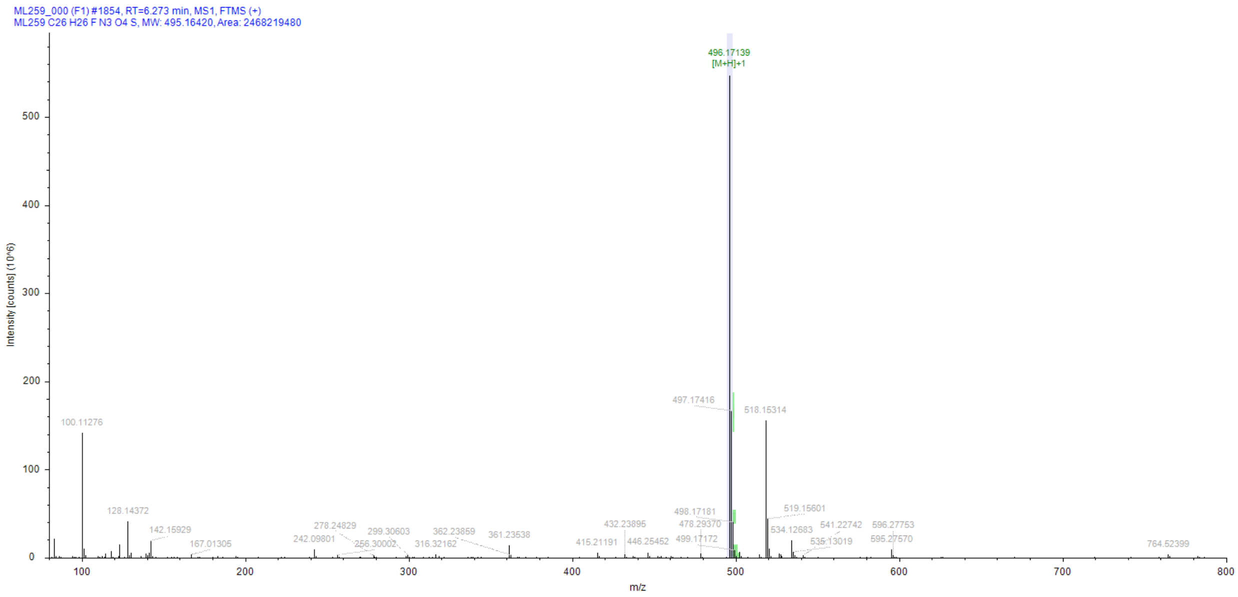

Figure S30. MS-Data of AAP-SO_2**.**

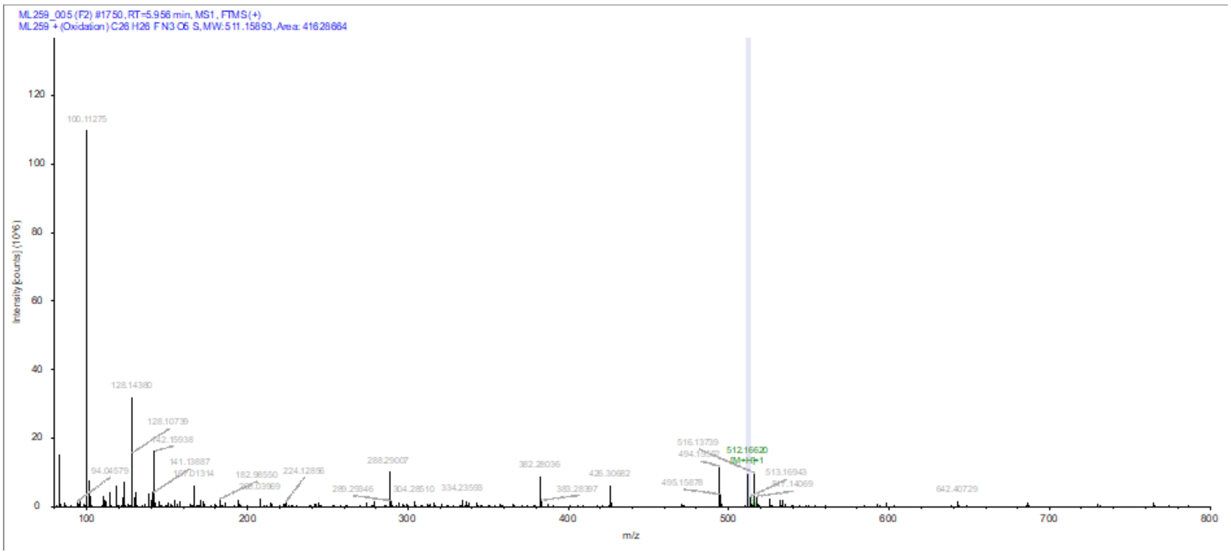

Figure S31. LC-MS/MS data of metabolite ***h.***

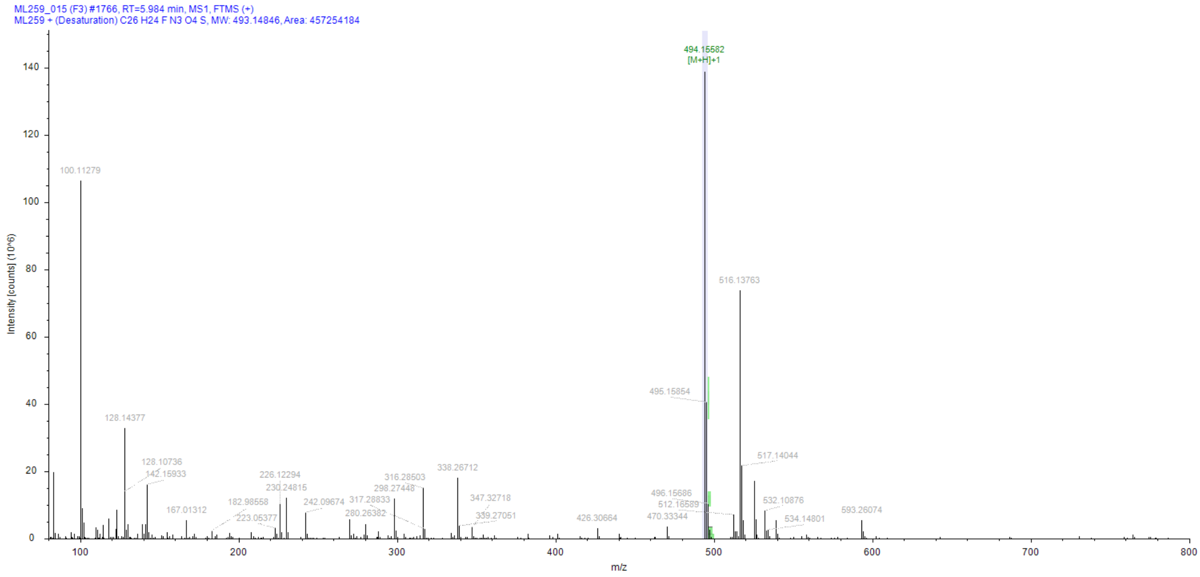

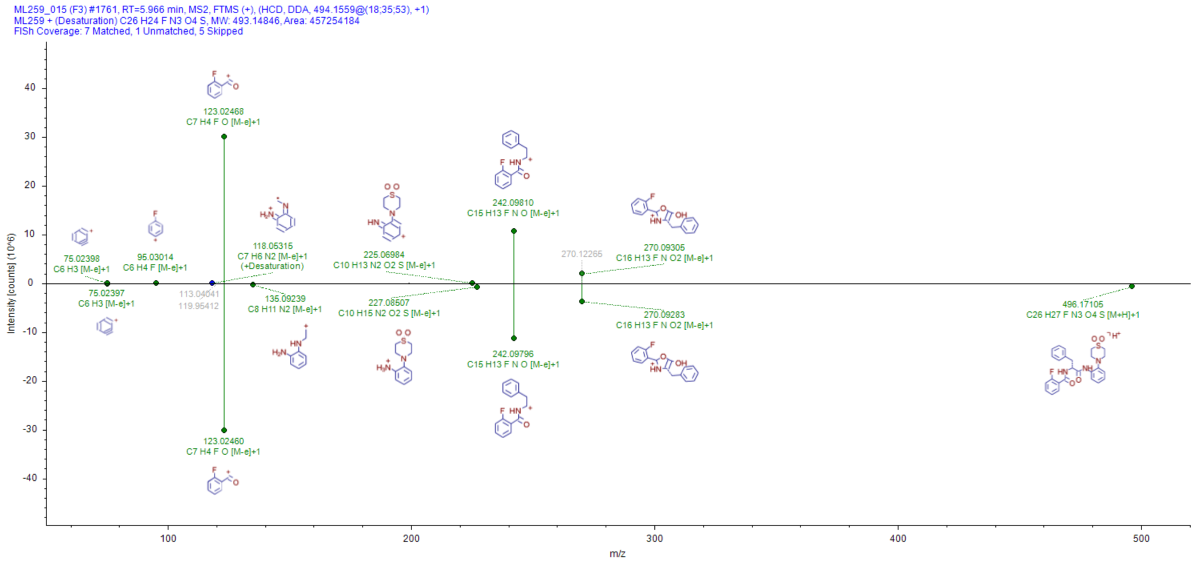

Figure S32. LC-MS/MS data of metabolite ***g.***

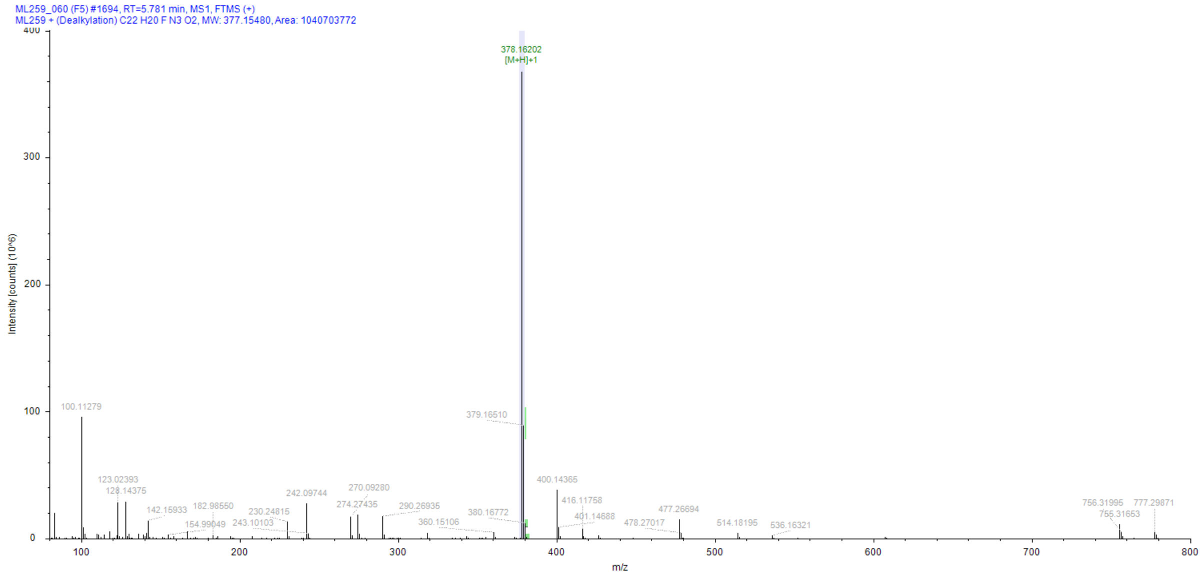

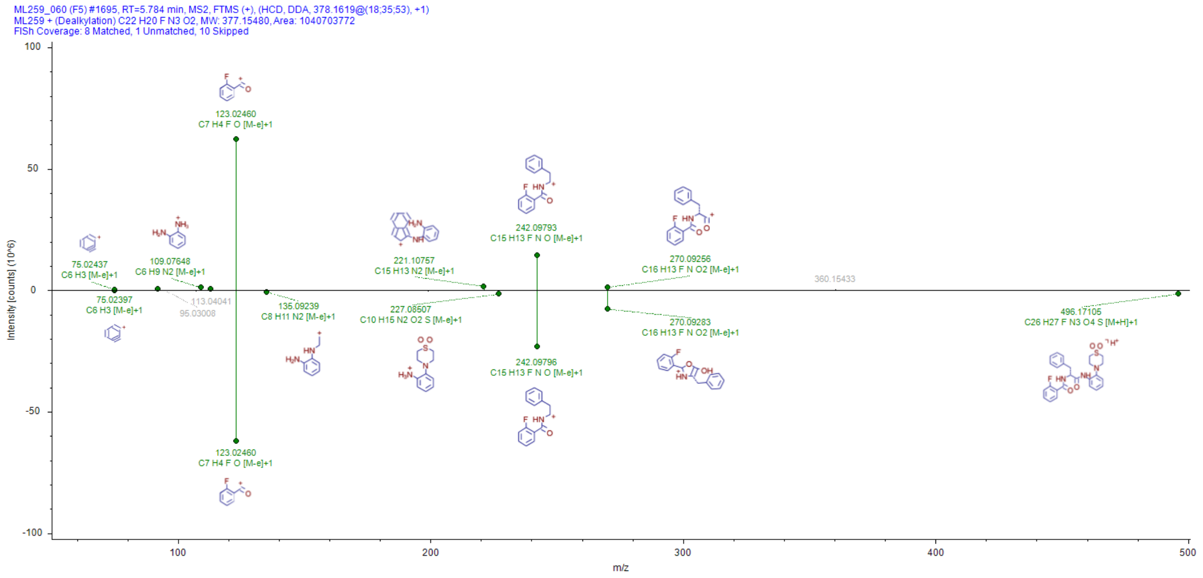

Figure S33. LC-MS/MS data of metabolite ***j.***

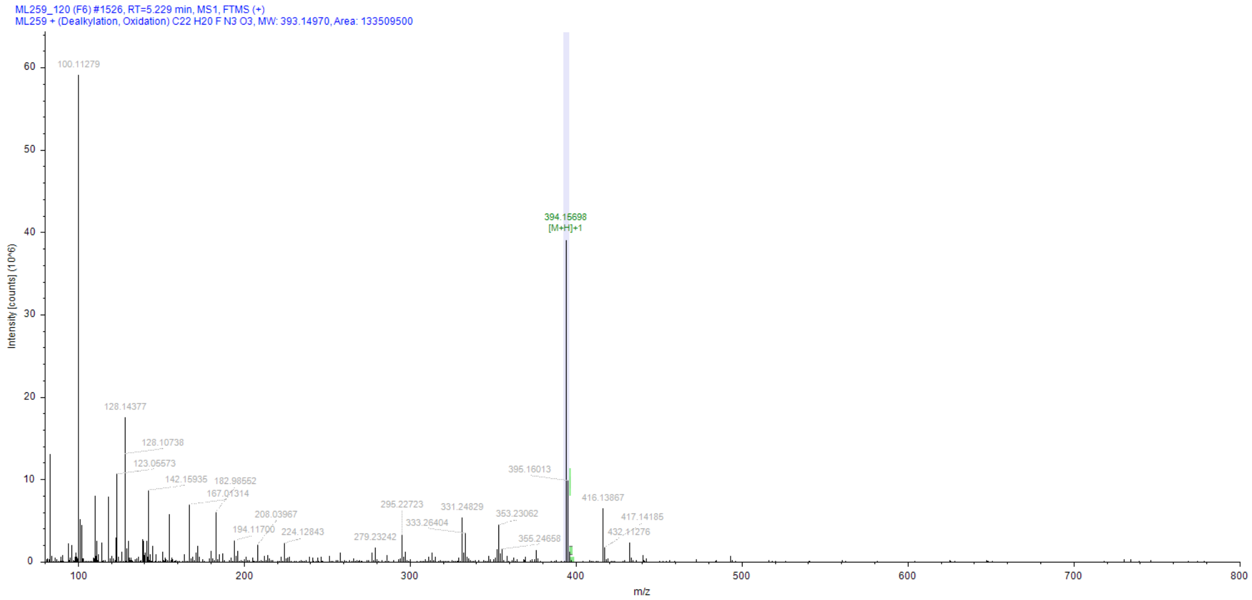

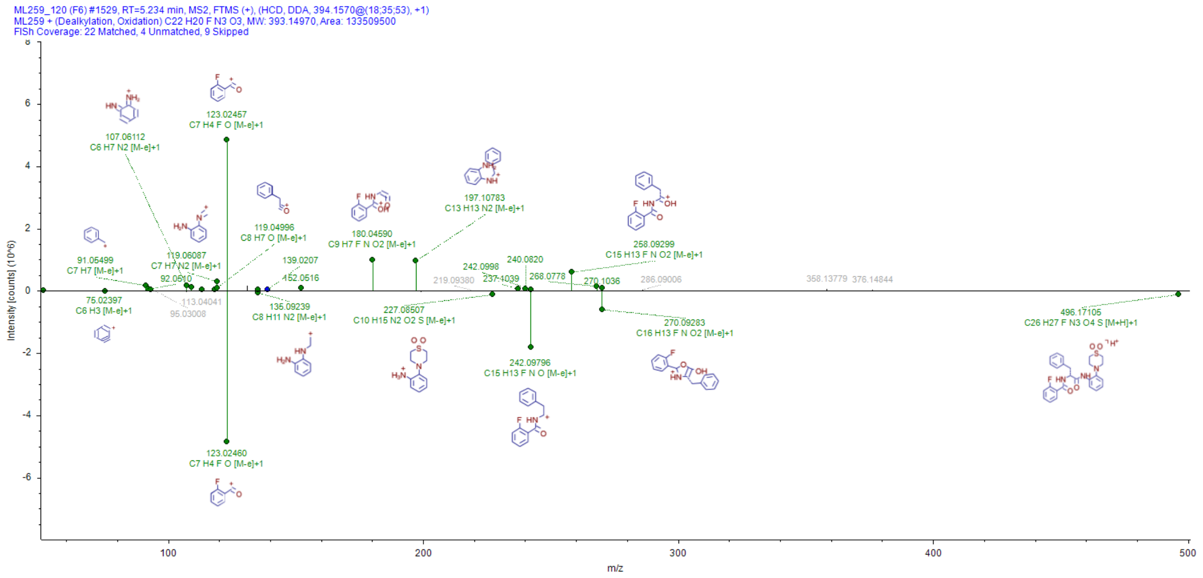

Figure S34. LC-MS/MS data of metabolite ***k.***

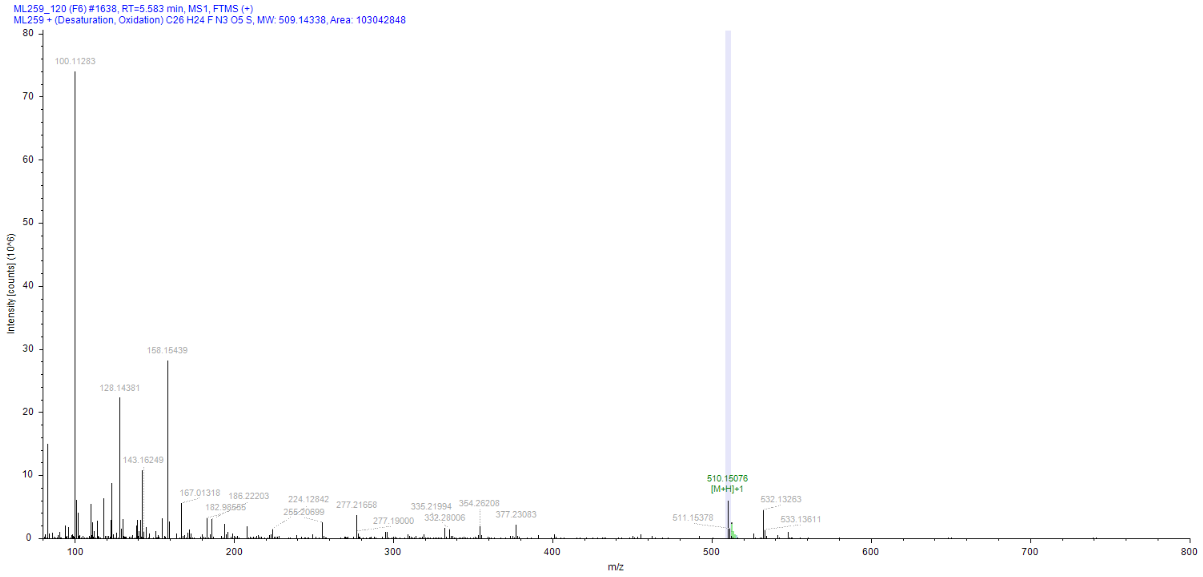

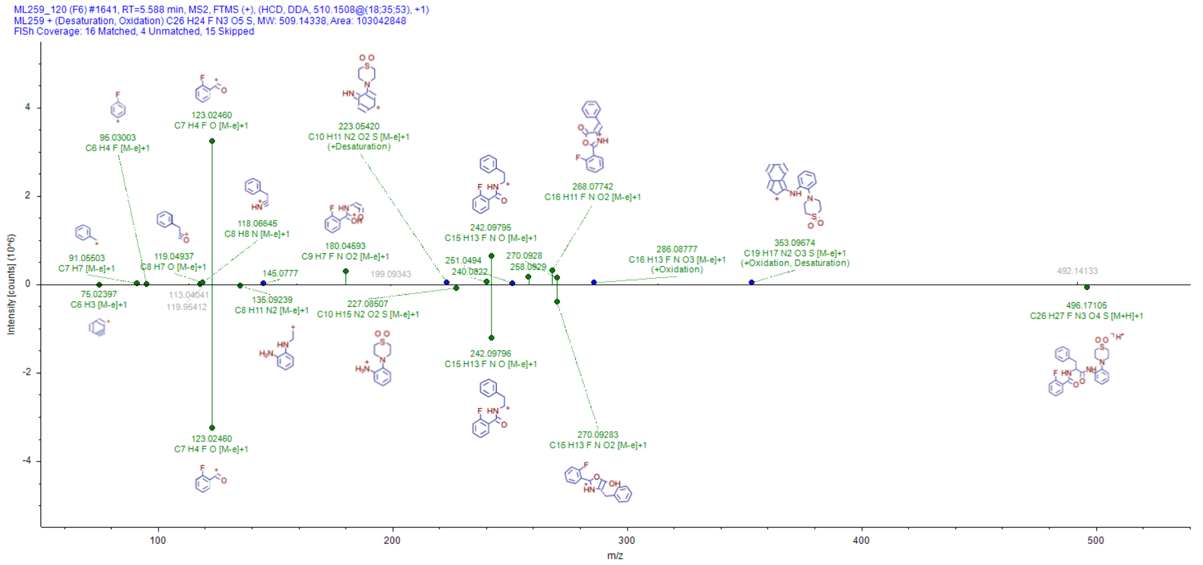

Figure S35. LC-MS/MS data of metabolite ***i.***

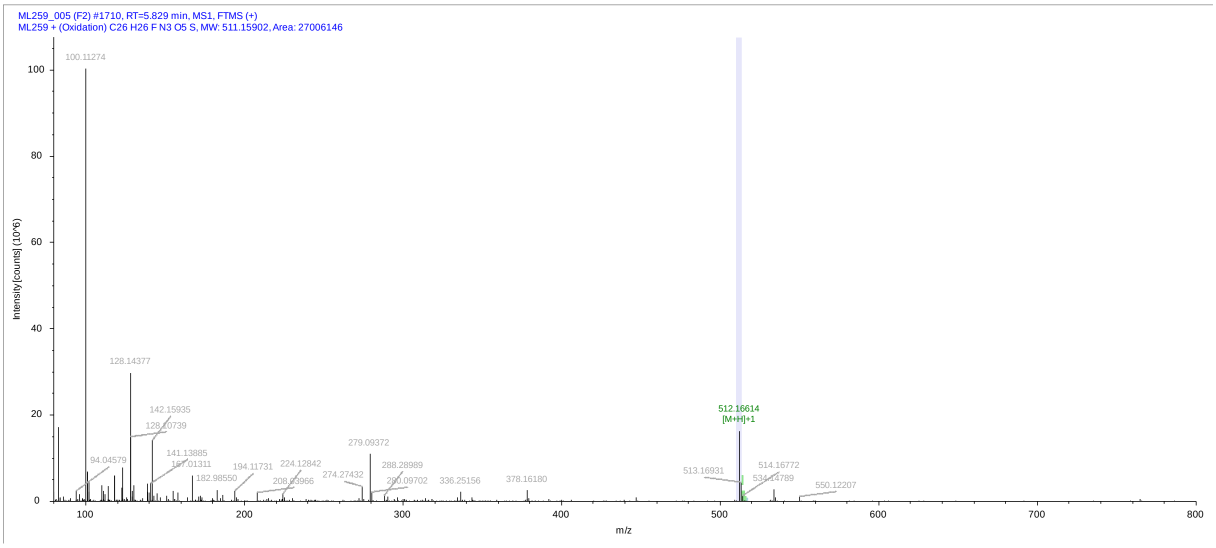

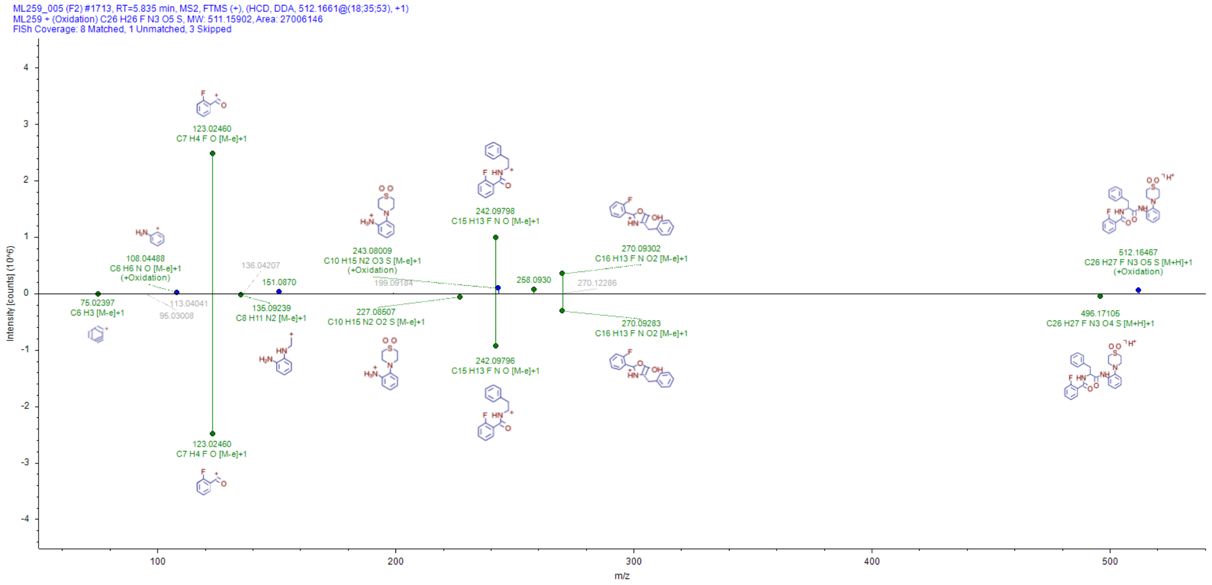

Figure S36. LC-MS/MS data of metabolite ***I*.**

Metabolite identification for AAP-SO_4 after incubation with human microsomes/CYP enzymes

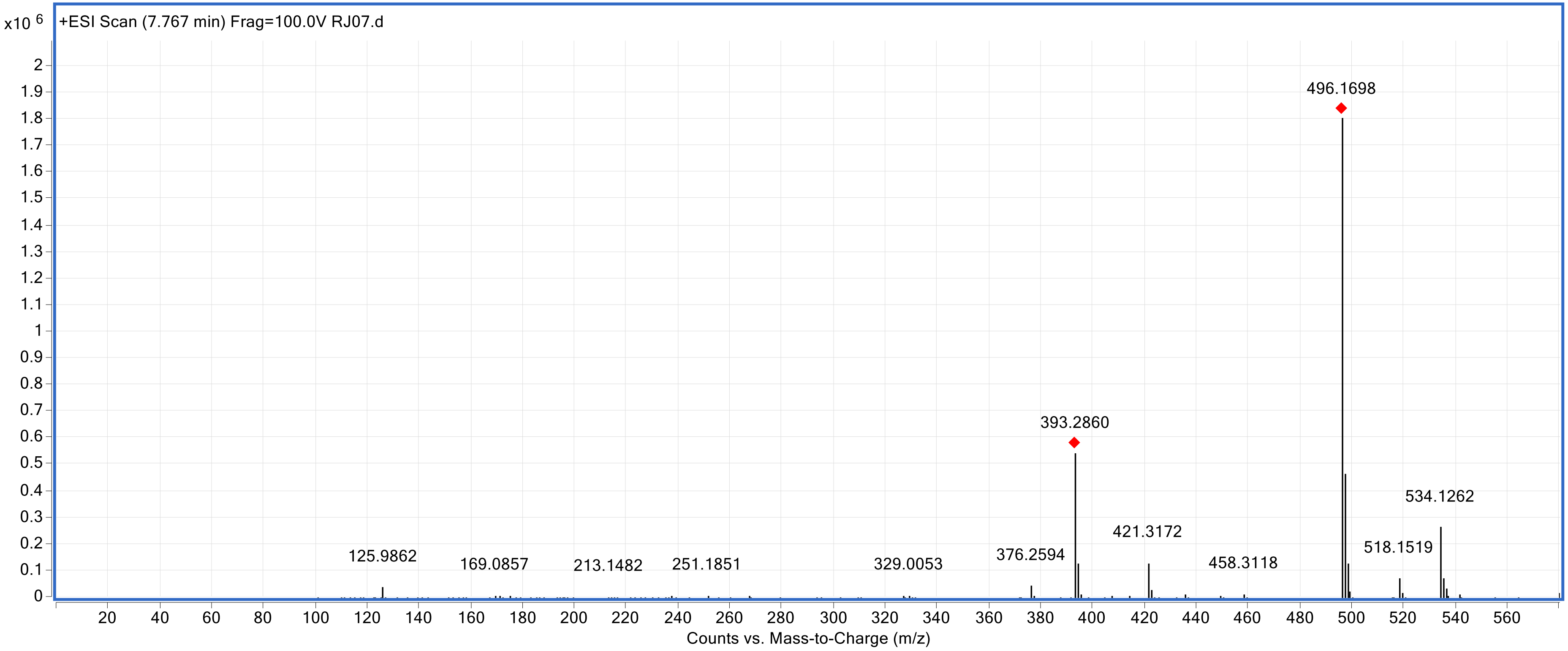

Figure S37. MS-Data of AAP-SO_2**.**

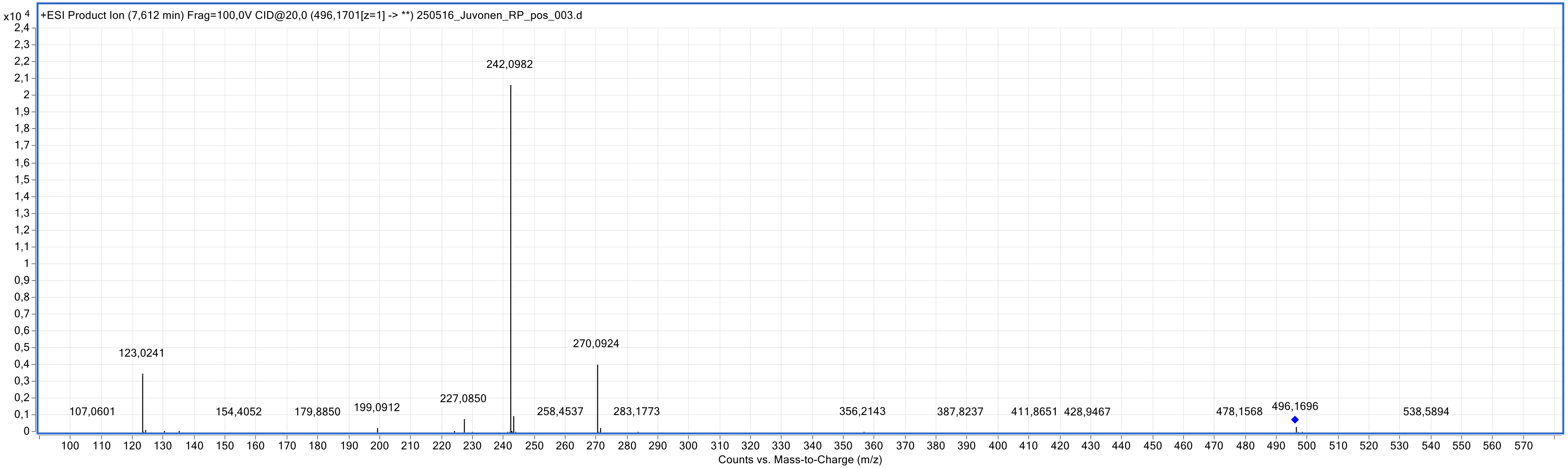

Figure S38. Fragmentation of AAP-SO_2**.**

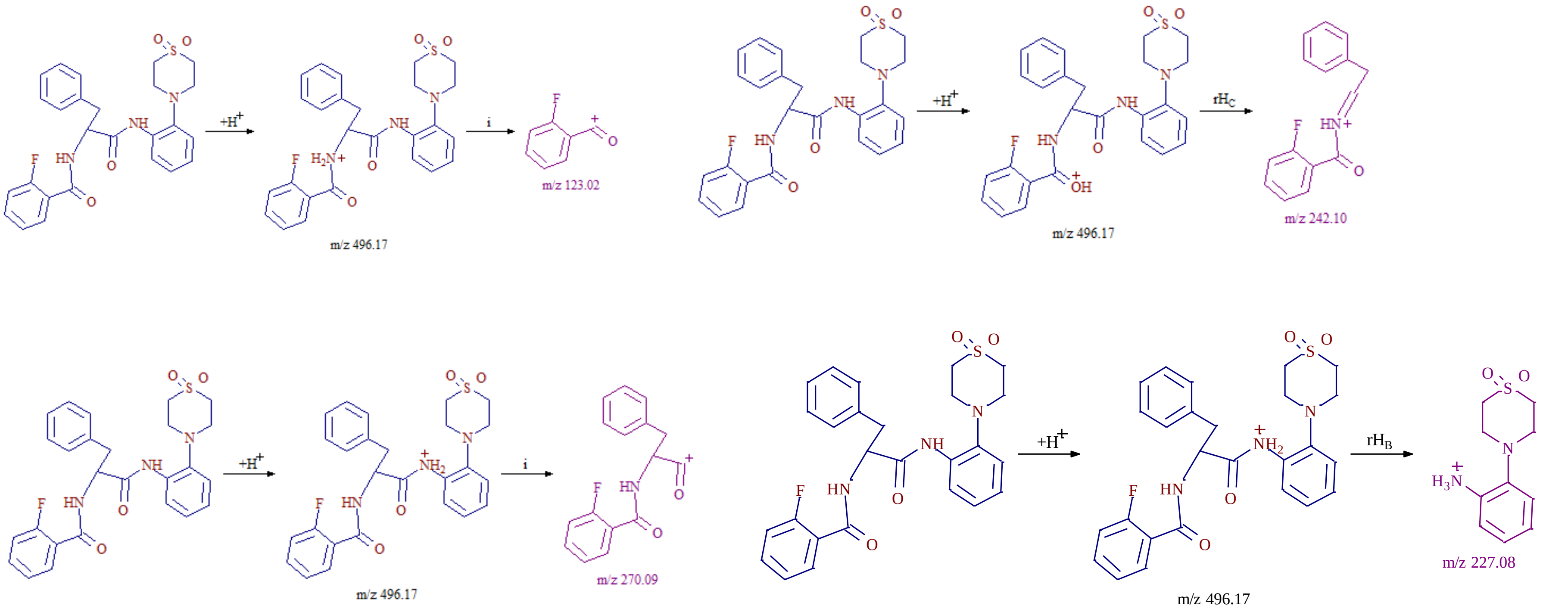

Figure S39. Full scan MS/MS spectrum of AAP-SO_2 and proposed structures of the main fragment ions. m/z ratio values are given with two significant digits in the fragmentation scheme and with four significant digits in the MS/MS spectrum.

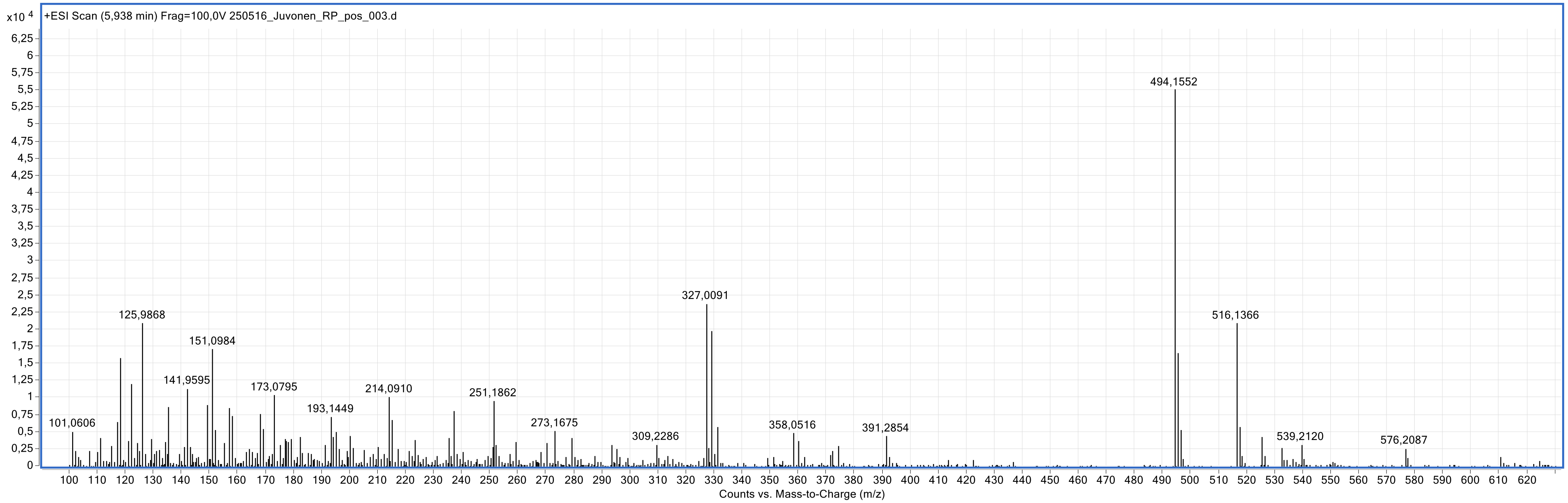

Figure S40. MS-Data of ***g***.

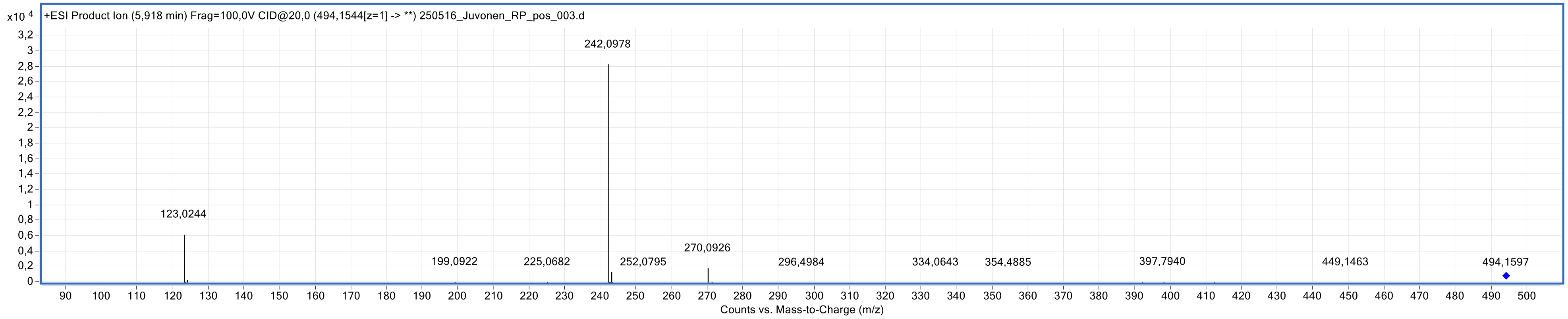

Figure S41. Fragmentation of ***g.***

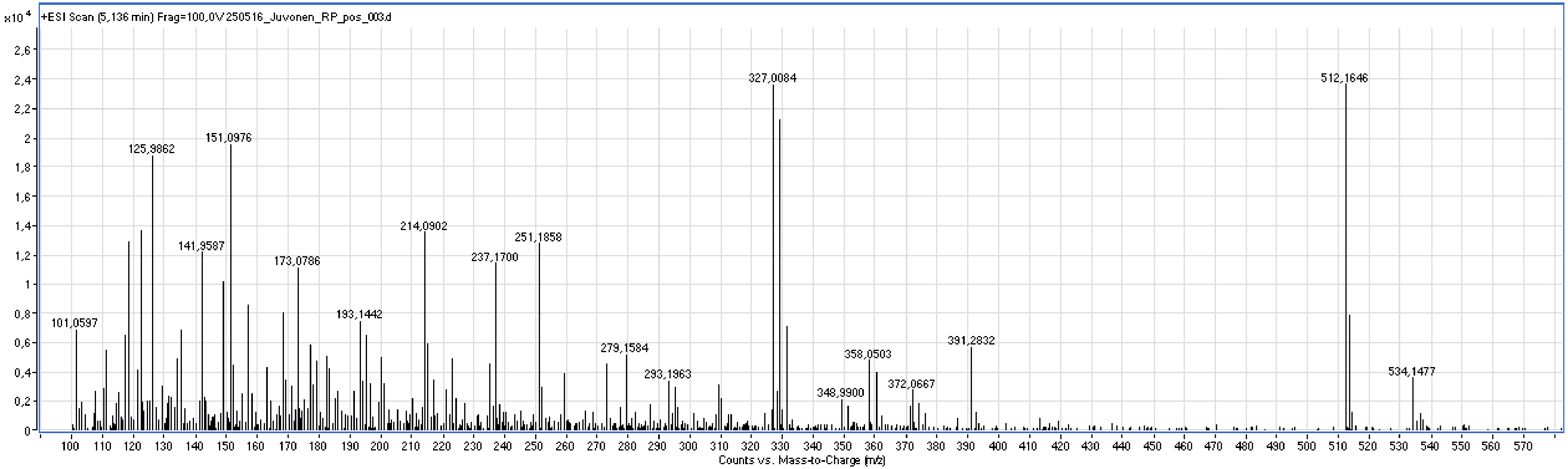

Figure S42. MS-Data of ***I.***

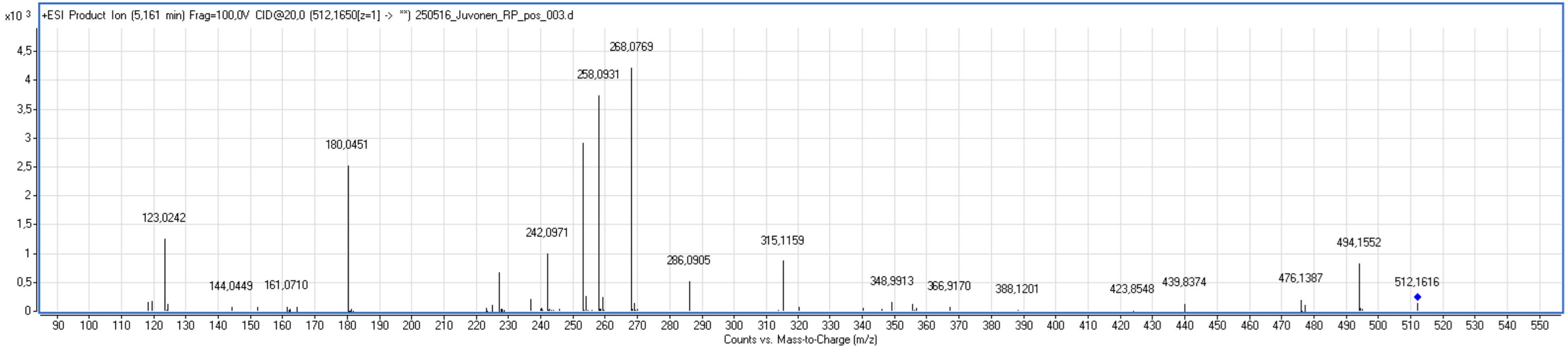

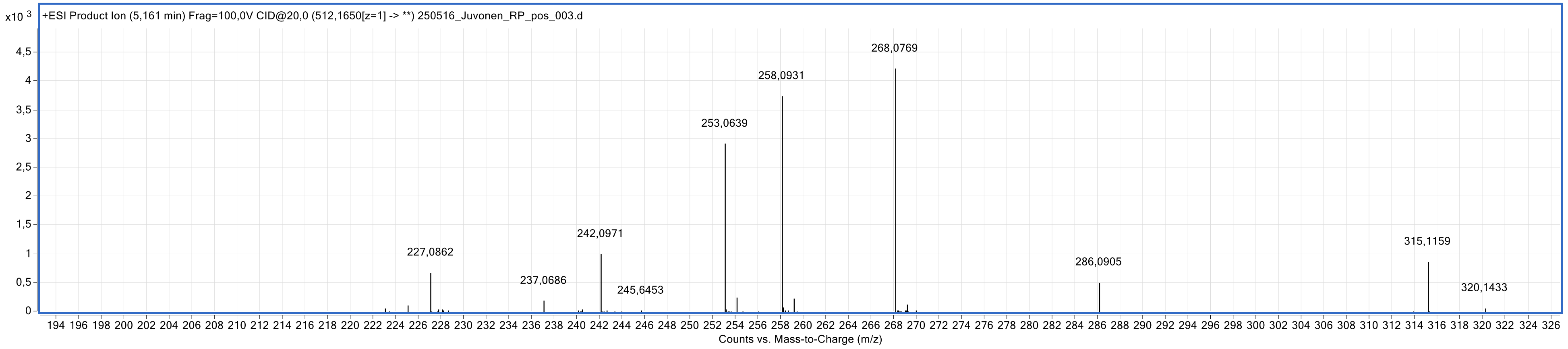

Figure S43. Fragmentation of ***I.***

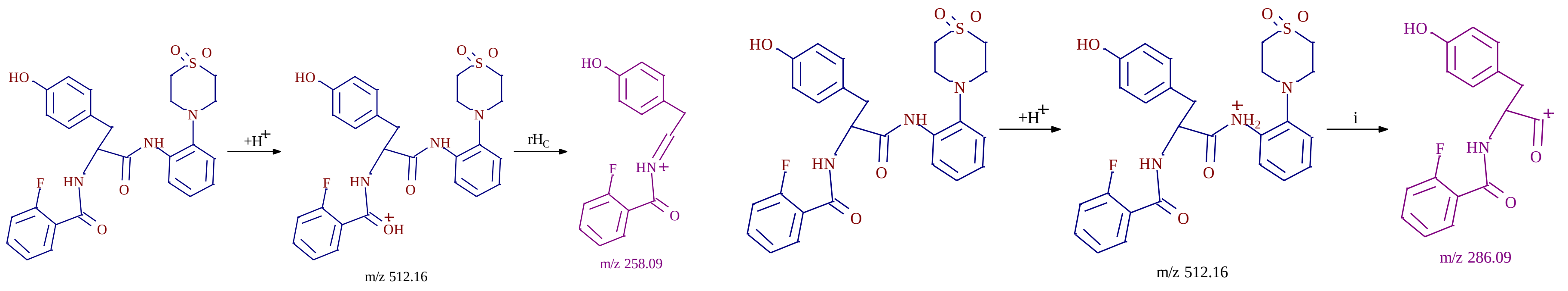

Figure S44. Full scan MS/MS spectrum of ***I*** and proposed structures of the main fragment ions. m/z ratio values are given with two significant digits in the fragmentation scheme and with four significant digits in the MS/MS spectrum.

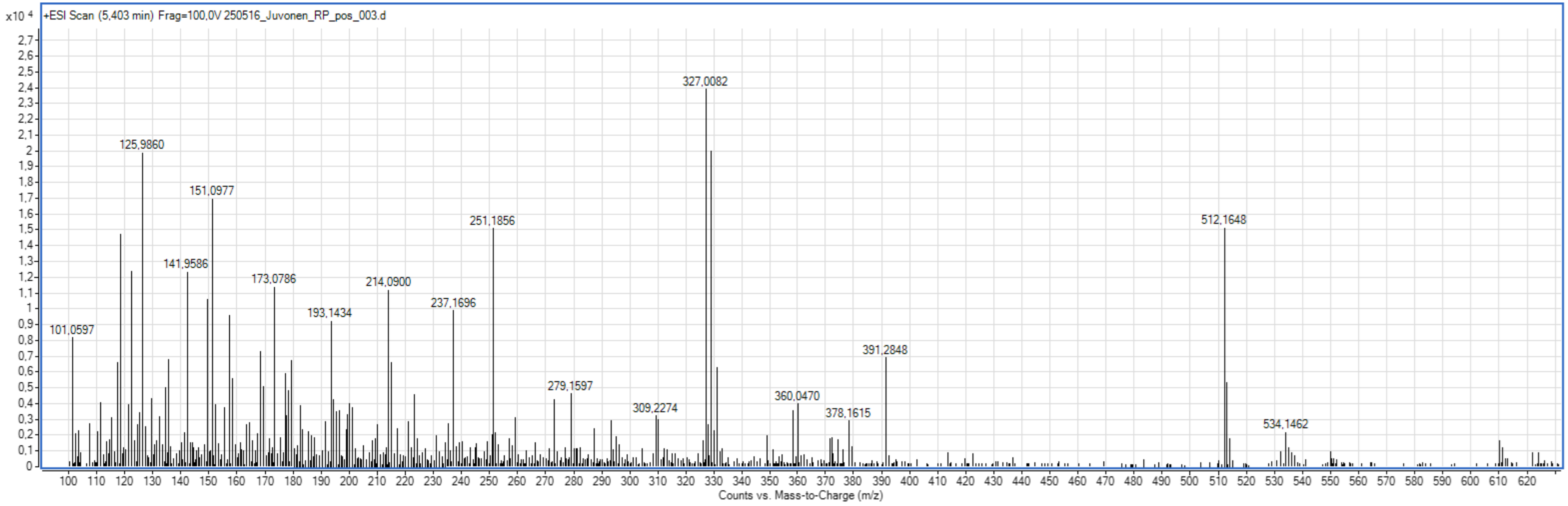

Figure S45. MS-Data of ***h.***

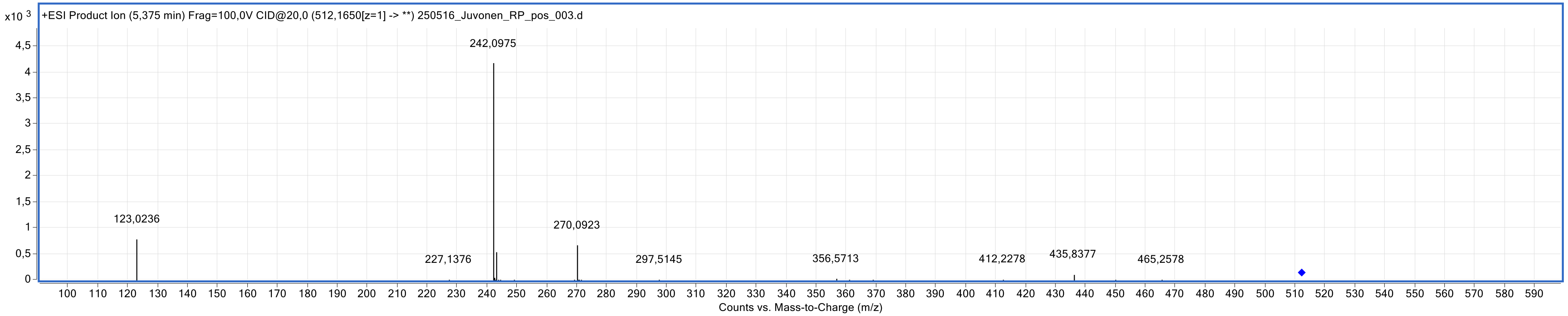

Figure S46. Fragmentation of ***h.***

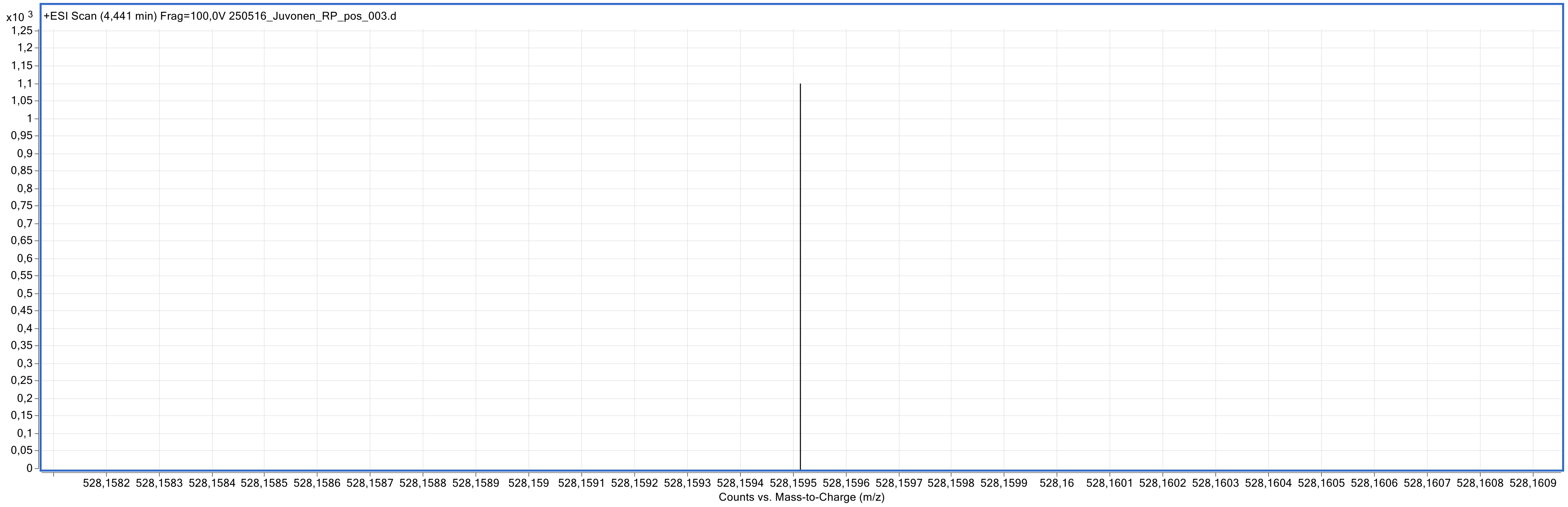

Figure S47. MS-Data of ***m.***

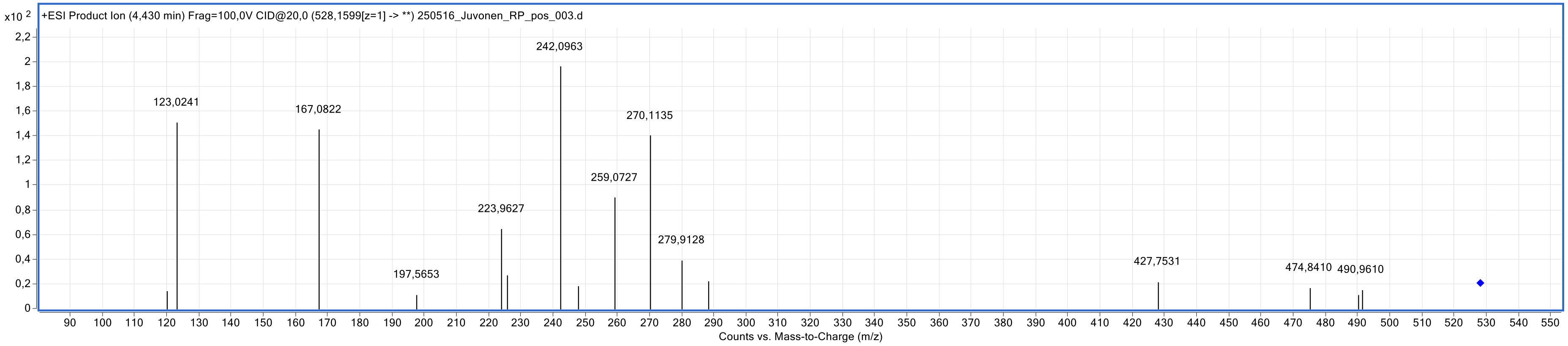

Figure S48. Fragmentation of ***m.***

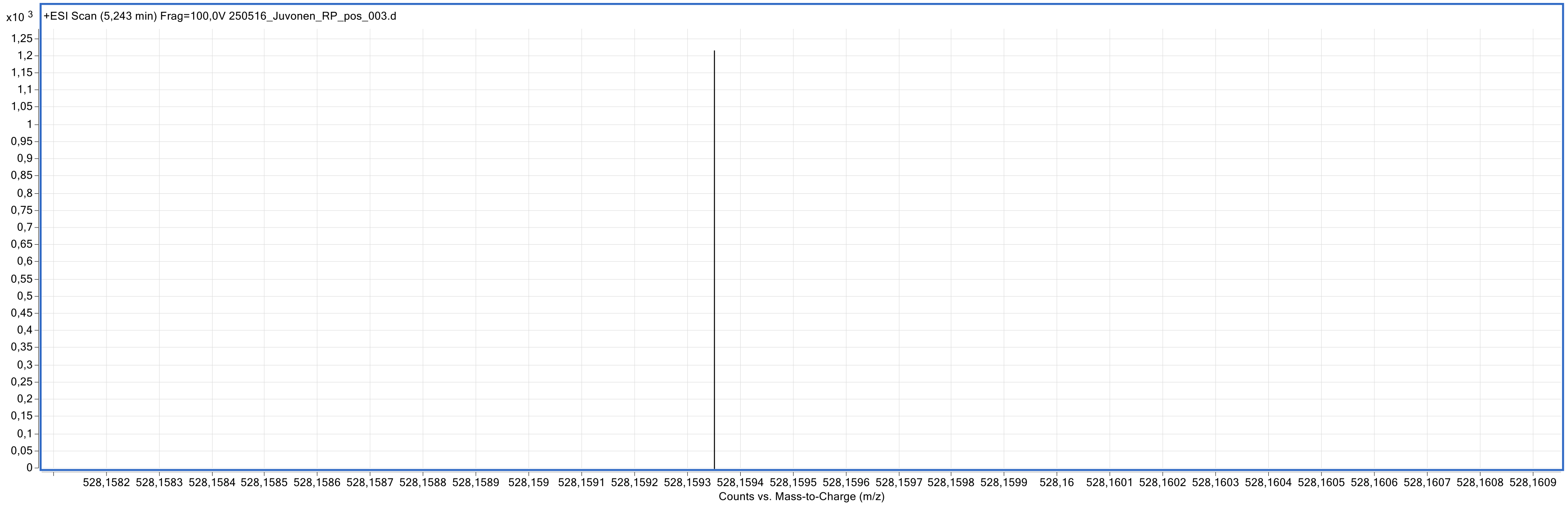

Figure S49. MS-Data of ***n.***

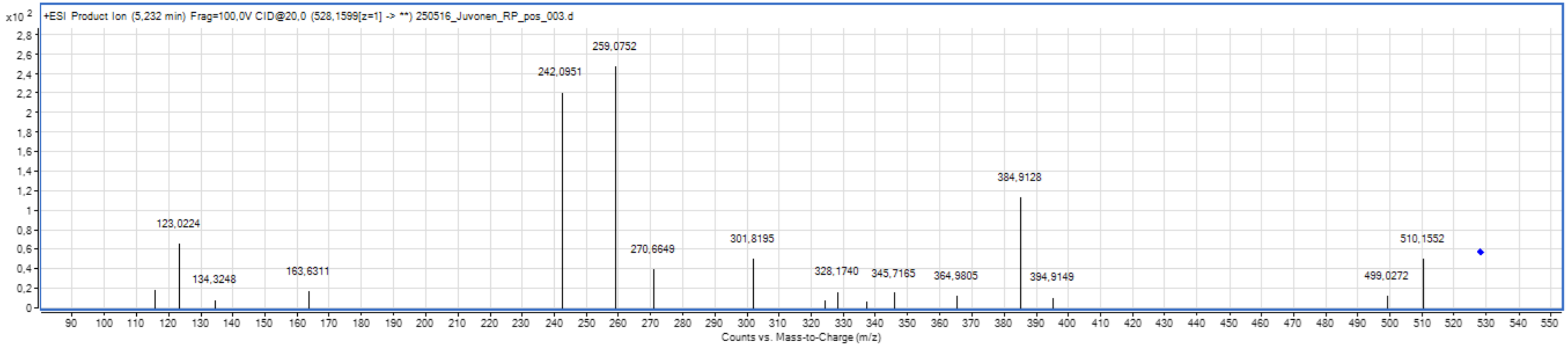

Figure S50. Fragmentation of ***n.***

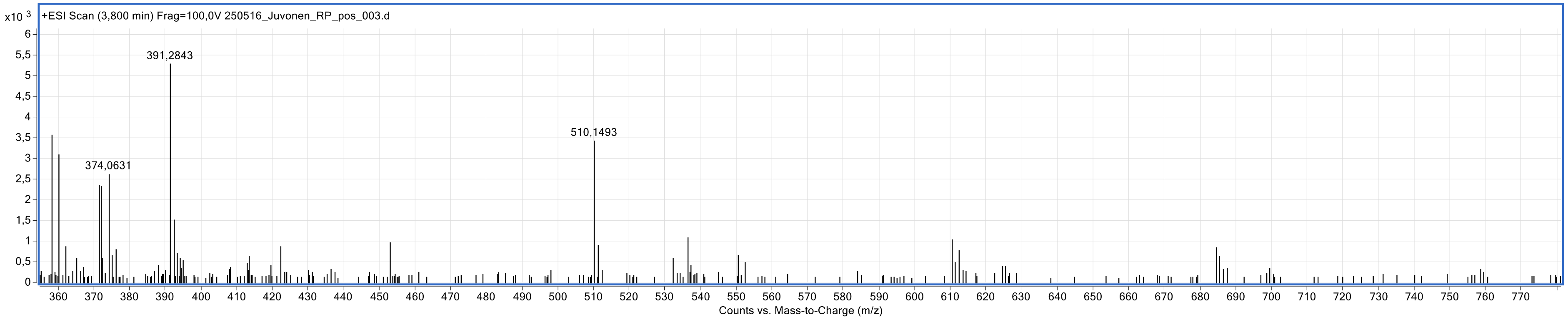

Figure S51. MS-Data of ***o*.**

References

(1) Lang, M.; Ganapathy, U. S.; Mann, L.; Abdelaziz, R.; Seidel, R. W.; Goddard, R.; Sequenzia, I.; Hoenke, S.; Schulze, P.; Aragaw, W.; et al. Synthesis and characterization of phenylalanine amides active against Mycobacterium abscessus and other mycobacteria *Journal of Medicinal Chemistry* **2023**. DOI: 10.1021/acs.jmedchem.3c00009.

(2) Sheldrick, G. M. SHELXT - integrated space-group and crystal-structure determination. *Acta Crystallogr. A* **2015**, *71*, 3-8. DOI: 10.1107/S2053273314026370.

(3) Sheldrick, G. M. Crystal structure refinement with SHELXL. *Acta Crystallogr. C* **2015**, *71*, 3-8. DOI: 10.1107/S2053229614024218.

(4) Parsons, S.; Flack, H. D.; Wagner, T. Use of intensity quotients and differences in absolute structure refinement. *Acta Crystallogr B Struct Sci Cryst Eng Mater* **2013**, *69* (Pt 3), 249-259. DOI: 10.1107/S2052519213010014.

(5) Macrae, C. F.; Sovago, I.; Cottrell, S. J.; Galek, P. T. A.; McCabe, P.; Pidcock, E.; Platings, M.; Shields, G. P.; Stevens, J. S.; Towler, M.; et al. Mercury 4.0: from visualization to analysis, design and prediction. *J. Appl. Crystallogr.* **2020**, *53*, 226-235. DOI: 10.1107/S1600576719014092.

(6) Neese, F. Software Update: The ORCA Program System—Version 6.0. *WIREs Computational Molecular Science* **2025**, *15* (2). DOI: 10.1002/wcms.70019.
