# Supplementary material for: Cytochrome P450-Mediated Metabolism of Antimycobacterial Nα‑Aroyl‑N‑aryl-phenylalanine Amides
Source: ACS Infect Dis. 2026 Jun 2;12(6):1992–2003. doi: 10.1021/acsinfecdis.6c00100 (PMC13270519; doi:10.1021/acsinfecdis.6c00100)

checkCIF/PLATON report

Structure factors have been supplied for datablock(s) 15379sadabs_222

THIS REPORT IS FOR GUIDANCE ONLY. IF USED AS PART OF A REVIEW PROCEDURE FOR PUBLICATION, IT SHOULD NOT REPLACE THE EXPERTISE OF AN EXPERIENCED CRYSTALLOGRAPHIC REFEREE.

No syntax errors found. CIF dictionary Interpreting this report

Datablock: 15379sadabs_222

```
Bond precision:   C-C = 0.0050 A          Wavelength=0.71073

Cell:               a=4.8828(5)      b=18.634(2)      c=20.078(2)
                    alpha=90         beta=90          gamma=90
Temperature:        100 K
```

	Calculated	Reported
Volume	1826.8(3)	1826.8(3)
Space group	P 21 21 21	P 21 21 21
Hall group	P 2ac 2ab	P 2ac 2ab
Moiety formula	C22 H20 F N3 O2	C22 H20 F N3 O2
Sum formula	C22 H20 F N3 O2	C22 H20 F N3 O2
Mr	377.41	377.41
Dx,g cm-3	1.372	1.372
Z	4	4
Mu (mm-1)	0.097	0.097
F000	792.0	792.0
F000'	792.37	
h,k,lmax	7,26,28	6,26,28
Nref	5621[3248]	5585
Tmin,Tmax	0.996,0.998	0.994,0.998
Tmin'	0.991	

```
Correction method= # Reported T Limits: Tmin=0.994 Tmax=0.998
AbsCorr = GAUSSIAN
```

Data completeness= 1.72/0.99   Theta(max)= 30.655

R(reflections)= 0.0599(2761)   wR2(reflections)= 0.1305(5585)

S = 0.988   Npar= 276

The following ALERTS were generated. Each ALERT has the format
test-name_ALERT_alert-type_alert-level.
Click on the hyperlinks for more details of the test.

Alert level B

```
RINTA01_ALERT_3_B  The value of Rint is greater than 0.18
            Rint given   0.202
```

Author Response: The crystal was a small needle and weakly diffracting.

Alert level C

```
STRVA01_ALERT_4_C           Flack test results are ambiguous.
           From the CIF: _refine_ls_abs_structure_Flack    0.400
           From the CIF: _refine_ls_abs_structure_Flack_su    0.700
PLAT026_ALERT_3_C Ratio Observed / Unique Reflections (too) Low ..        49% Check
PLAT340_ALERT_3_C Low Bond Precision on  C-C Bonds ..............      0.005 Ang.
PLAT905_ALERT_3_C Negative K value in the Analysis of Variance ...     -1.438 Report
```

Alert level G

```
PLAT002_ALERT_2_G Number of Distance or Angle Restraints on AtSite         16 Note
PLAT020_ALERT_3_G The Value of Rint is Greater Than 0.12 .........      0.202 Report
PLAT032_ALERT_4_G Std. Uncertainty on Flack Parameter Value High .      0.700 Report
PLAT066_ALERT_1_G Predicted and Reported Tmin&Tmax Range Identical          ? Check
PLAT171_ALERT_4_G The CIF-Embedded .res File Contains EADP Records          7 Report
PLAT172_ALERT_4_G The CIF-Embedded .res File Contains DFIX Records          2 Report
PLAT174_ALERT_4_G The CIF-Embedded .res File Contains FLAT Records          1 Report
PLAT176_ALERT_4_G The CIF-Embedded .res File Contains SADI Records          5 Report
PLAT191_ALERT_3_G A Non-default SADI Restraint Value has been used     0.0400 Report
PLAT191_ALERT_3_G A Non-default SADI Restraint Value has been used     0.0400 Report
PLAT191_ALERT_3_G A Non-default SADI Restraint Value has been used     0.0400 Report
PLAT301_ALERT_3_G Main Residue  Disorder ..............(Resd    1)        25% Note
PLAT432_ALERT_2_G Short Inter X...Y Contact  F1'       ..C7       .       2.91 Ang.
                                        1/2+x,3/2-y,1-z  =      4_566 Check
PLAT480_ALERT_4_G Long H...A H-Bond Reported H19       ..F1       .       2.63 Ang.
PLAT791_ALERT_4_G Model has Chirality at C2          (Sohncke SpGr)          R Verify
PLAT860_ALERT_3_G Number of Least-Squares Restraints .............         13 Note
PLAT912_ALERT_4_G Missing # of FCF Reflections Above STh/L=  0.600         18 Note
PLAT969_ALERT_5_G The 'Henn et al.' R-Factor-gap value ...........      2.954 Note
              Predicted wR2: Based on SigI**2  4.42 or SHELX Weight 13.21
PLAT978_ALERT_2_G Number C-C Bonds with Positive Residual Density.          0 Info
```

---

0 **ALERT level A** = Most likely a serious problem - resolve or explain

```
 1 ALERT level B = A potentially serious problem, consider carefully
 4 ALERT level C = Check. Ensure it is not caused by an omission or oversight
19 ALERT level G = General information/check it is not something unexpected

 1 ALERT type 1 CIF construction/syntax error, inconsistent or missing data
 3 ALERT type 2 Indicator that the structure model may be wrong or deficient
10 ALERT type 3 Indicator that the structure quality may be low
 9 ALERT type 4 Improvement, methodology, query or suggestion
 1 ALERT type 5 Informative message, check
```

It is advisable to attempt to resolve as many as possible of the alerts in all categories. Often the minor alerts point to easily fixed oversights, errors and omissions in your CIF or refinement strategy, so attention to these fine details can be worthwhile. It is up to the individual to critically assess their own results and, if necessary, seek expert advice.

PLATON version of 26/09/2025; check.def file version of 20/09/2025

---

duplicate check

No duplication found

Datablock 15379sadabs_222 - ellipsoid plot

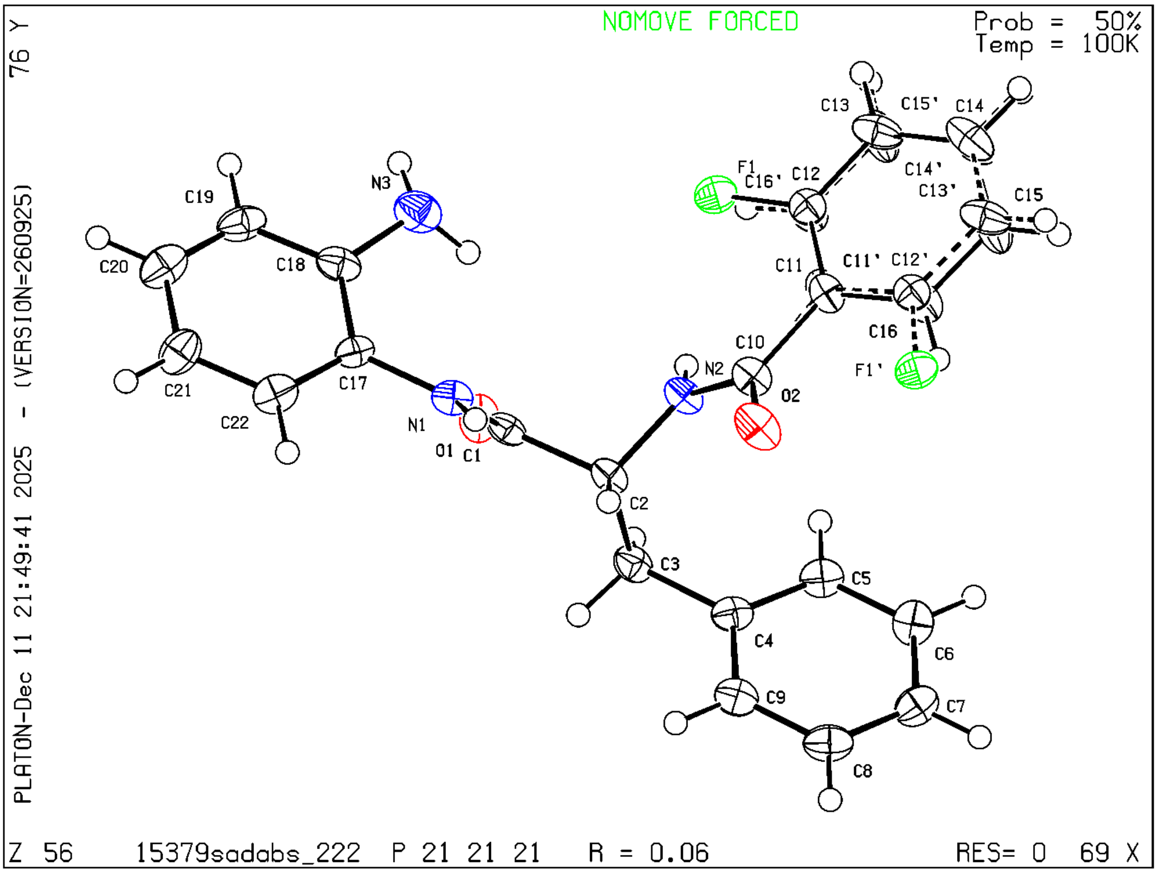

Supplement: Supplementary file 2 [file id6c00100_si_002.pdf]
